# Validation and genetic heritability estimation of known type 2 diabetes related variants in the Korean population

**DOI:** 10.5808/gi.21071

**Published:** 2021-12-31

**Authors:** Hye-Mi Jang, Mi Yeong Hwang, Bong-Jo Kim, Young Jin Kim

**Affiliations:** Division of Genome Science, Department of Precision Medicine, National Institute of Health, Cheongju 28159, Korea

**Keywords:** genome-wide association study, heritability, metabolic traits, single nucleotide polymorphism, type 2 diabetes

## Abstract

Genome-wide association studies (GWASs) facilitated the discovery of countless disease-associated variants. However, GWASs have mostly been conducted in European ancestry samples. Recent studies have reported that these European-based association results may reduce disease prediction accuracy when applied in non-Europeans. Therefore, previously reported variants should be validated in non-European populations to establish reliable scientific evidence for precision medicine. In this study, we validated known associations with type 2 diabetes (T2D) and related metabolic traits in 125,850 samples from a Korean population genotyped by the Korea Biobank Array (KBA). At the end of December 2020, there were 8,823 variants associated with glycemic traits, lipids, liver enzymes, and T2D in the GWAS catalog. Considering the availability of imputed datasets in the KBA genome data, publicly available East Asian T2D summary statistics, and the linkage disequilibrium among the variants (r^2^ < 0.2), 2,900 independent variants were selected for further analysis. Among these, 1,837 variants (63.3%) were statistically significant (p ≤ 0.05). Most of the non-replicated variants (n = 1,063) showed insufficient statistical power and decreased minor allele frequencies compared with the replicated variants. Moreover, most of known variants showed <10% genetic heritability. These results could provide valuable scientific evidence for future study designs, the current power of GWASs, and future applications in precision medicine in the Korean population.

## Introduction

Common diseases are a result of the complex interactions between genetic and environmental factors [1]. To date, several genetic studies have been conducted to identify diseases associated genetic variants and have used this gained knowledge as a clinical tool for disease prediction and prevention [2]. For the last decade, genome-wide association studies (GWASs) have been used as an efficient research tool for revealing numerous genetic variants associated with various diseases and traits [3]. Indeed, there were about 290K variants recorded in the NHGRI-EBI GWAS catalog [4].

Despite its great success, most GWASs have been conducted in European ancestry samples, and this Eurocentric bias may produce a significant reduction in disease prediction accuracy for non-Europeans [5,6]. This discrepancy might be caused by a difference in allele frequency distribution across populations and population-specific genetic effects [7,8]. Therefore, numerous studies have been conducted to validate previously reported diseases associated loci [9-12]. Here, we aimed to validate known associations with type 2 diabetes (T2D) and related metabolic traits in a large-scale East Asian population comprising of 125,850 Korean samples. Subsequently, this could establish reliable scientific evidence for disease prediction based on known T2D related associations in the Korean population.

The genetic components of complex diseases and traits were estimated as 30-70% based on family-based studies and statistical estimation using genome data. However, estimated heritability using validated loci showed only limited heritability, implying that there are more hidden genetic components to be revealed [13,14]. Since genetic variants are rapidly accumulating, it is valuable information to observe the current estimated genetic heritability status from known genetic variants. Therefore, we also estimated the genetic heritability from previously known variants associated with T2D and related quantitative traits.

## Methods

### Study subjects

Since 2001, the Korean Genome and Epidemiology Study (KoGES) has recruited 211,725 participants from three population-based cohorts including the KoGES_Ansan and Ansung study, the KoGES_Health EXAminee (HEXA) study, and the KoGES_CardioVascular disease Associations Study (CAVAS) [15]. The details of these studies have been described elsewhere [15]. All participants were aged between 40 and 69 years and provided written informed consent. Thousands of variables from these participants, including epidemiological surveys, physical examinations, and laboratory tests, were examined. This study was approved by the institutional review board at the Korea National Institute of Health, Republic of Korea.

### Phenotype measurements

Glycemic traits, liver enzymes, lipid traits, and T2D were considered T2D-related traits. Glycemic traits were fasting plasma glucose (FPG) and glycated hemoglobin (HbA1c). Liver enzymes included alanine aminotransferase (ALT), aspartate aminotransferase (AST), and γ-glutamyl transferase (GGT). Lipid traits included high-density lipoprotein (HDL), low-density lipoprotein (LDL), triglyceride (TG), and total cholesterol (TC). LDL was calculated using the Friedewald’s formula [16]. Participants taking medication or undergoing therapy likely influencing the traits were excluded from further analysis. All quantitative traits were transformed to follow an approximate normal distribution by inverse-variance or z-score normalization on residuals after regressing out age, gender, and recruitment area. TG was calculated using the log scale prior to transformation.

### Genotyping and quality control

In this study, quality controlled genotypes of 125,850 samples genotyped by the Korea Biobank Array (KBA) were used. The KBA was optimized for genome studies in the Korean population comprising of approximately 830K variants [17]. The detailed genotyping and quality control processes have been reported previously [12,17]. In brief, genotypes were called by batches containing about 3,000 to 8,000 samples considering the recruitment area. Chromosomal position was based on hg19. Samples were excluded based on the following criteria: gender discrepancy, low call rate (<97%), excessive heterozygosity, 2nd-degree related samples using KING v2 [18], outliers of principle component analysis by using FlashPCA [19]. Following quality control, low-quality variants were excluded if they had a high missing rate (> 5%), Hardy-Weinberg equilibrium failure (p < 10^-6^), and low minor allele frequency (MAF) (< 1%). As a result, 125,850 samples were used for further analysis.

### Retrieving previously associated variants

T2D-related variants were retrieved from a GWAS catalog database (https://www.ebi.ac.uk/gwas/) [4]. To avoid possible false-positive studies with <1,000 samples were excluded for further analysis. As of December 31, 2020, there were 8,823 variants associated with glycemic traits, lipid traits, liver enzymes, and T2D listed in the GWAS catalog. Chromosomal positions of the variants from GWAS catalog were based on hg38. Thus, to match the chromosomal positions with the association results, LiftOver from UCSC genome browser was used to convert hg38 positions to hg19 [20]. Using the p-value of the cataloged variants and linkage disequilibrium (LD) information from the 1,000 Genomes project phase 3 East Asians or Europeans [21], two different clumping analysis was conducted to obtain independent variants for East Asians or Europeans (if it was available in the KBA imputed genotype data). For clumping analysis, plink was used with options including --clump-p1 1 --clump-p2 1 --clump-r^2^ 0.l --clump-kb 500. Two sets of independently associated variants for East Asians or Europeans were then merged. Subsequently, 2,900 variants were selected as independent variants and used for further analysis.

### Statistical analysis

The 8,823 variants associated with T2D related traits were imputed if they were not available in the KBA genotype data. KBA genotype data was phased using Eagle v2.3 [22] and imputed using Impute v4 (https://jmarchini.org/software/) [23] with a merged reference panel of 2,504 samples of 1,000 Genomes project phase 3 and 397 Korean whole genome sequencing data [17]. Linear regression analysis using transformed traits was performed using EPACTS v3.4.6 (http://genome.sph.umich.edu/wiki/EPACTS). T2D association results were assessed from publicly available summary statistics of previously conducted East Asian meta-analysis study [8]. R statistics program (version 4.0.5; https://www.r-project.org) was used to visualize the results. For lipids and T2D, we used LD score regression (LDSC) from bigsnpr R package to estimate genetic heritability of each trait using all variants matched with HapMap phase 3 within ±500 kb window of the independent variants [24,25]. For the other traits, heritability was estimated as the sum of estimated heritability for each variant using the effect size of the variant and variance of the associated trait. LDSC could not reliably calculate the correlation matrix of relatively small number of loci.

## Results

**Fig. 1.**
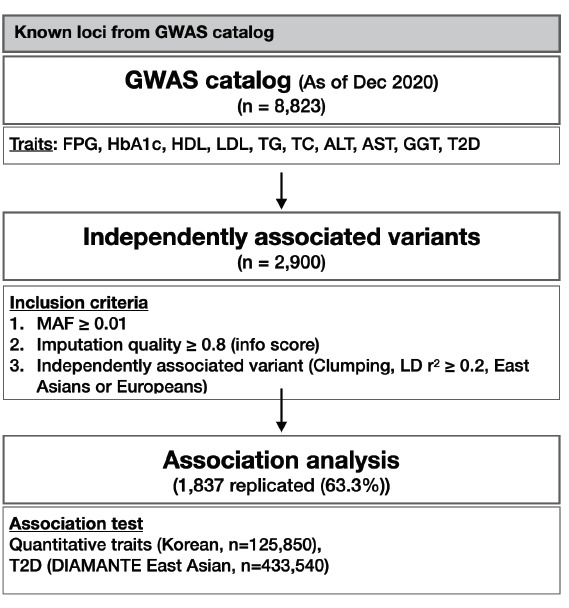
Schematic representation of analysis flow. GWAS, genome-wide association study; FPG, fasting plasma glucose; HbA1c, glycated hemoglobin; HDL, high-density lipoprotein; LDL, low-density lipoprotein; TG, triglyceride; TC, total cholesterol; ALT, alanine aminotransferase; AST, aspartate aminotransferase; GGT, γ-glutamyl transferase; T2D, type 2 diabetes; MAF, minor allele frequency; LD, linkage disequilibrium.

At the end of 2020, there were 8,823 variants associated with T2D-related traits including glycemic traits (FPG and HbA1c), lipids (HDL, LDL, TG, and TC), liver enzymes (AST, ALT, and GGT), and T2D in the GWAS catalog database. Because various studies have reported index variants closely located to each other, only independent variants with a high imputation quality score (≥0.8) were selected for further analysis by the clumping method, considering a linkage disequilibrium of r^2^ < 0.2 either in East Asian or European based on 1,000 genomes project data. Subsequently, 2,900 independent variants were identified and used in the association analysis. The overall analysis scheme is summarized in Fig. 1.

T2D-related metabolic traits, and the association results of the T2D variants were retrieved from a publicly available summary statistics of the DIAMANTE East Asians association study [8]. Overall, 1,837 variants (63.3%) were statistically significant (p ≤ 0.05) (Table 1, Supplementary Table 1). The replication rate was the lowest for TG (51.9%) and the highest for HbA1c (84.5%).

**Fig. 2.**
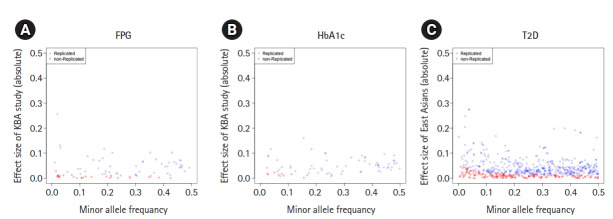
Effect sizes and minor allele frequency of variants associated with glycemic traits and type 2 diabetes. The X-axis represents the minor allele frequency of the variants. The Y-axis represents the absolute scale of effect sizes of variants. Each circle indicates a variant. Variants were colored in blue if they were replicated in this study (p ≤ 0.05) and colored in red otherwise (p > 0.05). (A) Fasting plasma glucose (FPG). (B) Glycated hemoglobin (HbA1c). (C) Type 2 diabetes (T2D).

The failure of replication in the Korean population could be due to insufficient statistical power and differences in genetic architectures. To study the possible reasons for this failure, we analyzed the statistical power and effect size distribution by MAF for the unvalidated variants (n = 1,063). The estimated statistical power with alpha of 0.05 is summarized in Table 2. Most of variants associated with T2D-related traits presented with insufficient statistical power, ranging from 0.36 for HbA1c to 0.68 for TG. However, 83 of 183 unvalidated T2D variants (45.3%) presented with enough statistical power (>80%), implicating a possible difference in genetic architecture across populations. Effect sizes by MAF of 1,063 non-replicated variants (n = 1,063) were plotted by each trait (Fig. 2, Supplementary Figs. 1 and 2). As expected, the effect size increased as MAF decreased. The effect sizes of non-replicated variants were closed to zero. Non-replicated variants were populated at a lower MAF than replicated variants, especially for glycemic traits and T2D.

To observe the current status of estimated genetic heritability from known genetic variants, genetic heritability was estimated using the effect sizes from known genetic variants (Table 3). The estimated heritability was the lowest for AST and ALT (1.32%) and the highest for TG (20.37%). However, estimated heritability was <10% for most traits implying that further analysis is needed to identify the hidden genetic components of T2D related traits.

## Discussion

In this study, associations of 2,900 known T2D related variants were validated in the 125,870 Korean samples. From these known variants, 1,837 (63.3%) were replicated, however, 1,063 variants were not replicated due to insufficient statistical power and difference in genetic architecture across populations. Most non-replicated variants showed insufficient statistical power (<0.8) and a relatively lower MAF than the replicated variants. Additionally, we estimated the current status of genetic heritability using the known variants. The genetic heritability from known loci was mostly less than 10% implying that there is a great portion of missing heritability for T2D-related variants. These results could provide valuable scientific evidence for future study design, the current power of GWAS, and future applications to precision medicine in the Korean population.

Despite these valuable findings of the current study, there were a few limitations and careful interpretation is required. First, given the insufficient statistical power from a limited number of sample size compared to that in previous studies conducted in Europeans [5-7], an association study with a larger sample size is warranted to achieve sufficient statistical power to investigate known associations. Second, we used the threshold as p ≤ 0.05. However, multiple testing considering number of independent variants is more reliable to avoid the inflation of false-positives. Third, independent variants were selected to avoid missing targets of previous reports based on linkage disequilibrium patterns from both East Asian and European populations. Therefore, high non-replicability might be caused by the inclusion of index variants from previous studies conducted in European populations. Finally, heritability was estimated using LDSC with all variants within the known loci or the sum of estimated heritability of independent variants. Some of traits with relatively small number of loci were unable to estimate the heritability using LDSC. Therefore, further study is warranted to estimate an accurate heritability accounting for genetic architecture within the loci.

Most of the non-replicated variants showed relatively less frequency compared to the replicated variants. To validate these variants in the Korean population, an immense sample size (up to millions) is required to obtain sufficient statistical power based on the estimated statistical power of non-replicated variants in this study. Insufficient statistical power from less frequent variants is a common problem in single ancestry studies [26,27]. Therefore, a trans-ethnic meta-analysis would be an adequate approach to identify hidden variants.

## Figures and Tables

**Table 1.** Summary of replication results

Category	No. of variants	Replicated	Not replicated (p > 0.05)	
		(p ≤ 0.05)	Total	%
Liver enzymes				
ALT	35	22	13	62.9
AST	29	23	6	79.3
GGT	64	53	11	82.8
Lipid traits				
HDL	662	383	279	57.9
LDL	484	286	198	59.1
TG	566	294	272	51.9
TC	297	234	63	78.8
Glycemic traits				
FPG	80	53	27	66.3
HbA1c	71	60	11	84.5
T2D	612	429	183	70.1
Total	2,900	1,837	1,063	63.3

ALT, alanine aminotransferase; AST, aspartate aminotransferase; GGT, γ-glutamyl transferase; HDL, high-density lipoprotein; LDL, low-density lipoprotein; TG, triglyceride; TC, total cholesterol; FPG, fasting plasma glucose; HbA1c, glycated hemoglobin; T2D, type 2 diabetes.

**Table 2.** Estimated statistical power of non-replicated variants

Category	No. of non-replicated variants	Statistical power		% (power > 80%)
		Min	Max	
Liver enzymes				
ALT	13	0.0501	0.4714	0
AST	6	0.0543	0.5133	0
GGT	11	0.0533	0.4250	0
Lipid traits				
HDL	279	0.0500	0.5325	0
LDL	198	0.0500	0.3633	0
TG	272	0.0500	0.6796	0
TC	63	0.0503	0.5133	0
Glycemic traits				
FPG	27	0.0500	0.3633	0
HbA1c	11	0.0510	0.4877	0
T2D	183	0.0501	0.9994	45.36

ALT, alanine aminotransferase; AST, aspartate aminotransferase; GGT, γ -glutamyl transferase; HDL, high-density lipoprotein; LDL, low-density lipoprotein; TG, triglyceride; TC, total cholesterol; FPG, fasting plasma glucose; HbA1c, glycated hemoglobin; T2D, type 2 diabetes.

**Table 3.** Estimated genetic heritability of known variants

Category	No. of variants	Sample size	Estimated heritability (%)
Liver enzymes			
ALT	13	109,068	1.32
AST	6	109,230	1.32
GGT	11	102,729	6.62
Lipid traits			
HDL	279	120,559	19.99
LDL	198	77,363	20.37
TG	272	120,377	14.03
TC	63	120,561	12.28
Glycemic traits			
FPG	27	109,942	6.17
HbA1c	11	51,385	8.24
T2D	183	433,540	7.69

ALT, alanine aminotransferase; AST, aspartate aminotransferase; GGT, γ -glutamyl transferase; HDL, high-density lipoprotein; LDL, low-density lipoprotein; TG, triglyceride; TC, total cholesterol; FPG, fasting plasma glucose; HbA1c, glycated hemoglobin; T2D, type 2 diabetes.

## Supplementary Materials

Supplementary Table 1. Replication results of known type 2 diabetes related variantsa

Supplementary Fig. 1. Effect sizes and MAF of variants (liver enzymes)

Supplementary Fig. 2. Effect sizes and MAF of variants (lipid traits)
