# Supplementary material for: Validation and genetic heritability estimation of known type 2 diabetes related variants in the Korean population
Source: Genomics Inform. 2021 Dec 31;19(4):e37. doi: 10.5808/gi.21071 (PMC8752982; doi:10.5808/gi.21071)
Supplement: Supplementary Table 1. — Replication results of known type 2 diabetes related variantsa [file gi-21071suppl1.pdf]

Supplementary Table 1. Replication results of known type 2 diabetes related variants

Traits	CHR	POS	EA	NEA	EAF	N
HDL	1	935222	A	C	0.7830	120559
HDL	1	23710475	T	C	0.6451	120559
HDL	1	25022314	A	G	0.2251	120559
HDL	1	28406047	T	G	0.5085	120559
HDL	1	29567412	T	C	0.4768	120559
HDL	1	40022777	C	G	0.8901	120559
HDL	1	40035686	T	C	0.1045	120559
HDL	1	63156043	A	G	0.1936	120559
HDL	1	66073952	T	C	0.8431	120559
HDL	1	93634590	A	C	0.6426	120559
HDL	1	93846653	A	T	0.3460	120559
HDL	1	107549245	A	G	0.2976	120559
HDL	1	109817192	A	G	0.9395	120559
HDL	1	109818530	T	C	0.9404	120559
HDL	1	110267651	T	C	0.1086	120559
HDL	1	110470764	A	G	0.3206	120559
HDL	1	150940625	T	G	0.9223	120559
HDL	1	156700651	T	G	0.8990	120559
HDL	1	161163037	T	C	0.0743	120559
HDL	1	161614490	T	G	0.1723	46421
HDL	1	171455322	T	C	0.0742	120559
HDL	1	172346548	A	G	0.9058	120559
HDL	1	178515312	A	G	0.4947	120559
HDL	1	182150978	A	C	0.2919	120559
HDL	1	182154990	T	C	0.7075	120559
HDL	1	183100563	T	G	0.6670	120559
HDL	1	184049978	T	C	0.3951	120559
HDL	1	201771326	A	G	0.2302	120559
HDL	1	203519194	T	C	0.5220	120559
HDL	1	205041542	T	C	0.5763	120559
HDL	1	205677148	A	G	0.5034	120559
HDL	1	205719532	A	G	0.5021	120559
HDL	1	206626828	C	G	0.7647	120559
HDL	1	212422567	CA	C	0.4988	120559
HDL	1	212422567	A	C	0.2203	120559
HDL	1	214992980	C	G	0.8155	120559
HDL	1	219664030	A	G	0.6296	120559
HDL	1	219672376	C	G	0.7318	120559
HDL	1	220970028	A	G	0.1568	120559
HDL	1	228088833	T	C	0.2980	120559
HDL	1	230294916	T	C	0.1607	120559
HDL	1	230297778	A	T	0.7491	120559
HDL	1	230416399	T	G	0.8676	120559
HDL	1	234853268	A	C	0.2942	120559
HDL	2	272203	T	C	0.1946	120559
HDL	2	3634753	T	C	0.7018	120559
HDL	2	3640142	T	C	0.2945	120559

HDL	2	9154942	T	C	0.7732	120559
HDL	2	20371380	A	G	0.3399	120559
HDL	2	21221035	A	C	0.7327	120559
HDL	2	21225281	T	C	0.2580	120559
HDL	2	21263900	A	G	0.1121	120559
HDL	2	30478453	T	G	0.7527	120559
HDL	2	42602387	T	C	0.3122	120559
HDL	2	46642249	A	G	0.5400	120559
HDL	2	48484467	T	C	0.5612	120559
HDL	2	58950363	T	G	0.1420	120559
HDL	2	58975143	T	C	0.6168	120559
HDL	2	59335104	T	G	0.3296	120559
HDL	2	65279414	A	G	0.6689	120559
HDL	2	65282708	C	G	0.3296	120559
HDL	2	66673862	A	C	0.6759	120559
HDL	2	85543222	T	C	0.9197	120559
HDL	2	100796850	T	C	0.5156	120559
HDL	2	111664756	A	G	0.7189	120559
HDL	2	111822002	C	G	0.2912	120559
HDL	2	111894720	T	C	0.8775	120559
HDL	2	128593977	A	G	0.7709	120559
HDL	2	135263081	A	G	0.0606	120559
HDL	2	147871114	A	G	0.3670	120559
HDL	2	156926963	T	C	0.5220	120559
HDL	2	161265910	T	C	0.2539	120559
HDL	2	165528876	T	C	0.1003	120559
HDL	2	173926771	T	C	0.6982	120559
HDL	2	178126546	C	G	0.5399	120559
HDL	2	203477868	A	C	0.0460	120559
HDL	2	211540507	A	C	0.1786	120559
HDL	2	226306993	T	C	0.6129	120559
HDL	2	227019416	T	G	0.0500	120559
HDL	2	227099180	T	C	0.9436	120559
HDL	2	227116365	A	G	0.0616	120559
HDL	2	227229344	C	G	0.2958	120559
HDL	2	230020220	T	C	0.1728	120559
HDL	2	242237902	T	C	0.5226	120559
HDL	3	11412604	T	C	0.3398	120559
HDL	3	12327431	T	G	0.9785	120559
HDL	3	12351223	A	G	0.7623	120559
HDL	3	12379351	A	G	0.9502	120559
HDL	3	12737231	A	C	0.0463	120559
HDL	3	15846011	T	C	0.1236	120559
HDL	3	24293001	T	C	0.8608	120559
HDL	3	36960660	A	C	0.6096	120559
HDL	3	47061183	A	G	0.2586	120559
HDL	3	47097985	A	G	0.2584	120559
HDL	3	48767877	A	G	0.7654	120559

HDL	3	50024038	A	C	0.8370	120559
HDL	3	50041313	T	C	0.8342	120559
HDL	3	51926817	A	C	0.4964	120559
HDL	3	52529899	A	T	0.0234	120559
HDL	3	53139506	T	C	0.7556	120559
HDL	3	108867705	T	C	0.9377	120559
HDL	3	114484372	T	C	0.9631	120559
HDL	3	119529113	T	G	0.9322	120559
HDL	3	119560606	T	C	0.5455	120559
HDL	3	123049938	A	C	0.0334	120559
HDL	3	131642852	T	C	0.9104	120559
HDL	3	131751775	A	C	0.9076	120559
HDL	3	133478557	T	G	0.3048	120559
HDL	3	135798658	T	C	0.1245	120559
HDL	3	135880410	A	T	0.9900	120559
HDL	3	150146399	A	T	0.0324	120559
HDL	3	152171870	C	G	0.7433	120559
HDL	3	156795414	T	G	0.5315	120559
HDL	3	156798732	A	G	0.4654	120559
HDL	3	160025287	T	C	0.1710	120559
HDL	3	183976103	T	C	0.0565	120559
HDL	3	185822353	T	G	0.9751	120559
HDL	3	185822774	A	G	0.5225	120559
HDL	3	185834499	A	T	0.0240	120559
HDL	3	185878419	A	G	0.0442	120559
HDL	3	185931174	T	C	0.9675	120559
HDL	3	196073072	A	G	0.8043	120559
HDL	4	858332	T	C	0.1055	120559
HDL	4	951947	T	C	0.8437	120559
HDL	4	954311	T	C	0.4125	120559
HDL	4	970112	A	G	0.5801	120559
HDL	4	2250109	T	C	0.6806	120559
HDL	4	17924734	T	C	0.9041	120559
HDL	4	24626903	T	G	0.2377	120559
HDL	4	37151533	T	C	0.5370	120559
HDL	4	39700173	T	C	0.7082	120559
HDL	4	41254862	T	C	0.2286	120559
HDL	4	55526251	T	G	0.1414	120559
HDL	4	69349018	A	C	0.8902	120559
HDL	4	69361445	T	C	0.8845	120559
HDL	4	69533217	A	C	0.5059	120559
HDL	4	76572191	A	C	0.8351	120559
HDL	4	83917037	A	G	0.4736	120559
HDL	4	87862396	C	G	0.6041	120559
HDL	4	87982876	C	G	0.5577	120559
HDL	4	88022709	T	C	0.3574	120559
HDL	4	89723065	A	G	0.7034	120559
HDL	4	91245506	T	C	0.1802	120559

HDL	4	99788480	T	C	0.6450	120559
HDL	4	100014805	A	G	0.1939	120559
HDL	4	100270256	T	C	0.9872	120559
HDL	4	100517324	T	C	0.2342	120559
HDL	4	104253889	A	G	0.7790	120559
HDL	4	106081636	T	C	0.3607	120559
HDL	4	120034116	A	G	0.8254	120559
HDL	4	146403165	A	G	0.5652	120559
HDL	4	151090031	T	C	0.7221	120559
HDL	4	157706904	T	C	0.2938	120559
HDL	5	1282414	A	G	0.3611	120559
HDL	5	39522481	T	G	0.7516	120559
HDL	5	52664796	A	G	0.6641	120559
HDL	5	53274467	A	G	0.8969	120559
HDL	5	53283630	A	T	0.8815	120559
HDL	5	53298025	A	G	0.4714	120559
HDL	5	55806751	A	G	0.4868	120559
HDL	5	55808475	T	C	0.5139	120559
HDL	5	55860781	A	G	0.9084	120559
HDL	5	55861786	T	C	0.0920	120559
HDL	5	59391636	T	C	0.9762	120559
HDL	5	67714246	A	G	0.5422	120559
HDL	5	72926514	A	G	0.1716	120559
HDL	5	75003678	T	C	0.4333	120559
HDL	5	75696662	T	C	0.9746	120559
HDL	5	78421959	A	G	0.2775	120559
HDL	5	103944020	T	G	0.3752	120559
HDL	5	108656635	A	G	0.9621	120559
HDL	5	112188456	T	C	0.2608	120559
HDL	5	118729286	T	C	0.1393	120559
HDL	5	124057584	A	G	0.2475	120559
HDL	5	127350549	T	C	0.3352	120559
HDL	5	134444982	A	C	0.1939	120559
HDL	5	149211868	A	G	0.2964	120559
HDL	5	153213649	A	G	0.0416	120559
HDL	5	158003020	A	G	0.7505	120559
HDL	5	158022041	A	G	0.7465	120559
HDL	5	158622532	A	C	0.3932	120559
HDL	5	170459675	A	G	0.9204	120559
HDL	5	170612546	A	G	0.8794	120559
HDL	5	176637576	T	C	0.5111	120559
HDL	5	180226673	A	C	0.8708	120559
HDL	6	7255610	A	G	0.9707	120559
HDL	6	16825137	A	G	0.8526	120559
HDL	6	20304563	A	T	0.2665	120559
HDL	6	22743403	A	G	0.7215	120559
HDL	6	26090270	C	G	0.0615	120559
HDL	6	29829636	A	G	0.0304	120559

HDL	6	31195996	A	G	0.2742	120559
HDL	6	31300286	A	G	0.7046	120559
HDL	6	32379383	C	G	0.2093	120559
HDL	6	32500957	A	T	0.8616	120559
HDL	6	32560883	A	G	0.3633	120559
HDL	6	32632367	T	C	0.5057	120559
HDL	6	32669373	T	C	0.0521	120559
HDL	6	33717770	T	C	0.1663	120559
HDL	6	34188892	A	C	0.9735	120559
HDL	6	34595543	T	C	0.6391	120559
HDL	6	35142899	T	C	0.7185	120559
HDL	6	39146230	T	C	0.8421	120559
HDL	6	41991740	T	C	0.9570	120559
HDL	6	42902508	T	C	0.6933	120559
HDL	6	42928461	T	C	0.1338	120559
HDL	6	43757896	A	C	0.5590	120559
HDL	6	43758873	A	G	0.7669	120559
HDL	6	47369202	A	C	0.1462	120559
HDL	6	75455385	T	C	0.1864	120559
HDL	6	98574560	T	C	0.4088	120559
HDL	6	109510972	A	G	0.3957	120559
HDL	6	111834954	T	C	0.0109	120559
HDL	6	116322349	T	C	0.7459	120559
HDL	6	127414801	T	C	0.5610	120559
HDL	6	127452639	C	G	0.4723	120559
HDL	6	131897278	T	C	0.2794	120559
HDL	6	137082948	A	G	0.5316	120559
HDL	6	139226630	T	G	0.6237	120559
HDL	6	139831757	T	C	0.7333	120559
HDL	6	139834012	T	G	0.7345	120559
HDL	6	153431125	T	C	0.8499	120559
HDL	6	153459444	A	T	0.0391	120559
HDL	6	154450989	A	G	0.9056	120559
HDL	6	163740322	A	G	0.1894	120559
HDL	7	1051664	T	C	0.4574	120559
HDL	7	1114381	T	C	0.0269	120559
HDL	7	6449272	A	G	0.1161	120559
HDL	7	6456091	T	C	0.1956	120559
HDL	7	6461310	T	C	0.2341	120559
HDL	7	12224708	T	C	0.5099	120559
HDL	7	12269417	C	G	0.3478	120559
HDL	7	15889360	T	C	0.4719	120559
HDL	7	17284577	T	C	0.6022	120559
HDL	7	17914600	T	C	0.5189	120559
HDL	7	17920253	C	G	0.5188	120559
HDL	7	17992014	A	G	0.3638	120559
HDL	7	26370190	A	G	0.4323	120559
HDL	7	26397239	A	C	0.8099	120559

HDL	7	36193142	T	G	0.8077	120559
HDL	7	36292925	A	G	0.8109	120559
HDL	7	38277792	A	G	0.4769	120559
HDL	7	50289669	A	C	0.3418	120559
HDL	7	50305863	T	G	0.5980	120559
HDL	7	73039406	C	G	0.8964	120559
HDL	7	95011948	T	C	0.9164	120559
HDL	7	101737327	T	G	0.9550	120559
HDL	7	106962948	A	G	0.1343	120559
HDL	7	109103912	A	G	0.2202	120559
HDL	7	127851628	A	G	0.1094	120559
HDL	7	130430930	T	C	0.2922	120559
HDL	7	130432538	A	T	0.7080	120559
HDL	7	134669523	T	C	0.1931	120559
HDL	7	139099813	T	G	0.8104	120559
HDL	7	150327424	A	C	0.1754	120559
HDL	7	150331377	A	G	0.1762	120559
HDL	7	150537635	A	G	0.6305	120559
HDL	8	4164013	A	T	0.8660	120559
HDL	8	6599005	T	C	0.2730	120559
HDL	8	6601492	T	G	0.7048	120559
HDL	8	9105172	A	G	0.1368	120559
HDL	8	9183358	A	G	0.0108	120559
HDL	8	9187242	A	G	0.0105	120559
HDL	8	9859913	T	C	0.6763	120559
HDL	8	10491684	C	G	0.0731	120559
HDL	8	10643164	T	C	0.0143	120559
HDL	8	12623463	A	G	0.2707	120559
HDL	8	13536115	T	C	0.0419	120559
HDL	8	14291955	A	G	0.7495	120559
HDL	8	19662937	T	C	0.0611	120559
HDL	8	19810787	A	C	0.1324	120559
HDL	8	19817476	T	G	0.9032	120559
HDL	8	19819328	T	C	0.8751	120559
HDL	8	19824667	T	C	0.2097	120559
HDL	8	19928013	A	T	0.2505	120559
HDL	8	19942181	T	G	0.2015	120559
HDL	8	25464670	T	G	0.2935	120559
HDL	8	29360305	A	C	0.2771	120559
HDL	8	34406540	T	C	0.3736	120559
HDL	8	38782627	T	C	0.9636	120559
HDL	8	64653461	T	C	0.9808	120559
HDL	8	71260460	A	G	0.4133	120559
HDL	8	71338185	T	C	0.3012	120559
HDL	8	72395582	A	AAGCCCT	0.1186	120559
HDL	8	73439070	A	G	0.3006	120559
HDL	8	95997165	T	C	0.0481	120559
HDL	8	103876780	T	C	0.1756	120559

HDL	8	105982209	C	G	0.1646	120559
HDL	8	106590684	A	G	0.4797	120559
HDL	8	116599199	T	G	0.2057	120559
HDL	8	116601894	T	C	0.7943	120559
HDL	8	121867780	T	C	0.4021	120559
HDL	8	126480367	T	C	0.6798	120559
HDL	8	126495818	T	C	0.1853	120559
HDL	8	126507389	A	C	0.7123	120559
HDL	8	126629328	T	G	0.5619	120559
HDL	8	144302570	T	C	0.7958	120559
HDL	8	144303418	A	G	0.3489	120559
HDL	8	144496772	A	C	0.8757	120559
HDL	9	1033773	C	G	0.5699	120559
HDL	9	13719203	A	G	0.4565	120559
HDL	9	15303583	A	G	0.9704	120559
HDL	9	15304782	A	C	0.9696	120559
HDL	9	16728532	A	C	0.6870	120559
HDL	9	17295541	C	G	0.7725	120559
HDL	9	19360566	A	G	0.0192	120559
HDL	9	28410683	T	C	0.7892	120559
HDL	9	28414339	A	G	0.7893	120559
HDL	9	86481873	A	C	0.2581	120559
HDL	9	92177897	A	G	0.8810	120559
HDL	9	94010298	A	G	0.0493	120559
HDL	9	95382297	T	C	0.5728	120559
HDL	9	100809135	T	C	0.2106	120559
HDL	9	107515814	T	C	0.2546	120559
HDL	9	107562557	A	G	0.6556	120559
HDL	9	107579880	T	C	0.0599	120559
HDL	9	107589567	T	C	0.0250	120559
HDL	9	107589744	T	C	0.7121	120559
HDL	9	107594364	A	G	0.9243	120559
HDL	9	107647019	T	C	0.0737	120559
HDL	9	107661742	A	C	0.7471	120559
HDL	9	107665739	A	G	0.2490	120559
HDL	9	107684230	T	G	0.6965	120559
HDL	9	112575692	C	G	0.3109	120559
HDL	9	117140082	T	C	0.5117	120559
HDL	9	123631225	A	G	0.5289	120559
HDL	9	131562232	A	G	0.6468	120559
HDL	9	135033545	A	G	0.7510	120559
HDL	9	136149830	A	G	0.2601	120559
HDL	9	136919416	A	G	0.3404	120559
HDL	10	8576206	A	G	0.7800	120559
HDL	10	17265447	T	C	0.3156	120559
HDL	10	33448764	T	G	0.1211	120559
HDL	10	33647091	A	G	0.6633	120559
HDL	10	34015681	A	T	0.6899	120559

HDL	10	46018831	A	G	0.9123	120559
HDL	10	61409469	A	G	0.7836	120559
HDL	10	65052205	T	C	0.6169	120559
HDL	10	74711376	T	C	0.6577	120559
HDL	10	76847490	T	C	0.9443	120559
HDL	10	80954251	A	G	0.3258	120559
HDL	10	80955067	T	C	0.3831	120559
HDL	10	88511326	T	G	0.7188	120559
HDL	10	94839724	T	G	0.7280	120559
HDL	10	95309022	T	C	0.9057	120559
HDL	10	99769388	T	C	0.7507	120559
HDL	10	101912064	T	C	0.9435	120559
HDL	10	102027407	T	C	0.0558	120559
HDL	10	113913222	T	C	0.3092	120559
HDL	10	113940329	T	C	0.3084	120559
HDL	10	113977211	A	G	0.3195	120559
HDL	10	114019830	A	G	0.3749	120559
HDL	10	115789375	T	C	0.7919	120559
HDL	10	115795236	A	G	0.0098	120559
HDL	10	121659811	A	G	0.3174	120559
HDL	10	122859177	T	G	0.0711	120559
HDL	10	122898697	T	C	0.3361	120559
HDL	10	126354554	C	G	0.4118	120559
HDL	10	126696496	T	C	0.6579	120559
HDL	11	2936952	A	G	0.0717	120559
HDL	11	2958538	T	C	0.3190	120559
HDL	11	5701074	A	C	0.0684	120559
HDL	11	10380828	A	C	0.7945	120559
HDL	11	10388782	A	G	0.4731	120559
HDL	11	13355770	T	C	0.1151	120559
HDL	11	14504463	A	C	0.8692	120559
HDL	11	14852490	A	G	0.4984	120559
HDL	11	18067020	CA	C	0.1716	120559
HDL	11	27675712	T	C	0.7656	120559
HDL	11	30435051	T	G	0.0518	120559
HDL	11	43628749	T	C	0.8198	120559
HDL	11	46743247	T	C	0.4071	120559
HDL	11	47246397	A	G	0.2715	120559
HDL	11	47324666	A	T	0.6496	120559
HDL	11	47752775	A	G	0.6858	120559
HDL	11	48518893	A	G	0.4462	120559
HDL	11	49616839	A	G	0.1946	120559
HDL	11	51512090	T	C	0.8720	120559
HDL	11	54846440	A	C	0.8529	120559
HDL	11	55451313	A	G	0.1808	120559
HDL	11	56156771	C	G	0.8714	120559
HDL	11	56964224	A	G	0.0674	120559
HDL	11	58162382	A	G	0.1077	120559

HDL	11	58376120	A	G	0.1170	120559
HDL	11	61592362	A	G	0.6943	120559
HDL	11	61609750	T	C	0.3174	120559
HDL	11	62316195	C	G	0.9568	120559
HDL	11	63877163	T	C	0.3102	120559
HDL	11	65391317	A	G	0.5071	120559
HDL	11	65473798	A	C	0.2685	120559
HDL	11	66066993	T	G	0.3665	120559
HDL	11	68597886	T	G	0.1770	120559
HDL	11	68703959	A	G	0.7933	120559
HDL	11	75451281	A	T	0.7339	120559
HDL	11	75473715	T	C	0.7406	120559
HDL	11	76266172	T	C	0.3703	120559
HDL	11	95320808	T	C	0.9251	120559
HDL	11	103871404	A	C	0.7233	120559
HDL	11	109963745	C	G	0.1256	120559
HDL	11	110012143	T	G	0.8961	120559
HDL	11	116586283	T	C	0.2345	120559
HDL	11	116611827	A	T	0.2236	120559
HDL	11	116617240	A	C	0.8036	120559
HDL	11	116648917	C	G	0.7817	120559
HDL	11	116657667	T	C	0.0792	120559
HDL	11	116667545	T	C	0.0532	120559
HDL	11	116705516	A	G	0.2407	120559
HDL	11	116728630	C	G	0.1200	120559
HDL	11	116784376	T	G	0.1493	120559
HDL	11	116884789	A	G	0.1457	120559
HDL	11	117075566	T	C	0.1193	120559
HDL	11	117085270	A	C	0.5458	120559
HDL	11	118346863	A	G	0.4115	120559
HDL	11	118941596	A	G	0.1323	120559
HDL	11	122514403	T	C	0.1468	120559
HDL	11	122526601	T	C	0.1455	120559
HDL	11	126225876	C	G	0.8830	120559
HDL	11	126316025	A	G	0.8321	120559
HDL	12	6731818	A	G	0.6876	120559
HDL	12	6861043	T	C	0.4404	120559
HDL	12	7691134	A	G	0.6197	120559
HDL	12	7725904	T	TAA	0.3327	120559
HDL	12	7774892	A	G	0.6830	120559
HDL	12	9082581	A	G	0.7107	120559
HDL	12	20474706	A	G	0.8548	120559
HDL	12	20579392	C	G	0.0712	120559
HDL	12	26440698	A	G	0.2810	120559
HDL	12	26474867	A	G	0.2517	120559
HDL	12	33459554	A	G	0.9653	120559
HDL	12	33715933	T	C	0.9206	120559
HDL	12	51157863	T	G	0.3324	120559

HDL	12	53752692	A	G	0.2394	120559
HDL	12	53796744	T	C	0.0927	120559
HDL	12	56368078	A	G	0.5350	120559
HDL	12	56379060	A	T	0.7701	120559
HDL	12	57391292	T	C	0.1374	120559
HDL	12	57839173	T	G	0.0917	120559
HDL	12	57843711	A	G	0.0939	120559
HDL	12	67651972	C	G	0.7202	120559
HDL	12	71106648	T	C	0.5451	120559
HDL	12	84017043	A	G	0.3661	120559
HDL	12	101873956	T	C	0.3913	120559
HDL	12	101888063	T	C	0.0336	120559
HDL	12	107128090	A	T	0.7061	120559
HDL	12	109871179	A	G	0.5024	120559
HDL	12	109950144	A	G	0.6847	120559
HDL	12	110116872	A	G	0.5347	120559
HDL	12	110368201	A	G	0.2273	120559
HDL	12	111718231	A	C	0.1819	120559
HDL	12	121405210	T	C	0.5095	120559
HDL	12	123159262	T	C	0.0353	120559
HDL	12	123199410	A	G	0.5486	120559
HDL	12	123775127	A	G	0.2964	120559
HDL	12	123895906	C	G	0.7042	120559
HDL	12	124475156	A	G	0.8911	120559
HDL	12	124505444	T	C	0.4454	120559
HDL	12	124637757	T	C	0.1996	120559
HDL	12	125086417	T	C	0.1017	120559
HDL	12	125259888	A	G	0.0997	120559
HDL	12	125265201	A	G	0.3794	120559
HDL	12	125283766	T	C	0.7546	120559
HDL	12	125315647	A	G	0.3123	120559
HDL	12	125325778	T	C	0.0626	120559
HDL	12	125338529	T	C	0.5994	120559
HDL	12	125353810	A	T	0.1994	120559
HDL	12	125380232	A	G	0.7937	120559
HDL	12	125434716	A	G	0.6135	120559
HDL	13	49513352	A	G	0.6098	120559
HDL	13	51201045	T	G	0.0802	120559
HDL	13	69720694	T	C	0.2851	120559
HDL	13	113927208	T	G	0.1499	120559
HDL	14	20938251	A	C	0.8689	120559
HDL	14	33175822	A	G	0.8150	120559
HDL	14	52436005	A	C	0.8243	120559
HDL	14	52574576	A	C	0.8243	120559
HDL	14	65119839	A	G	0.7930	120559
HDL	14	65914867	A	G	0.8951	120559
HDL	14	67235722	A	G	0.4508	120559
HDL	14	69149372	A	G	0.9114	120559

HDL	14	69273090	A	G	0.7225	120559
HDL	14	74250126	T	C	0.7330	120559
HDL	14	75377352	A	G	0.4462	120559
HDL	14	81635888	T	C	0.4184	120559
HDL	14	88605509	A	C	0.1076	120559
HDL	14	89804276	A	G	0.1298	120559
HDL	14	92498829	A	ATAT	0.3730	120559
HDL	14	98396293	T	C	0.3493	120559
HDL	14	103241799	T	C	0.4823	120559
HDL	14	105258892	T	C	0.2079	120559
HDL	15	23941678	T	C	0.6108	120559
HDL	15	31637569	C	G	0.0967	120559
HDL	15	40744046	T	G	0.6814	120559
HDL	15	41800652	CG	C	0.7028	120559
HDL	15	41888918	A	G	0.9632	120559
HDL	15	43468698	A	G	0.2621	120559
HDL	15	44245931	A	T	0.9567	120559
HDL	15	53001538	T	C	0.8154	120559
HDL	15	57910164	A	G	0.5420	120559
HDL	15	58577163	A	G	0.1222	120559
HDL	15	58577777	A	T	0.5186	120559
HDL	15	58581370	A	T	0.1393	120559
HDL	15	58678720	T	C	0.3776	120559
HDL	15	58680178	T	C	0.5082	120559
HDL	15	58694020	T	G	0.3411	120559
HDL	15	58699937	T	C	0.1421	120559
HDL	15	58714787	T	C	0.9581	120559
HDL	15	58723426	A	G	0.5679	120559
HDL	15	58723479	T	C	0.5680	120559
HDL	15	58724792	A	T	0.6094	120559
HDL	15	58752734	A	C	0.4276	120559
HDL	15	61201424	T	C	0.6637	120559
HDL	15	61948435	A	C	0.2455	120559
HDL	15	63352092	A	G	0.5568	120559
HDL	15	63396867	A	G	0.0131	120559
HDL	15	64167098	A	G	0.9345	120559
HDL	15	67336207	C	G	0.7664	120559
HDL	15	74712937	A	C	0.4007	120559
HDL	15	80539598	T	C	0.7835	120559
HDL	15	81392903	T	G	0.6458	120559
HDL	15	83465789	A	C	0.1868	120559
HDL	15	102068904	T	C	0.5539	120559
HDL	16	11454650	A	C	0.0465	120559
HDL	16	19460842	A	G	0.1594	120559
HDL	16	28917644	T	C	0.1273	120559
HDL	16	29994922	T	C	0.4387	120559
HDL	16	31185882	A	G	0.8025	120559
HDL	16	53805207	A	C	0.1600	120559

HDL	16	53818460	T	G	0.1261	120559
HDL	16	56579961	T	G	0.1311	120559
HDL	16	56801047	T	C	0.0865	120559
HDL	16	56825666	A	G	0.2913	120559
HDL	16	56834254	C	G	0.8748	120559
HDL	16	56929020	C	G	0.0191	120559
HDL	16	56969148	A	G	0.1014	120559
HDL	16	56993886	A	G	0.8296	120559
HDL	16	56995236	A	C	0.5464	120559
HDL	16	57006590	T	C	0.1696	120559
HDL	16	57017662	A	G	0.1087	120559
HDL	16	57017762	C	G	0.2137	120559
HDL	16	57308423	T	G	0.5147	120559
HDL	16	67225501	T	C	0.0131	120559
HDL	16	67911517	T	C	0.9303	120559
HDL	16	67940350	A	G	0.9649	120559
HDL	16	67985706	T	C	0.1572	120559
HDL	16	71856727	T	C	0.0267	120559
HDL	16	81534790	T	C	0.3673	120559
HDL	16	85150163	A	G	0.8919	120559
HDL	16	85951258	A	G	0.7269	120559
HDL	16	88029685	A	G	0.0413	120559
HDL	17	486821	A	C	0.0350	120559
HDL	17	495327	A	G	0.0351	120559
HDL	17	7438834	A	T	0.5769	120559
HDL	17	8054860	A	C	0.1912	120559
HDL	17	26695832	A	G	0.0695	120559
HDL	17	28781792	A	G	0.0746	120559
HDL	17	37830447	A	T	0.4347	120559
HDL	17	38344485	T	C	0.6409	120559
HDL	17	40257163	T	C	0.8585	120559
HDL	17	40781561	T	G	0.2299	120559
HDL	17	42191796	A	G	0.5227	120559
HDL	17	45732605	A	G	0.6782	120559
HDL	17	46957987	A	G	0.2723	120559
HDL	17	48978368	T	C	0.4161	120559
HDL	17	53382829	T	C	0.6946	120559
HDL	17	65892507	C	G	0.6452	120559
HDL	17	66464683	A	G	0.8951	120559
HDL	17	66875294	C	G	0.5587	120559
HDL	17	66901366	T	C	0.5583	120559
HDL	17	68419330	A	G	0.5531	120559
HDL	17	76398404	C	G	0.4322	120559
HDL	17	76769605	A	G	0.6341	120559
HDL	18	19658460	T	C	0.3986	120559
HDL	18	21127910	T	C	0.6227	120559
HDL	18	29797958	T	C	0.7347	120559
HDL	18	40735531	A	G	0.5609	120559

HDL	18	47107152	C	G	0.7382	120559
HDL	18	47118923	T	G	0.5441	120559
HDL	18	47132464	T	C	0.2733	120559
HDL	18	47156188	A	G	0.0699	120559
HDL	18	47167214	T	C	0.1998	120559
HDL	18	47171888	C	G	0.1163	120559
HDL	18	47209125	T	C	0.5772	120559
HDL	18	47209143	T	C	0.1640	120559
HDL	18	47280410	T	C	0.3299	120559
HDL	18	56109859	A	C	0.7388	120559
HDL	18	57739072	T	C	0.1944	120559
HDL	18	57849429	T	C	0.2437	120559
HDL	18	58027496	A	G	0.0359	120559
HDL	19	2671100	T	C	0.5347	120559
HDL	19	3414088	A	G	0.4757	120559
HDL	19	4028783	T	C	0.9110	120559
HDL	19	7220013	C	G	0.6283	120559
HDL	19	7242261	T	C	0.1594	120559
HDL	19	7976698	A	G	0.1614	120559
HDL	19	8431581	A	G	0.0805	120559
HDL	19	8433196	A	C	0.0840	120559
HDL	19	8469738	T	C	0.6484	120559
HDL	19	11269893	T	C	0.1429	120559
HDL	19	11347657	T	C	0.2740	120559
HDL	19	18614935	T	C	0.8271	120559
HDL	19	33891013	A	T	0.8013	120559
HDL	19	33899065	A	G	0.4681	120559
HDL	19	38863464	A	C	0.2356	120559
HDL	19	41333284	T	C	0.6014	120559
HDL	19	41535003	T	C	0.1785	120559
HDL	19	41733145	C	G	0.3721	120559
HDL	19	45242173	A	G	0.1934	120559
HDL	19	45242740	A	G	0.6085	120559
HDL	19	45247627	A	G	0.9126	120559
HDL	19	45329214	T	G	0.8079	120559
HDL	19	45376284	C	G	0.8529	120559
HDL	19	45398264	T	C	0.2986	120559
HDL	19	45411941	T	C	0.9048	120559
HDL	19	45412079	T	C	0.0641	120559
HDL	19	45448465	T	G	0.4775	120559
HDL	19	46385997	A	T	0.4479	120559
HDL	19	47563418	T	C	0.7801	120559
HDL	19	47589895	T	C	0.7742	120559
HDL	19	50121274	A	G	0.4347	120559
HDL	19	52324216	A	G	0.2161	120559
HDL	19	52341757	A	T	0.4320	120559
HDL	19	54815577	T	C	0.1766	120559
HDL	19	56181408	T	C	0.8453	120559

HDL	19	57488423	T	C	0.3491	120559
HDL	20	17596155	A	C	0.5022	120559
HDL	20	19727038	A	G	0.0505	120559
HDL	20	21885619	A	C	0.4834	120559
HDL	20	32542814	A	G	0.8629	120559
HDL	20	33278101	T	C	0.6432	120559
HDL	20	33604042	T	C	0.3591	120559
HDL	20	44538484	T	G	0.6358	120559
HDL	20	44544798	T	C	0.0644	120559
HDL	20	44551855	T	C	0.8834	120559
HDL	20	44554015	T	C	0.9872	120559
HDL	20	44576982	A	G	0.0642	120559
HDL	20	44728661	A	G	0.3754	120559
HDL	20	46290250	T	C	0.8496	120559
HDL	20	46340596	T	C	0.2818	120559
HDL	20	46476143	A	T	0.7050	120559
HDL	20	51263786	A	G	0.5981	120559
HDL	20	55836040	A	G	0.2262	120559
HDL	20	62339059	C	G	0.6731	120559
HDL	20	62711459	T	C	0.7026	120559
HDL	21	27858938	A	AT	0.0161	120559
HDL	21	36343552	T	C	0.4631	120559
HDL	21	43718792	A	G	0.3995	120559
HDL	21	46271452	T	C	0.4405	120559
HDL	21	46294986	C	G	0.3180	120559
HDL	21	46571889	C	G	0.5205	120559
HDL	21	46899279	A	G	0.7849	120559
HDL	22	21925017	A	G	0.6646	120559
HDL	22	21976934	T	C	0.5823	120559
HDL	22	29455687	CCTT	C	0.4671	120559
HDL	22	29905277	A	G	0.9012	120559
HDL	22	30931307	T	C	0.9704	120559
HDL	22	36042986	T	C	0.5760	120559
HDL	22	38572526	C	G	0.7492	120559
HDL	22	38954703	C	G	0.4886	120559
HDL	22	40790565	A	G	0.2806	120559
HDL	22	43150523	T	G	0.8052	120559
HDL	22	44324727	C	G	0.5840	120559
HDL	22	48881562	C	G	0.1133	120559
LDL	1	2819307	A	G	0.7345	118303
LDL	1	10556447	T	G	0.8212	118303
LDL	1	10796866	T	C	0.3601	118303
LDL	1	11889815	A	G	0.1054	118303
LDL	1	16509671	T	C	0.0881	118303
LDL	1	16512586	C	G	0.0881	118303
LDL	1	18808292	A	C	0.8993	118303
LDL	1	23747996	G	GA	0.6333	118303
LDL	1	23795177	T	C	0.2785	118303

LDL	1	25747230	C	G	0.3383	118303
LDL	1	25749269	A	G	0.1245	118303
LDL	1	25769209	G	GA	0.6693	118303
LDL	1	29550991	T	G	0.0623	118303
LDL	1	55487346	C	G	0.8565	118303
LDL	1	55498798	T	C	0.6903	118303
LDL	1	55503448	A	G	0.2819	118303
LDL	1	55518093	A	G	0.0247	118303
LDL	1	55521195	T	C	0.1173	118303
LDL	1	55521313	T	G	0.7510	118303
LDL	1	55526428	T	G	0.9447	118303
LDL	1	55529187	A	G	0.9442	118303
LDL	1	56797981	C	G	0.3925	118303
LDL	1	62920008	A	G	0.8216	118303
LDL	1	62944947	CA	C	0.2224	118303
LDL	1	92968312	T	C	0.0303	118303
LDL	1	93034023	T	G	0.9697	118303
LDL	1	107572997	T	C	0.7070	118303
LDL	1	107617707	A	G	0.7154	118303
LDL	1	109817590	T	G	0.0591	118303
LDL	1	109818530	T	C	0.9404	118303
LDL	1	150255587	T	C	0.2631	118303
LDL	1	150958836	A	G	0.9241	118303
LDL	1	155106227	A	T	0.8719	118303
LDL	1	182970547	A	G	0.3963	118303
LDL	1	183114864	T	TAA	0.4658	118303
LDL	1	198994619	T	C	0.8084	118303
LDL	1	198994696	T	TA	0.8129	118303
LDL	1	220970028	A	G	0.1568	118303
LDL	1	220970593	T	G	0.1252	118303
LDL	1	220975337	T	C	0.0157	118303
LDL	1	220998913	A	G	0.3035	118303
LDL	1	224537655	T	C	0.0144	118303
LDL	1	234726012	A	C	0.7359	118303
LDL	1	234734956	T	C	0.2655	118303
LDL	1	234851216	A	C	0.2949	118303
LDL	1	234853268	A	C	0.2942	118303
LDL	1	235109214	T	C	0.7293	118303
LDL	2	3636478	T	G	0.2977	118303
LDL	2	3642361	T	C	0.7032	118303
LDL	2	8720650	T	G	0.4613	118303
LDL	2	20371380	A	G	0.3399	118303
LDL	2	21232195	A	G	0.0572	118303
LDL	2	21241505	A	G	0.9574	118303
LDL	2	21247065	A	T	0.1205	118303
LDL	2	21252534	A	G	0.1203	118303
LDL	2	21263900	A	G	0.1121	118303
LDL	2	21286057	T	C	0.1005	118303

LDL	2	21533107	A	T	0.0383	118303
LDL	2	25887558	A	G	0.7452	118303
LDL	2	26930411	T	C	0.2199	118303
LDL	2	27730940	T	C	0.5500	118303
LDL	2	43682659	A	T	0.7260	118303
LDL	2	44074000	A	G	0.0105	118303
LDL	2	44075483	T	C	0.9895	118303
LDL	2	44100077	A	G	0.8435	118303
LDL	2	46166321	A	G	0.8210	118303
LDL	2	62955387	A	G	0.7179	118303
LDL	2	63047973	C	G	0.2833	118303
LDL	2	64906800	C	G	0.9087	118303
LDL	2	70471899	A	G	0.3699	118303
LDL	2	113831182	A	T	0.5272	118303
LDL	2	121306440	A	G	0.5007	118303
LDL	2	135451302	T	G	0.0274	118303
LDL	2	135632981	T	C	0.9408	118303
LDL	2	136783169	A	G	0.3888	118303
LDL	2	158434569	T	C	0.2559	118303
LDL	2	158447571	C	G	0.7165	118303
LDL	2	169829810	G	GTA	0.7156	118303
LDL	2	169830155	A	G	0.7163	118303
LDL	2	171540823	A	C	0.7204	118303
LDL	2	203328933	T	C	0.9017	118303
LDL	2	203489457	A	G	0.9541	118303
LDL	2	203527979	T	C	0.2852	118303
LDL	2	203808532	A	G	0.0588	118303
LDL	2	204317553	T	C	0.2520	118303
LDL	2	216300185	T	C	0.9213	118303
LDL	2	234612539	A	C	0.0912	118303
LDL	2	234679384	T	C	0.0861	118303
LDL	2	242370751	T	G	0.6754	118303
LDL	3	12271918	A	AT	0.7247	118303
LDL	3	12327431	T	G	0.9785	118303
LDL	3	12470239	A	C	0.5531	118303
LDL	3	32533010	T	C	0.0542	118303
LDL	3	32538247	T	C	0.0312	118303
LDL	3	69810294	T	G	0.5727	118303
LDL	3	119536429	A	G	0.4569	118303
LDL	3	122285218	CT	C	0.5619	118303
LDL	3	129278182	A	G	0.7551	118303
LDL	3	132163200	T	G	0.1493	118303
LDL	3	132209203	A	G	0.8859	118303
LDL	3	136121600	A	C	0.1354	118303
LDL	3	136258924	C	G	0.9723	118303
LDL	3	142648844	T	G	0.0583	118303
LDL	3	142649110	C	G	0.8719	118303
LDL	3	147033897	C	G	0.7209	118303

LDL	3	149124783	T	C	0.6992	118303
LDL	3	160042459	T	G	0.2254	118303
LDL	3	160171092	T	C	0.1884	118303
LDL	3	170756985	T	G	0.9646	118303
LDL	3	170834559	A	C	0.9240	118303
LDL	4	3443931	A	G	0.4991	118303
LDL	4	7223319	T	G	0.2775	118303
LDL	4	8224276	T	C	0.3800	118303
LDL	4	40418670	T	C	0.3359	118303
LDL	4	69341487	T	C	0.2680	118303
LDL	4	81164723	T	C	0.3855	118303
LDL	4	100239319	T	C	0.7565	118303
LDL	4	154191226	A	G	0.0246	118303
LDL	4	156507678	C	G	0.2040	118303
LDL	4	180470440	A	G	0.0460	118303
LDL	4	187120211	A	C	0.3564	118303
LDL	5	35885982	T	C	0.8227	118303
LDL	5	52193237	A	G	0.9331	118303
LDL	5	55861786	T	C	0.0920	118303
LDL	5	71953629	C	G	0.8724	118303
LDL	5	72014569	A	G	0.1201	118303
LDL	5	74655726	T	C	0.5228	118303
LDL	5	74656539	T	C	0.4764	118303
LDL	5	122848876	A	G	0.3729	118303
LDL	5	122928965	A	G	0.6329	118303
LDL	5	131744574	T	C	0.6499	118303
LDL	5	131804045	T	G	0.4313	118303
LDL	5	139597910	T	C	0.2369	118303
LDL	5	141912841	T	C	0.4845	118303
LDL	5	141913503	A	G	0.5234	118303
LDL	5	156390297	T	C	0.2336	118303
LDL	5	176520243	A	G	0.3915	118303
LDL	5	179471201	T	C	0.2151	118303
LDL	6	16109163	A	C	0.0743	118303
LDL	6	16124560	T	C	0.2090	118303
LDL	6	16126934	A	G	0.7854	118303
LDL	6	30077135	A	C	0.3380	118303
LDL	6	30215377	A	T	0.2755	118303
LDL	6	31240479	T	G	0.8237	118303
LDL	6	31324194	A	G	0.5821	118303
LDL	6	31325323	C	G	0.2838	118303
LDL	6	31352060	T	C	0.7744	118303
LDL	6	31514448	T	C	0.0160	118303
LDL	6	31885930	T	C	0.8875	118303
LDL	6	32602483	T	G	0.2962	118303
LDL	6	32648156	A	G	0.7224	118303
LDL	6	32663954	A	G	0.0836	118303
LDL	6	33143948	C	G	0.2910	118303

LDL	6	34603691	A	G	0.0139	118303
LDL	6	37038432	A	G	0.0876	118303
LDL	6	42919222	T	C	0.6186	118303
LDL	6	52452585	A	G	0.2578	118303
LDL	6	52453220	A	C	0.7380	118303
LDL	6	53509452	A	G	0.8738	118303
LDL	6	100620931	T	C	0.0154	118303
LDL	6	106374015	A	C	0.5522	118303
LDL	6	116312893	A	T	0.8594	118303
LDL	6	116316882	T	G	0.7413	118303
LDL	6	127471533	T	C	0.5222	118303
LDL	6	130389211	A	G	0.5374	118303
LDL	6	135411228	T	C	0.6754	118303
LDL	6	135419631	A	G	0.6736	118303
LDL	6	139317827	G	GC	0.9678	118303
LDL	6	151858598	T	C	0.4558	118303
LDL	6	159958835	T	G	0.2964	118303
LDL	6	160520806	A	G	0.9742	118303
LDL	6	160575985	T	C	0.3113	118303
LDL	6	160820978	A	G	0.5334	118303
LDL	6	160922870	A	G	0.8949	118303
LDL	7	1047615	A	G	0.8083	118303
LDL	7	1074134	T	C	0.7592	118303
LDL	7	6440437	A	G	0.1860	118303
LDL	7	17287269	A	T	0.6172	118303
LDL	7	21449451	T	C	0.0885	118303
LDL	7	21496427	T	C	0.3772	118303
LDL	7	21601659	G	GCTCT	0.7604	118303
LDL	7	21607283	C	G	0.7606	118303
LDL	7	25991826	T	C	0.9742	118303
LDL	7	25992323	A	AAGGCC	0.9739	118303
LDL	7	26015392	A	C	0.5835	118303
LDL	7	28191793	T	C	0.6126	118303
LDL	7	36169203	T	C	0.6836	118303
LDL	7	41750490	T	TAC	0.8035	118303
LDL	7	44579180	C	G	0.3857	118303
LDL	7	44580876	T	G	0.0144	118303
LDL	7	44581986	T	C	0.6092	118303
LDL	7	44606217	C	G	0.9740	118303
LDL	7	46171884	A	G	0.6521	118303
LDL	7	75614777	A	G	0.3158	118303
LDL	7	87074419	T	C	0.7092	118303
LDL	7	97977268	A	G	0.2127	118303
LDL	7	98034664	T	G	0.7859	118303
LDL	7	100216773	T	C	0.0898	118303
LDL	7	100285974	A	C	0.0346	118303
LDL	7	100422481	T	G	0.9830	118303
LDL	7	106801088	T	G	0.0698	118303

LDL	7	107140239	A	G	0.7404	118303
LDL	7	113627084	A	G	0.7078	118303
LDL	7	116486020	A	C	0.6004	118303
LDL	7	130438531	CTTTTTT	C	0.7081	118303
LDL	7	130445428	CT	C	0.2853	118303
LDL	7	134390964	A	T	0.1955	118303
LDL	7	137559799	T	C	0.9819	118303
LDL	7	155026807	T	C	0.5908	118303
LDL	8	6564576	A	G	0.2930	118303
LDL	8	9173209	A	G	0.8906	118303
LDL	8	9183596	A	G	0.0107	118303
LDL	8	9185146	T	C	0.0106	118303
LDL	8	9367743	C	G	0.1763	118303
LDL	8	10508801	C	G	0.2464	118303
LDL	8	18272881	A	G	0.3513	118303
LDL	8	21927460	C	G	0.0128	72815
LDL	8	55437524	A	G	0.7730	118303
LDL	8	55441799	A	G	0.7726	118303
LDL	8	59370320	A	G	0.8502	118303
LDL	8	59393273	A	G	0.1432	118303
LDL	8	59398276	T	C	0.2710	118303
LDL	8	61475573	T	TA	0.5758	118303
LDL	8	74888494	T	C	0.1898	118303
LDL	8	109991668	A	C	0.9280	118303
LDL	8	116658583	T	G	0.2785	118303
LDL	8	126490972	A	T	0.4420	118303
LDL	8	126500031	C	G	0.7923	118303
LDL	8	126506694	A	G	0.2044	118303
LDL	8	141988685	A	G	0.2849	118303
LDL	8	145022657	T	G	0.1614	118303
LDL	8	145031968	T	C	0.1124	118303
LDL	9	2640759	A	G	0.9020	118303
LDL	9	16884586	A	T	0.2500	118303
LDL	9	19217421	T	C	0.0133	118303
LDL	9	19313913	A	G	0.9857	118303
LDL	9	22081850	T	C	0.6780	118303
LDL	9	78729176	A	T	0.4796	118303
LDL	9	78730766	A	G	0.5063	118303
LDL	9	107591272	T	G	0.6856	118303
LDL	9	107595602	A	C	0.3069	118303
LDL	9	107647019	T	C	0.0737	118303
LDL	9	107661742	A	C	0.7471	118303
LDL	9	107664301	T	C	0.2511	118303
LDL	9	131465481	C	G	0.9271	118303
LDL	9	136141870	T	C	0.2598	118303
LDL	9	136146597	T	C	0.2601	118303
LDL	9	139320069	T	G	0.8629	118303
LDL	9	139368953	A	G	0.0555	118303

LDL	10	8100125	A	G	0.0584	118303
LDL	10	17268839	T	C	0.3227	118303
LDL	10	18506426	A	G	0.4022	118303
LDL	10	18720845	T	C	0.0158	118303
LDL	10	32245938	T	TA	0.3481	118303
LDL	10	36066439	A	T	0.5059	118303
LDL	10	45953767	A	G	0.8296	118303
LDL	10	52352431	T	C	0.1053	118303
LDL	10	65005399	T	C	0.3854	118303
LDL	10	65172328	T	C	0.3859	118303
LDL	10	74662593	A	C	0.2106	118303
LDL	10	74692646	T	G	0.7527	118303
LDL	10	94839642	A	G	0.7289	118303
LDL	10	102075479	A	G	0.0479	118303
LDL	10	113910721	A	G	0.3083	118303
LDL	10	113921354	A	G	0.6916	118303
LDL	10	115786233	A	G	0.0098	118303
LDL	10	118397894	A	G	0.7348	118303
LDL	10	124686656	T	C	0.4876	118303
LDL	10	124693587	T	G	0.5123	118303
LDL	11	5677158	T	C	0.9658	118303
LDL	11	5701074	A	C	0.0684	118303
LDL	11	18632984	T	C	0.5494	118303
LDL	11	18639167	T	C	0.4503	118303
LDL	11	19355480	T	C	0.5631	118303
LDL	11	26083007	T	C	0.9564	118303
LDL	11	32153468	A	T	0.2998	118303
LDL	11	46301255	A	G	0.8767	118303
LDL	11	61571382	A	G	0.2976	118303
LDL	11	61588305	A	G	0.6975	118303
LDL	11	65422853	A	C	0.5415	118303
LDL	11	66296569	T	C	0.7370	118303
LDL	11	77976208	T	C	0.3663	118303
LDL	11	103808152	T	C	0.4137	118303
LDL	11	103870640	A	T	0.1162	118303
LDL	11	113577990	A	ATTG	0.9419	118303
LDL	11	116648917	C	G	0.7817	118303
LDL	11	116650638	T	C	0.0790	118303
LDL	11	116660686	A	G	0.7814	118303
LDL	11	118540104	C	G	0.7879	118303
LDL	11	122504717	A	C	0.3015	118303
LDL	11	122518525	A	G	0.8529	118303
LDL	11	126218541	A	G	0.8880	118303
LDL	11	126228000	T	C	0.1201	118303
LDL	12	623129	A	G	0.9604	118303
LDL	12	7625014	T	C	0.9226	118303
LDL	12	9205803	CG	C	0.9281	118303
LDL	12	25407726	A	G	0.9691	118303

LDL	12	26802549	A	G	0.7144	118303
LDL	12	29508642	A	G	0.5498	118303
LDL	12	32145000	A	G	0.1425	118303
LDL	12	40421117	A	G	0.3810	118303
LDL	12	40533337	A	C	0.6111	118303
LDL	12	50650057	T	C	0.0427	118303
LDL	12	51779544	A	AG	0.3964	118303
LDL	12	57650599	A	ATCTC	0.8397	118303
LDL	12	100850750	A	C	0.7679	118303
LDL	12	100942077	A	G	0.2881	118303
LDL	12	109137726	T	C	0.3663	118303
LDL	12	110020894	G	GT	0.8156	118303
LDL	12	111718231	A	C	0.1819	118303
LDL	12	113031543	A	T	0.4049	118303
LDL	12	113039943	T	C	0.3950	118303
LDL	12	113277970	T	G	0.5883	118303
LDL	12	121376416	T	C	0.3128	118303
LDL	12	121416650	A	C	0.5107	118303
LDL	12	124265687	T	C	0.0607	118303
LDL	12	125303254	T	C	0.1301	118303
LDL	12	125307053	A	G	0.1305	118303
LDL	12	125315647	A	G	0.3123	118303
LDL	12	133140853	A	G	0.1667	118303
LDL	13	31016979	T	C	0.8680	118303
LDL	13	32929232	A	G	0.6030	118303
LDL	13	32976656	G	GC	0.4324	118303
LDL	13	33040430	A	G	0.1441	118303
LDL	13	50048401	CA	C	0.2268	118303
LDL	13	50956096	T	C	0.1797	118303
LDL	13	51045297	G	GT	0.8289	118303
LDL	13	111018729	C	G	0.6246	118303
LDL	13	111038325	T	C	0.4369	118303
LDL	13	114551993	T	C	0.6836	118303
LDL	14	24871926	T	C	0.0539	118303
LDL	14	24883887	A	G	0.0426	118303
LDL	14	31725111	A	G	0.2166	118303
LDL	14	35187252	T	C	0.0813	118303
LDL	14	64233717	A	G	0.9259	118303
LDL	14	64236436	T	TA	0.9277	118303
LDL	14	70770867	A	G	0.6864	118303
LDL	14	70847124	T	C	0.9183	118303
LDL	14	70874146	CA	C	0.6954	118303
LDL	14	74250126	T	C	0.7330	118303
LDL	14	75278211	A	G	0.4260	118303
LDL	14	104332759	T	C	0.2809	118303
LDL	15	49317758	A	G	0.7986	118303
LDL	15	49829019	T	C	0.5453	118303
LDL	15	57240500	G	GTAT	0.0192	118303

LDL	15	57512284	A	T	0.9794	118303
LDL	15	58214210	T	C	0.2608	118303
LDL	15	58679807	CAGA	C	0.6213	118303
LDL	15	58692681	T	C	0.2979	118303
LDL	15	58726744	C	G	0.6270	118303
LDL	15	62365465	T	C	0.3834	118303
LDL	15	63328962	A	C	0.9545	118303
LDL	15	65153690	A	G	0.8116	118303
LDL	15	75106719	G	GT	0.8162	118303
LDL	15	75106719	T	G	0.4443	118303
LDL	15	75116167	T	C	0.1609	118303
LDL	15	75180892	T	G	0.7789	118303
LDL	15	75289722	T	C	0.8989	118303
LDL	15	91109611	A	G	0.7641	118303
LDL	16	11631568	T	C	0.4884	118303
LDL	16	11641180	A	G	0.4780	118303
LDL	16	11706100	A	G	0.4027	118303
LDL	16	14381175	C	G	0.5419	118303
LDL	16	31012781	A	G	0.9243	118303
LDL	16	53811788	A	G	0.8758	118303
LDL	16	56988044	T	C	0.2552	118303
LDL	16	56993886	A	G	0.8296	118303
LDL	16	68054788	A	G	0.9088	118303
LDL	16	71639053	A	T	0.6880	118303
LDL	16	72079657	T	C	0.2485	118303
LDL	16	72101525	C	G	0.6950	118303
LDL	16	72217113	T	G	0.7328	118303
LDL	16	72931085	A	G	0.6458	118303
LDL	16	79504057	A	G	0.4905	118303
LDL	16	83980529	C	G	0.2174	118303
LDL	16	88551153	T	C	0.4608	118303
LDL	16	88569287	G	GTC	0.9352	118303
LDL	16	88582871	T	G	0.0206	118303
LDL	17	1942861	T	C	0.9231	118303
LDL	17	7080316	T	C	0.3667	118303
LDL	17	7571080	A	G	0.0358	118303
LDL	17	8216468	T	C	0.2754	118303
LDL	17	26664215	A	G	0.1861	118303
LDL	17	26694861	A	G	0.7451	118303
LDL	17	29466722	A	G	0.5117	118303
LDL	17	40449618	T	C	0.7722	118303
LDL	17	45391804	T	C	0.3607	118303
LDL	17	45662383	T	C	0.3216	118303
LDL	17	46757575	T	G	0.2536	118303
LDL	17	57875554	A	G	0.5125	118303
LDL	17	66401063	T	C	0.2339	118303
LDL	17	67191270	C	G	0.4994	118303
LDL	17	73778609	C	G	0.4805	118303

LDL	17	73878654	A	C	0.6971	118303
LDL	17	76377482	T	G	0.3416	118303
LDL	17	76382791	T	C	0.3940	118303
LDL	17	80530429	T	C	0.2609	118303
LDL	17	81009104	T	C	0.1632	118303
LDL	18	9522606	A	G	0.6380	118303
LDL	18	9526184	T	C	0.3620	118303
LDL	18	21113285	T	C	0.3537	118303
LDL	18	47160953	T	G	0.8844	118303
LDL	19	2802092	T	C	0.3402	118303
LDL	19	2803227	T	C	0.4841	118303
LDL	19	4493708	A	G	0.5702	118303
LDL	19	10669163	C	G	0.0874	118303
LDL	19	10838486	A	G	0.0303	118303
LDL	19	10904689	C	G	0.8846	118303
LDL	19	11133272	A	G	0.7445	118303
LDL	19	11159096	T	G	0.0945	118303
LDL	19	11163601	T	G	0.0997	118303
LDL	19	11185919	T	C	0.9431	118303
LDL	19	11195030	A	G	0.8748	118303
LDL	19	11227480	A	C	0.3820	118303
LDL	19	11229218	A	G	0.8582	118303
LDL	19	11242307	C	G	0.7115	118303
LDL	19	11250139	A	G	0.2360	118303
LDL	19	11335477	A	C	0.2756	118303
LDL	19	11347493	T	C	0.7198	118303
LDL	19	15798015	A	T	0.6844	118303
LDL	19	18340910	A	G	0.8669	118303
LDL	19	19336608	T	C	0.1066	118303
LDL	19	19379549	T	C	0.0749	118303
LDL	19	19388500	A	T	0.9028	118303
LDL	19	19789528	A	G	0.8795	118303
LDL	19	41353107	T	C	0.6942	118303
LDL	19	41353338	A	G	0.7397	118303
LDL	19	44072880	C	G	0.7992	118303
LDL	19	45022560	T	C	0.8464	118303
LDL	19	45140165	A	G	0.4944	118303
LDL	19	45242173	A	G	0.1934	118303
LDL	19	45242740	A	G	0.6085	118303
LDL	19	45247627	A	G	0.9126	118303
LDL	19	45329214	T	G	0.8079	118303
LDL	19	45347911	A	C	0.2088	118303
LDL	19	45373565	A	G	0.1479	118303
LDL	19	45389596	A	G	0.0570	118303
LDL	19	45392254	T	C	0.0949	118303
LDL	19	45401666	A	G	0.7905	118303
LDL	19	45411941	T	C	0.9048	118303
LDL	19	45452845	A	G	0.0712	118303

LDL	19	45490285	A	G	0.0814	118303
LDL	19	45695738	T	C	0.6990	118303
LDL	19	47295358	T	C	0.2306	118303
LDL	19	57011472	CTT	C	0.7541	118303
LDL	20	390310	A	T	0.7746	118303
LDL	20	12958687	A	G	0.3748	118303
LDL	20	12962718	A	G	0.3745	118303
LDL	20	16482169	T	C	0.3499	118303
LDL	20	17804068	A	G	0.1169	118303
LDL	20	17844492	T	G	0.6583	118303
LDL	20	17844684	T	G	0.6629	118303
LDL	20	25206654	A	G	0.0672	118303
LDL	20	25208990	A	G	0.9329	118303
LDL	20	34125271	T	C	0.1356	118303
LDL	20	34147998	A	G	0.8202	118303
LDL	20	39091487	A	G	0.7448	118303
LDL	20	39180436	T	C	0.4295	118303
LDL	20	39672618	A	T	0.8097	118303
LDL	20	39826079	T	C	0.2519	118303
LDL	20	39906988	A	G	0.4715	118303
LDL	20	40234917	A	G	0.0816	118303
LDL	20	44534651	A	G	0.3641	118303
LDL	20	52535400	T	C	0.5548	118303
LDL	20	61341472	A	G	0.2934	118303
LDL	20	62373983	A	G	0.6332	118303
LDL	20	62692060	A	C	0.2911	118303
LDL	20	62909520	A	G	0.0411	118303
LDL	21	37553132	T	G	0.6617	118303
LDL	21	40574305	A	G	0.4341	118303
LDL	21	40709960	T	C	0.5338	118303
LDL	22	19949013	T	C	0.2688	118303
LDL	22	21916272	A	C	0.3138	118303
LDL	22	30378703	T	C	0.0828	118303
LDL	22	30617585	T	C	0.6710	118303
LDL	22	35676138	CT	C	0.5494	118303
LDL	22	35696931	A	G	0.3808	118303
LDL	22	37462936	A	G	0.5124	118303
LDL	22	41268925	T	G	0.5388	118303
LDL	22	41314012	T	C	0.7975	118303
LDL	22	44340904	T	C	0.4211	118303
LDL	22	50067994	T	G	0.5447	118303
LDL	22	50840573	A	G	0.7404	118303
LDL	22	50853626	T	G	0.2586	118303
TC	1	10556447	T	G	0.8212	120561
TC	1	16515805	T	C	0.1900	120561
TC	1	23755513	T	C	0.0706	120561
TC	1	23766233	T	C	0.7269	120561
TC	1	25768937	A	G	0.6693	120561

TC	1	25775733	A	T	0.6694	120561
TC	1	28298951	T	C	0.5077	120561
TC	1	55487346	C	G	0.8565	120561
TC	1	55499156	T	C	0.6904	120561
TC	1	55503448	A	G	0.2819	120561
TC	1	55517301	A	G	0.8875	120561
TC	1	55518160	A	C	0.0258	120561
TC	1	55518467	A	G	0.7023	120561
TC	1	55529187	A	G	0.9442	120561
TC	1	55656075	T	G	0.1877	120561
TC	1	62924448	T	C	0.2233	120561
TC	1	63118196	A	C	0.8258	120561
TC	1	92968312	T	C	0.0303	120561
TC	1	93001430	T	C	0.9697	120561
TC	1	109817192	A	G	0.9395	120561
TC	1	109817590	T	G	0.0591	120561
TC	1	113190807	A	C	0.5035	120561
TC	1	120257576	A	G	0.0697	120561
TC	1	182970547	A	G	0.3963	120561
TC	1	211188937	A	G	0.0967	120561
TC	1	220970028	A	G	0.1568	120561
TC	1	220970593	T	G	0.1252	120561
TC	1	220975337	T	C	0.0157	120561
TC	1	220998913	A	G	0.3035	120561
TC	1	234853268	A	C	0.2942	120561
TC	1	234853406	A	G	0.2949	120561
TC	1	236718620	T	C	0.5262	120561
TC	2	17930195	A	G	0.0798	120561
TC	2	20367973	C	G	0.3631	120561
TC	2	20369701	A	T	0.3634	120561
TC	2	21199426	A	G	0.0401	120561
TC	2	21205502	T	G	0.7281	120561
TC	2	21247065	A	T	0.1205	120561
TC	2	21252534	A	G	0.1203	120561
TC	2	21263900	A	G	0.1121	120561
TC	2	21286057	T	C	0.1005	120561
TC	2	21533107	A	T	0.0383	120561
TC	2	27730940	T	C	0.5500	120561
TC	2	27844601	T	G	0.6697	120561
TC	2	44069772	A	G	0.0108	120561
TC	2	44074000	A	G	0.0105	120561
TC	2	46166321	A	G	0.8210	120561
TC	2	62983213	A	G	0.2814	120561
TC	2	63149557	A	G	0.7184	120561
TC	2	65652156	T	C	0.1342	120561
TC	2	70471899	A	G	0.3699	120561
TC	2	111600519	T	C	0.1498	120561
TC	2	113841030	A	G	0.9507	120561

TC	2	113867288	T	C	0.9508	120561
TC	2	121309488	T	C	0.4583	120561
TC	2	136522941	A	G	0.4115	120561
TC	2	136806774	A	G	0.3487	120561
TC	2	136963494	A	C	0.8234	120561
TC	2	158437683	T	C	0.7155	120561
TC	2	163110536	A	G	0.8309	120561
TC	2	169830155	A	G	0.7163	120561
TC	2	203328933	T	C	0.9017	120561
TC	2	203519264	T	G	0.0452	120561
TC	2	203519783	T	G	0.9548	120561
TC	2	203532304	A	G	0.8948	120561
TC	2	216287093	A	G	0.0712	120561
TC	2	234664586	A	ATC	0.1224	120561
TC	2	234679384	T	C	0.0861	120561
TC	3	12267648	A	G	0.6395	120561
TC	3	12450093	A	G	0.4359	120561
TC	3	12628920	C	G	0.0217	120561
TC	3	32533010	T	C	0.0542	120561
TC	3	32538247	T	C	0.0312	120561
TC	3	64706499	T	C	0.4551	120561
TC	3	122258056	A	G	0.7665	120561
TC	3	132163200	T	G	0.1493	120561
TC	3	132193076	T	TG	0.8852	120561
TC	3	142625778	A	G	0.0814	120561
TC	3	160171092	T	C	0.1884	120561
TC	3	185803532	T	C	0.0250	120561
TC	4	3425030	T	C	0.4724	120561
TC	4	3434885	T	G	0.5065	120561
TC	4	3452345	A	G	0.4378	120561
TC	4	31397618	A	G	0.2849	120561
TC	4	40428010	T	C	0.3971	120561
TC	4	69343287	A	G	0.8880	120561
TC	4	69349893	T	C	0.2676	120561
TC	4	88160140	T	C	0.4143	120561
TC	4	100260545	A	C	0.0450	120561
TC	5	37915720	A	G	0.2324	120561
TC	5	52193237	A	G	0.9331	120561
TC	5	72014569	A	G	0.1201	120561
TC	5	74655726	T	C	0.5228	120561
TC	5	74656539	T	C	0.4764	120561
TC	5	122908468	A	G	0.6325	120561
TC	5	131804045	T	G	0.4313	120561
TC	5	141912841	T	C	0.4845	120561
TC	5	156390297	T	C	0.2336	120561
TC	5	156392248	T	C	0.7665	120561
TC	6	16124560	T	C	0.2090	120561
TC	6	20405539	A	G	0.3803	120561

TC	6	31240479	T	G	0.8237	120561
TC	6	31265490	T	C	0.0836	120561
TC	6	31325323	C	G	0.2838	120561
TC	6	31352060	T	C	0.7744	120561
TC	6	31885930	T	C	0.8875	120561
TC	6	32412435	A	G	0.0834	120561
TC	6	32590735	T	C	0.2964	120561
TC	6	32648156	A	G	0.7224	120561
TC	6	34552797	A	G	0.0149	120561
TC	6	35133074	A	G	0.9742	120561
TC	6	37038432	A	G	0.0876	120561
TC	6	52453220	A	C	0.7380	120561
TC	6	53497222	A	G	0.8758	120561
TC	6	108050222	T	G	0.7412	120561
TC	6	116337503	A	G	0.0353	120561
TC	6	135411228	T	C	0.6754	120561
TC	6	135421067	CT	C	0.6573	120561
TC	6	151858598	T	C	0.4558	120561
TC	6	160506462	A	G	0.2421	120561
TC	6	160575985	T	C	0.3113	120561
TC	6	160820978	A	G	0.5334	120561
TC	7	1067906	T	C	0.8033	120561
TC	7	8998976	A	G	0.1113	120561
TC	7	21496427	T	C	0.3772	120561
TC	7	21582917	T	C	0.0481	120561
TC	7	21598753	C	G	0.2394	120561
TC	7	21607283	C	G	0.7606	120561
TC	7	25991826	T	C	0.9742	120561
TC	7	25992323	A	AAGGCC	0.9739	120561
TC	7	26014642	T	C	0.4165	120561
TC	7	44579180	C	G	0.3857	120561
TC	7	44580876	T	G	0.0144	120561
TC	7	44581986	T	C	0.6092	120561
TC	7	44606217	C	G	0.9740	120561
TC	7	46171884	A	G	0.6521	120561
TC	7	73026151	A	T	0.8958	120561
TC	7	87076587	A	C	0.2910	120561
TC	7	92408370	T	C	0.3281	120561
TC	7	100422481	T	G	0.9830	120561
TC	7	137562668	T	C	0.9818	120561
TC	8	6601492	T	G	0.7048	120561
TC	8	9183358	A	G	0.0108	120561
TC	8	9185146	T	C	0.0106	120561
TC	8	18272881	A	G	0.3513	120561
TC	8	55441799	A	G	0.7726	120561
TC	8	59370320	A	G	0.8502	120561
TC	8	59382311	A	G	0.7277	120561
TC	8	59393273	A	G	0.1432	120561

TC	8	61548494	A	C	0.4133	120561
TC	8	74881674	A	G	0.1764	120561
TC	8	87158577	A	G	0.9750	120561
TC	8	87204944	T	C	0.5445	120561
TC	8	116663898	A	G	0.2821	120561
TC	8	126490972	A	T	0.4420	120561
TC	8	126506694	A	G	0.2044	120561
TC	8	145022657	T	G	0.1614	120561
TC	8	145031968	T	C	0.1124	120561
TC	9	2640759	A	G	0.9020	120561
TC	9	15305378	C	G	0.9148	120561
TC	9	19212560	T	C	0.0131	120561
TC	9	19376255	A	C	0.9839	120561
TC	9	22088094	A	G	0.3632	120561
TC	9	107570714	C	G	0.6636	120561
TC	9	107589134	A	T	0.2879	120561
TC	9	107647019	T	C	0.0737	120561
TC	9	107661742	A	C	0.7471	120561
TC	9	107664301	T	C	0.2511	120561
TC	9	117144795	T	C	0.5457	120561
TC	9	117166246	A	G	0.4751	120561
TC	9	136141870	T	C	0.2598	120561
TC	9	136146597	T	C	0.2601	120561
TC	10	17260290	A	G	0.3199	120561
TC	10	17268839	T	C	0.3227	120561
TC	10	45979232	T	TTAAGTGA	0.9218	120561
TC	10	46007533	T	C	0.0453	120561
TC	10	74662593	A	C	0.2106	120561
TC	10	74665524	T	G	0.0650	120561
TC	10	94839642	A	G	0.7289	120561
TC	10	102027407	T	C	0.0558	120561
TC	10	113913222	T	C	0.3092	120561
TC	10	113933006	A	ATT	0.6911	120561
TC	10	114025182	T	G	0.6800	120561
TC	10	124657809	T	G	0.4805	120561
TC	11	18632984	T	C	0.5494	120561
TC	11	18639167	T	C	0.4503	120561
TC	11	61549025	A	G	0.3037	120561
TC	11	61570783	T	C	0.6982	120561
TC	11	75474195	A	C	0.2209	120561
TC	11	77973980	T	C	0.3793	120561
TC	11	116611827	A	T	0.2236	120561
TC	11	116648917	C	G	0.7817	120561
TC	11	116662579	T	C	0.7014	120561
TC	11	116665079	T	C	0.1573	120561
TC	11	116679155	A	G	0.0610	120561
TC	11	116728630	C	G	0.1200	120561
TC	11	117075566	T	C	0.1193	120561

TC	11	117085270	A	C	0.5458	120561
TC	11	118449370	A	G	0.5759	120561
TC	11	118486067	T	C	0.2271	120561
TC	11	122506970	T	C	0.6933	120561
TC	11	126239143	CT	C	0.2628	120561
TC	11	126244955	A	G	0.3234	120561
TC	12	9098995	G	GAAC	0.6929	120561
TC	12	25408464	A	G	0.9691	120561
TC	12	40606663	T	C	0.6760	120561
TC	12	100850750	A	C	0.7679	120561
TC	12	109137726	T	C	0.3663	120561
TC	12	109965512	C	G	0.6886	120561
TC	12	121416650	A	C	0.5107	120561
TC	12	123757861	A	G	0.6922	120561
TC	12	123867994	A	G	0.7049	120561
TC	12	125315647	A	G	0.3123	120561
TC	12	125353810	A	T	0.1994	120561
TC	13	32953388	T	C	0.4279	120561
TC	13	50198158	A	G	0.2541	120561
TC	13	111018729	C	G	0.6246	120561
TC	13	111025118	A	G	0.5696	120561
TC	13	114527838	A	G	0.3477	120561
TC	14	24871926	T	C	0.0539	120561
TC	14	39983128	T	G	0.8779	120561
TC	14	64235556	T	G	0.0732	120561
TC	14	70817141	T	C	0.0823	120561
TC	14	75278211	A	G	0.4260	120561
TC	15	57442759	A	G	0.9620	120561
TC	15	58581370	A	T	0.1393	120561
TC	15	58678512	T	C	0.2161	120561
TC	15	58680178	T	C	0.5081	120561
TC	15	58699937	T	C	0.1421	120561
TC	15	58723479	T	C	0.5680	120561
TC	15	58726744	C	G	0.6270	120561
TC	16	56987369	T	C	0.8292	120561
TC	16	56990716	A	C	0.1684	120561
TC	16	57000885	A	G	0.0953	120561
TC	16	57006590	T	C	0.1696	120561
TC	16	67997920	T	C	0.8428	120561
TC	16	72105965	T	C	0.7423	120561
TC	16	72108093	A	G	0.3033	120561
TC	16	83979317	T	C	0.2159	120561
TC	17	7080069	A	G	0.6208	120561
TC	17	7080316	T	C	0.3667	120561
TC	17	8219478	A	G	0.2738	120561
TC	17	28632119	CT	C	0.7309	120561
TC	17	29629326	A	G	0.3998	120561
TC	17	45391804	T	C	0.3607	120561

TC	17	45668509	A	G	0.3217	120561
TC	17	57875554	A	G	0.5125	120561
TC	17	67138878	A	C	0.4269	120561
TC	17	73778609	C	G	0.4805	120561
TC	17	76377482	T	G	0.3416	120561
TC	17	76382791	T	C	0.3940	120561
TC	18	9526184	T	C	0.3620	120561
TC	18	47118398	T	C	0.5489	120561
TC	18	47158186	T	C	0.2906	120561
TC	18	47181668	A	T	0.1604	120561
TC	18	47280410	T	C	0.3299	120561
TC	19	2814181	T	C	0.4689	120561
TC	19	10669163	C	G	0.0874	120561
TC	19	10904689	C	G	0.8846	120561
TC	19	11133272	A	G	0.7445	120561
TC	19	11159096	T	G	0.0945	120561
TC	19	11196886	T	C	0.9600	120561
TC	19	11224265	A	G	0.3877	120561
TC	19	11227602	T	C	0.1391	120561
TC	19	11250139	A	G	0.2360	120561
TC	19	11343795	A	G	0.7227	120561
TC	19	11347493	T	C	0.7198	120561
TC	19	18304700	A	G	0.8953	120561
TC	19	19336608	T	C	0.1066	120561
TC	19	19379549	T	C	0.0749	120561
TC	19	19393890	A	AG	0.9004	120561
TC	19	19686071	T	TG	0.0243	120561
TC	19	19789528	A	G	0.8795	120561
TC	19	45140165	A	G	0.4944	120561
TC	19	45242173	A	G	0.1934	120561
TC	19	45247627	A	G	0.9126	120561
TC	19	45329214	T	G	0.8079	120561
TC	19	45373565	A	G	0.1479	120561
TC	19	45389596	A	G	0.0570	120561
TC	19	45401666	A	G	0.7905	120561
TC	19	45408628	T	C	0.9340	120561
TC	19	45411941	T	C	0.9048	120561
TC	19	45415640	A	G	0.0816	120561
TC	19	45422946	A	G	0.8906	120561
TC	19	50012574	A	G	0.1611	120561
TC	20	16482169	T	C	0.3499	120561
TC	20	17844684	T	G	0.6629	120561
TC	20	31392777	T	C	0.8924	120561
TC	20	34125271	T	C	0.1356	120561
TC	20	34152782	T	C	0.1361	120561
TC	20	39091487	A	G	0.7448	120561
TC	20	39154095	A	G	0.4465	120561
TC	20	39672618	A	T	0.8097	120561

TC	20	39830122	A	G	0.1922	120561
TC	21	40574305	A	G	0.4341	120561
TC	22	35711098	A	G	0.4695	120561
TC	22	44324727	C	G	0.5840	120561
TG	1	10796866	T	C	0.3601	120377
TG	1	11838451	T	C	0.0893	120377
TG	1	23787021	A	C	0.7271	120377
TG	1	26865971	T	C	0.9280	120377
TG	1	39363294	A	G	0.6901	120377
TG	1	40048009	A	G	0.1079	120377
TG	1	40435999	A	G	0.5319	120377
TG	1	47049747	A	C	0.9703	120377
TG	1	51351846	A	G	0.1077	120377
TG	1	51353720	C	G	0.8923	120377
TG	1	54877103	T	C	0.6264	120377
TG	1	62904575	T	G	0.7764	120377
TG	1	63145439	A	G	0.1745	120377
TG	1	63191777	T	C	0.0190	120377
TG	1	77461735	A	G	0.6901	120377
TG	1	93565123	A	G	0.6982	120377
TG	1	93854517	T	C	0.3864	120377
TG	1	98378786	A	C	0.0496	120377
TG	1	98478981	T	C	0.9604	120377
TG	1	118143517	A	G	0.1543	120377
TG	1	154093825	A	G	0.4463	120377
TG	1	154251259	A	T	0.5812	120377
TG	1	172349246	A	C	0.4719	120377
TG	1	172353311	T	C	0.9051	120377
TG	1	178508930	A	G	0.3983	120377
TG	1	201355653	T	G	0.0242	120377
TG	1	203511492	A	G	0.5003	120377
TG	1	205070573	A	G	0.8944	120377
TG	1	210592392	T	G	0.6543	120377
TG	1	214147889	T	C	0.4157	120377
TG	1	214629616	A	G	0.4801	120377
TG	1	216074957	A	C	0.0513	120377
TG	1	219637671	A	T	0.6209	120377
TG	1	219734960	A	C	0.6790	120377
TG	1	219762070	T	G	0.9001	120377
TG	1	220998913	A	G	0.3035	120377
TG	1	228056868	T	C	0.2971	120377
TG	1	230294916	T	C	0.1607	120377
TG	1	230301811	T	G	0.7429	120377
TG	1	230410811	T	C	0.0619	120377
TG	1	237565709	A	T	0.2982	120377
TG	1	237852083	T	C	0.7403	120377
TG	2	3639909	C	G	0.7055	120377
TG	2	20374286	A	G	0.6571	120377

TG	2	21215645	T	C	0.0785	120377
TG	2	21231524	A	G	0.7408	120377
TG	2	21232804	A	G	0.0572	120377
TG	2	21278773	T	G	0.1226	120377
TG	2	21286057	T	C	0.1005	120377
TG	2	21510295	T	C	0.3344	120377
TG	2	25592918	T	C	0.3360	120377
TG	2	25928446	A	G	0.1266	120377
TG	2	26914787	A	G	0.2537	120377
TG	2	27730940	T	C	0.5500	120377
TG	2	27757343	A	G	0.6660	120377
TG	2	27950837	A	G	0.3043	120377
TG	2	28610627	T	C	0.2808	120377
TG	2	37075013	A	T	0.5987	120377
TG	2	43490619	A	T	0.7833	120377
TG	2	50716016	T	C	0.4526	120377
TG	2	58933591	T	C	0.6180	120377
TG	2	59313974	T	C	0.6577	120377
TG	2	65276049	T	C	0.6586	120377
TG	2	65284623	T	C	0.6714	120377
TG	2	66665146	T	C	0.7121	120377
TG	2	85788270	A	C	0.3778	120377
TG	2	111927379	A	G	0.5382	120377
TG	2	121309231	C	G	0.7046	120377
TG	2	145818432	A	G	0.2656	120377
TG	2	146347459	A	G	0.3880	120377
TG	2	161340112	A	T	0.0859	120377
TG	2	165528876	T	C	0.1003	120377
TG	2	169514699	T	C	0.9795	46363
TG	2	171629063	C	G	0.6939	120377
TG	2	202122995	A	G	0.2521	120377
TG	2	202185132	A	G	0.2737	120377
TG	2	203431804	A	G	0.9287	120377
TG	2	219184275	T	C	0.6164	120377
TG	2	227099180	T	C	0.9436	120377
TG	2	227163642	T	C	0.9336	120377
TG	2	227229344	C	G	0.2958	120377
TG	2	230128204	T	G	0.1384	120377
TG	2	235209795	A	G	0.9185	120377
TG	2	239896861	A	G	0.4314	120377
TG	2	242395674	A	G	0.6761	120377
TG	3	4763301	A	G	0.1215	120377
TG	3	12139092	T	C	0.0331	120377
TG	3	12173655	T	C	0.8537	120377
TG	3	12473045	A	G	0.5449	120377
TG	3	15681940	A	C	0.1807	120377
TG	3	24520283	A	G	0.4955	120377
TG	3	36979042	A	G	0.5767	120377

TG	3	37025661	T	C	0.9082	120377
TG	3	47265877	A	AG	0.7230	120377
TG	3	47484953	C	G	0.5009	120377
TG	3	52344680	A	G	0.9649	120377
TG	3	52532118	A	G	0.0230	120377
TG	3	69879670	T	G	0.0280	120377
TG	3	135798658	T	C	0.1245	120377
TG	3	136005792	C	G	0.1328	120377
TG	3	142652559	T	G	0.0264	120377
TG	3	142657205	T	G	0.5427	120377
TG	3	142669092	A	AT	0.5849	120377
TG	3	155547274	T	C	0.2275	120377
TG	3	156795414	T	G	0.5315	120377
TG	3	156915089	A	G	0.8469	120377
TG	3	185548663	A	G	0.1578	120377
TG	3	187628006	T	G	0.9483	120377
TG	3	188421362	A	G	0.2718	120377
TG	3	196187608	A	G	0.2629	120377
TG	4	951947	T	C	0.8437	120377
TG	4	965720	A	G	0.5811	120377
TG	4	3443931	A	G	0.4991	120377
TG	4	3446883	A	G	0.5380	120377
TG	4	4990298	A	G	0.4916	120377
TG	4	15096523	A	G	0.9796	120377
TG	4	18034463	A	G	0.9045	120377
TG	4	26331471	A	G	0.2524	120377
TG	4	39646631	T	C	0.1168	120377
TG	4	55535336	A	G	0.4296	120377
TG	4	69366825	A	ATCTC	0.1403	120377
TG	4	69367872	A	G	0.1403	120377
TG	4	77373079	T	C	0.1024	120377
TG	4	80564508	A	G	0.3189	120377
TG	4	86931091	T	C	0.3678	120377
TG	4	87862396	C	G	0.6041	120377
TG	4	87985166	T	C	0.3578	120377
TG	4	88168178	T	C	0.3464	120377
TG	4	88230501	A	C	0.9243	120377
TG	4	89713121	T	C	0.7047	120377
TG	4	89741269	A	G	0.6748	120377
TG	4	100014805	A	G	0.1939	120377
TG	4	104174843	A	C	0.2500	120377
TG	4	124743259	A	G	0.8652	120377
TG	4	143317027	A	G	0.2712	120377
TG	4	157682598	T	C	0.7525	120377
TG	4	164245854	A	G	0.6672	120377
TG	5	10467702	A	G	0.7027	120377
TG	5	53283630	A	T	0.8815	120377
TG	5	53308654	CT	C	0.5204	120377

TG	5	55783832	T	C	0.3194	120377
TG	5	55806751	A	G	0.4868	120377
TG	5	55808475	T	C	0.5139	120377
TG	5	55854153	T	C	0.0459	120377
TG	5	55857025	G	GT	0.9083	120377
TG	5	55990342	A	G	0.3199	120377
TG	5	57610069	C	G	0.7530	120377
TG	5	67714246	A	G	0.5422	120377
TG	5	77631634	A	G	0.9231	120377
TG	5	78531337	C	G	0.5240	120377
TG	5	90255685	A	G	0.4377	120377
TG	5	103995368	A	G	0.3374	120377
TG	5	112490629	A	C	0.9215	120377
TG	5	118713456	A	AT	0.8613	120377
TG	5	131008194	T	C	0.1873	120377
TG	5	132442190	T	G	0.8804	120377
TG	5	132444301	T	C	0.8805	120377
TG	5	140933792	T	C	0.0790	120377
TG	5	144501147	T	C	0.1264	120377
TG	5	149345975	A	G	0.4605	120377
TG	5	153800513	A	G	0.2442	120377
TG	5	156394441	A	G	0.7665	120377
TG	5	156398169	C	G	0.7656	120377
TG	5	157999022	A	G	0.2491	120377
TG	5	173324971	T	G	0.0671	120377
TG	6	7249460	A	G	0.0298	120377
TG	6	18747705	T	G	0.7884	120377
TG	6	20486798	T	C	0.0633	120377
TG	6	20506815	A	G	0.8440	120377
TG	6	26148326	A	G	0.8290	120377
TG	6	26319486	T	C	0.0440	120377
TG	6	29323655	A	G	0.0289	120377
TG	6	29807393	C	G	0.3825	120377
TG	6	29834951	T	C	0.2592	120377
TG	6	31241182	T	C	0.8399	120377
TG	6	31265539	A	G	0.5207	120377
TG	6	31325323	C	G	0.2838	120377
TG	6	31475056	A	G	0.0224	120377
TG	6	31840021	A	T	0.8873	120377
TG	6	32210799	A	G	0.5437	120377
TG	6	32331002	T	C	0.8575	120377
TG	6	32412435	A	G	0.0834	120377
TG	6	32587213	T	C	0.2318	120377
TG	6	32600585	A	G	0.1711	120377
TG	6	32669373	T	C	0.0521	120377
TG	6	34188892	A	C	0.9735	120377
TG	6	36638175	A	G	0.7075	120377
TG	6	36645696	A	G	0.4270	120377

TG	6	43757896	A	C	0.5590	120377
TG	6	43758873	A	G	0.7669	120377
TG	6	43804571	T	G	0.0968	120377
TG	6	52630269	A	C	0.8392	120377
TG	6	86662267	T	C	0.7103	120377
TG	6	96848669	A	G	0.9195	120377
TG	6	106378009	T	G	0.3762	120377
TG	6	107437166	T	C	0.1721	120377
TG	6	109189021	C	G	0.1993	120377
TG	6	111834954	T	C	0.0109	120377
TG	6	127184987	A	T	0.5419	120377
TG	6	127414801	T	C	0.5610	120377
TG	6	127435106	A	T	0.4816	120377
TG	6	130354855	T	C	0.1578	120377
TG	6	133827354	A	G	0.2344	120377
TG	6	139835399	C	G	0.7347	120377
TG	6	139837431	T	G	0.7354	120377
TG	6	140611419	A	G	0.5305	120377
TG	6	153473232	T	C	0.3264	120377
TG	6	160562481	A	G	0.1080	120377
TG	6	160820978	A	G	0.5334	120377
TG	6	161013013	T	C	0.9752	120377
TG	6	164092291	A	C	0.1020	120377
TG	6	164133001	T	C	0.1053	120377
TG	6	167548547	A	G	0.9386	120377
TG	7	1010801	C	G	0.9682	120377
TG	7	12269417	C	G	0.3478	120377
TG	7	15905325	T	C	0.4262	120377
TG	7	17287269	A	T	0.6172	120377
TG	7	17920613	T	C	0.5267	120377
TG	7	21496427	T	C	0.3772	120377
TG	7	21582917	T	C	0.0481	120377
TG	7	21607283	C	G	0.7606	120377
TG	7	25935747	A	C	0.0483	120377
TG	7	25965759	A	G	0.4288	120377
TG	7	25991826	T	C	0.9742	120377
TG	7	25997536	A	G	0.4083	120377
TG	7	26394297	A	G	0.3340	120377
TG	7	28256240	A	G	0.0156	120377
TG	7	29319249	A	G	0.0275	120377
TG	7	32262377	A	C	0.6898	120377
TG	7	44197583	T	G	0.3029	120377
TG	7	44205906	A	C	0.5082	120377
TG	7	44231778	T	C	0.4220	120377
TG	7	44267538	T	C	0.1033	120377
TG	7	72767451	T	C	0.1390	120377
TG	7	73016181	T	C	0.8990	120377
TG	7	73025975	A	G	0.8968	120377

TG	7	76036364	A	G	0.5308	120377
TG	7	93083588	T	G	0.5994	120377
TG	7	106632113	A	G	0.5717	120377
TG	7	112722196	A	G	0.9727	120377
TG	7	116358044	A	G	0.4083	120377
TG	7	116486020	A	C	0.6004	120377
TG	7	117705257	T	C	0.0949	120377
TG	7	130432538	A	T	0.7080	120377
TG	7	130442603	CT	C	0.4197	120377
TG	7	130583442	T	C	0.7390	120377
TG	7	150210213	G	GGCT	0.1678	120377
TG	7	150296609	A	G	0.8180	120377
TG	8	1373720	A	G	0.6054	120377
TG	8	8316637	T	C	0.8634	120377
TG	8	8363683	A	G	0.3605	120377
TG	8	9181395	A	G	0.0106	120377
TG	8	9184691	T	C	0.0105	120377
TG	8	9389241	T	C	0.6356	120377
TG	8	10193772	C	G	0.9309	120377
TG	8	10491684	C	G	0.0731	120377
TG	8	10644101	C	G	0.0141	120377
TG	8	10671260	A	G	0.7089	120377
TG	8	11045161	A	G	0.0751	120377
TG	8	11521079	A	T	0.0669	120377
TG	8	11650475	T	C	0.6628	120377
TG	8	11836318	A	C	0.4170	120377
TG	8	16279180	T	G	0.3037	120377
TG	8	17884606	T	C	0.7570	120377
TG	8	18272377	T	C	0.3516	120377
TG	8	19662937	T	C	0.0611	120377
TG	8	19810787	A	C	0.1324	120377
TG	8	19817476	T	G	0.9032	120377
TG	8	19824563	T	C	0.8750	120377
TG	8	19824667	T	C	0.2097	120377
TG	8	19928013	A	T	0.2505	120377
TG	8	19942181	T	G	0.2015	120377
TG	8	22457804	T	C	0.2064	120377
TG	8	25464670	T	G	0.2935	120377
TG	8	38329650	T	C	0.2899	120377
TG	8	59388565	T	C	0.1488	120377
TG	8	64608119	A	T	0.9881	120377
TG	8	72395582	A	AAGCCCT	0.1186	120377
TG	8	72459889	A	G	0.8508	120377
TG	8	72469742	A	G	0.2488	120377
TG	8	116670347	T	C	0.2822	120377
TG	8	118191475	T	C	0.4115	120377
TG	8	126409614	A	G	0.7066	120377
TG	8	126490972	A	T	0.4420	120377

TG	8	126506694	A	G	0.2044	120377
TG	8	126630967	T	C	0.2779	120377
TG	8	126694377	A	T	0.9334	120377
TG	8	144306970	A	G	0.1935	120377
TG	9	1033773	C	G	0.5699	120377
TG	9	13676484	A	G	0.7499	120377
TG	9	15305378	C	G	0.9148	120377
TG	9	16887366	A	T	0.2498	120377
TG	9	16905824	T	C	0.2508	120377
TG	9	22134253	A	G	0.4381	120377
TG	9	24957550	A	G	0.1262	120377
TG	9	33787871	T	C	0.2263	120377
TG	9	86583076	T	C	0.2910	120377
TG	9	92183196	T	C	0.0251	120377
TG	9	92202495	T	C	0.7340	120377
TG	9	95283887	A	G	0.4274	120377
TG	9	107664301	T	C	0.2511	120377
TG	9	107665978	C	G	0.8056	120377
TG	9	112241136	T	C	0.7569	120377
TG	9	123358262	A	G	0.5375	120377
TG	9	134854280	T	C	0.3786	120377
TG	9	136141870	T	C	0.2598	120377
TG	9	139386138	A	G	0.0532	120377
TG	10	5245780	T	G	0.0866	120377
TG	10	5267191	A	G	0.9135	120377
TG	10	17268839	T	C	0.3227	120377
TG	10	56639940	A	G	0.6505	120377
TG	10	65027610	A	T	0.6167	120377
TG	10	65290254	A	AT	0.6228	120377
TG	10	70346740	T	C	0.4255	120377
TG	10	71411206	A	G	0.5160	120377
TG	10	74703849	T	C	0.7636	120377
TG	10	74714177	T	G	0.2658	120377
TG	10	77217080	A	G	0.7749	120377
TG	10	81096071	T	C	0.7404	120377
TG	10	94259180	A	G	0.0282	120377
TG	10	94367867	A	T	0.2367	120377
TG	10	94839642	A	G	0.7289	120377
TG	10	94839724	T	G	0.7280	120377
TG	10	99772404	A	G	0.2492	120377
TG	10	113933886	A	G	0.3086	120377
TG	10	113937941	T	G	0.6914	120377
TG	10	113978850	A	G	0.6984	120377
TG	10	115798895	A	G	0.2117	120377
TG	10	120242187	T	C	0.3745	120377
TG	10	122999550	A	G	0.5385	120377
TG	10	134459388	A	G	0.6666	120377
TG	11	13361524	T	C	0.3147	120377

TG	11	18301915	A	G	0.9660	120377
TG	11	27675712	T	C	0.7656	120377
TG	11	30504660	A	G	0.9230	120377
TG	11	36446712	T	C	0.4448	120377
TG	11	47259668	T	C	0.7262	120377
TG	11	48693639	T	C	0.0799	120377
TG	11	49329521	T	C	0.7390	120377
TG	11	61579463	A	G	0.6984	120377
TG	11	61596633	T	C	0.3051	120377
TG	11	62200846	T	C	0.4269	120377
TG	11	62378221	A	G	0.9451	120377
TG	11	63877163	T	C	0.3102	120377
TG	11	65391317	A	G	0.5071	120377
TG	11	65473798	A	C	0.2685	120377
TG	11	66079786	A	G	0.6382	120377
TG	11	68597805	A	T	0.1822	120377
TG	11	68603346	T	C	0.6518	120377
TG	11	78105879	T	C	0.3843	120377
TG	11	111760738	T	G	0.7705	120377
TG	11	113296619	T	C	0.4232	120377
TG	11	116596309	A	C	0.8897	120377
TG	11	116647607	T	C	0.7823	120377
TG	11	116661392	A	C	0.0788	120377
TG	11	116662579	T	C	0.7014	120377
TG	11	116665079	T	C	0.1573	120377
TG	11	116667337	T	C	0.4069	120377
TG	11	116667545	T	C	0.0532	120377
TG	11	116830261	T	C	0.8316	120377
TG	11	117085270	A	C	0.5458	120377
TG	12	11791628	C	G	0.0916	120377
TG	12	20474706	A	G	0.8548	120377
TG	12	21331549	T	C	0.8566	120377
TG	12	21343886	A	G	0.8360	120377
TG	12	22765864	A	G	0.9417	120377
TG	12	26453283	A	G	0.2515	120377
TG	12	26457190	A	G	0.2515	120377
TG	12	29437861	A	G	0.5812	120377
TG	12	37896288	A	T	0.9661	120377
TG	12	46200396	T	G	0.1173	120377
TG	12	54429385	A	G	0.3894	120377
TG	12	56863770	C	G	0.1054	120377
TG	12	57766392	C	G	0.1119	120377
TG	12	57844049	T	C	0.0945	120377
TG	12	62838230	T	C	0.0164	120377
TG	12	66351826	T	C	0.1478	120377
TG	12	67645247	A	T	0.2822	120377
TG	12	69732105	T	C	0.5836	120377
TG	12	70369918	A	G	0.7042	120377

TG	12	103537266	A	T	0.8266	120377
TG	12	107174646	A	C	0.6642	120377
TG	12	107228676	C	G	0.3361	120377
TG	12	109699616	T	C	0.2659	120377
TG	12	123206340	T	C	0.4481	120377
TG	12	123895906	C	G	0.7042	120377
TG	12	124423817	T	C	0.8894	120377
TG	12	124464836	T	G	0.1086	120377
TG	12	124503803	CAA	C	0.5542	120377
TG	12	125303254	T	C	0.1301	120377
TG	12	125312425	C	G	0.6888	120377
TG	13	25820101	T	C	0.3112	120377
TG	13	29145323	A	C	0.7417	120377
TG	13	31007805	A	C	0.6751	120377
TG	13	31012904	T	C	0.7959	120377
TG	13	51007525	CT	C	0.2515	120377
TG	13	51221618	A	C	0.9201	120377
TG	13	74188014	A	T	0.2970	120377
TG	13	95253131	A	C	0.3359	120377
TG	13	95258944	T	C	0.6750	120377
TG	13	99245866	T	C	0.7196	120377
TG	13	111018729	C	G	0.6246	120377
TG	13	112245313	A	G	0.4607	120377
TG	13	114524944	A	G	0.6690	120377
TG	13	114544024	T	C	0.7709	120377
TG	14	23733114	A	T	0.2035	120377
TG	14	24545375	T	C	0.5296	120377
TG	14	52425506	A	G	0.7181	120377
TG	14	52480621	A	G	0.7667	120377
TG	14	64235556	T	G	0.0732	120377
TG	14	64236191	T	C	0.9256	120377
TG	14	65119839	A	G	0.7930	120377
TG	14	70327334	T	C	0.0134	120377
TG	14	71541026	T	C	0.6314	120377
TG	14	77503443	A	G	0.1263	120377
TG	14	104290813	A	G	0.7282	120377
TG	15	31637666	T	C	0.9037	120377
TG	15	39464167	A	G	0.4985	120377
TG	15	40397191	C	G	0.8545	120377
TG	15	40751555	A	C	0.8384	120377
TG	15	42125234	T	C	0.1382	120377
TG	15	44245931	A	T	0.9567	120377
TG	15	51898058	T	C	0.5926	120377
TG	15	57154952	A	T	0.8547	120377
TG	15	57278634	G	GTTGA	0.0192	120377
TG	15	58680178	T	C	0.5082	120377
TG	15	58683366	A	G	0.5169	120377
TG	15	58699937	T	C	0.1421	120377

TG	15	58723479	T	C	0.5680	120377
TG	15	58731153	C	G	0.3846	120377
TG	15	60902512	T	C	0.0863	120377
TG	15	61947694	T	C	0.2329	120377
TG	15	61960302	A	T	0.7711	120377
TG	15	63374127	T	C	0.9724	120377
TG	15	66872114	T	C	0.3155	120377
TG	15	66878900	A	G	0.3235	120377
TG	15	67418391	A	G	0.4538	120377
TG	15	70207077	A	G	0.6506	120377
TG	15	72103427	T	C	0.2921	120377
TG	15	73088869	A	T	0.7686	120377
TG	15	73618238	T	C	0.5839	120377
TG	15	99196112	A	G	0.4718	120377
TG	15	101890913	A	G	0.5698	120377
TG	15	102067841	T	C	0.5706	120377
TG	16	962154	T	C	0.6043	120377
TG	16	2450570	T	C	0.8664	120377
TG	16	4488191	A	G	0.7404	120377
TG	16	4676852	A	C	0.1015	120377
TG	16	11792700	A	C	0.7322	120377
TG	16	13814473	T	C	0.4894	120377
TG	16	15129970	A	G	0.5683	120377
TG	16	29994922	T	C	0.4387	120377
TG	16	31054040	C	G	0.9251	120377
TG	16	31136066	A	AAAAG	0.0802	120377
TG	16	53826028	T	C	0.8411	120377
TG	16	56989590	T	C	0.1691	120377
TG	16	56993886	A	G	0.8296	120377
TG	16	57006590	T	C	0.1696	120377
TG	16	58834402	A	C	0.9724	120377
TG	16	67925435	A	G	0.0351	120377
TG	16	67928042	A	G	0.0352	120377
TG	16	69378445	A	G	0.8654	120377
TG	16	69552785	C	G	0.9236	120377
TG	16	69622762	A	G	0.1243	120377
TG	16	72097827	T	C	0.3029	120377
TG	16	72212044	A	G	0.2633	120377
TG	16	79754440	A	T	0.7108	120377
TG	16	81534790	T	C	0.3673	120377
TG	16	81614892	A	G	0.2334	120377
TG	16	85150163	A	G	0.8919	120377
TG	16	85198965	T	G	0.8798	120377
TG	16	85707367	C	G	0.4926	120377
TG	16	86422112	A	G	0.2289	120377
TG	16	88004092	A	G	0.9551	120377
TG	17	599924	A	G	0.9624	120377
TG	17	1618363	T	C	0.8451	120377

TG	17	7459299	T	G	0.7001	120377
TG	17	7537792	T	C	0.5592	120377
TG	17	17409560	T	C	0.2740	120377
TG	17	17453067	T	C	0.2904	120377
TG	17	26694861	A	G	0.7451	120377
TG	17	37807698	A	C	0.5544	120377
TG	17	37832366	A	G	0.4355	120377
TG	17	40698075	A	G	0.7709	120377
TG	17	43194413	T	C	0.1579	120377
TG	17	45732605	A	G	0.6782	120377
TG	17	46197755	A	G	0.1982	120377
TG	17	46957987	A	G	0.2723	120377
TG	17	47407071	A	C	0.6123	120377
TG	17	48624523	A	C	0.9113	120377
TG	17	57906288	C	G	0.6274	120377
TG	17	58024324	A	G	0.5224	120377
TG	17	65854807	A	G	0.6033	120377
TG	17	65989961	C	G	0.3612	120377
TG	17	68453345	A	G	0.3240	120377
TG	17	68465984	CT	C	0.3240	120377
TG	17	73309269	A	G	0.9459	120377
TG	17	74273165	A	G	0.6099	120377
TG	17	74281391	T	C	0.4052	120377
TG	17	76391454	T	G	0.5689	120377
TG	17	76398404	C	G	0.4322	120377
TG	17	76439361	A	G	0.0409	120377
TG	18	268992	A	C	0.2298	120377
TG	18	289209	T	C	0.0375	120377
TG	18	2846812	A	T	0.8987	120377
TG	18	19910184	C	G	0.5932	120377
TG	18	19911690	A	G	0.4047	120377
TG	18	21116998	A	G	0.3541	120377
TG	18	21161134	T	C	0.9370	120377
TG	18	57861663	T	C	0.7716	120377
TG	18	60845884	T	C	0.5363	120377
TG	19	4139440	T	C	0.0361	120377
TG	19	4139849	T	C	0.0363	120377
TG	19	4965064	A	G	0.0708	120377
TG	19	7189375	T	C	0.4138	120377
TG	19	7220596	T	G	0.3766	120377
TG	19	7223961	A	ATTT	0.3636	120377
TG	19	11291782	A	C	0.9827	120377
TG	19	11350488	T	C	0.2749	120377
TG	19	19379549	T	C	0.0749	120377
TG	19	19455750	A	G	0.0182	120377
TG	19	19789528	A	G	0.8795	120377
TG	19	19925148	T	C	0.3942	120377
TG	19	33751349	A	G	0.0384	120377

TG	19	33889593	A	G	0.5482	120377
TG	19	33899065	A	G	0.4681	120377
TG	19	35559787	T	C	0.9739	120377
TG	19	41742691	G	GA	0.3830	120377
TG	19	45373565	A	G	0.1479	120377
TG	19	45413224	T	TG	0.4557	120377
TG	19	45416178	T	G	0.1664	120377
TG	19	45448465	T	G	0.4775	120377
TG	19	46796768	A	G	0.3254	120377
TG	19	46802122	A	T	0.4553	120377
TG	19	46878989	A	G	0.0384	120377
TG	19	50016748	T	C	0.0295	120377
TG	19	50016759	T	C	0.1476	120377
TG	19	56102362	A	G	0.1074	120377
TG	19	57488423	T	C	0.3491	120377
TG	20	31091206	T	G	0.0177	120377
TG	20	34126827	T	C	0.8644	120377
TG	20	38556466	A	G	0.2123	120377
TG	20	39154095	A	G	0.4465	120377
TG	20	39780932	A	G	0.3313	120377
TG	20	44544798	T	C	0.0644	120377
TG	20	44545048	T	C	0.3497	120377
TG	20	44554015	T	C	0.9872	120377
TG	20	44576982	A	G	0.0642	120377
TG	20	51012164	A	C	0.9441	120377
TG	20	51235613	A	G	0.4195	120377
TG	20	56098733	T	C	0.2583	120377
TG	20	56113783	A	G	0.4518	120377
TG	20	62695931	A	G	0.7062	120377
TG	20	62711459	T	C	0.7026	120377
TG	21	40465534	A	G	0.2145	120377
TG	21	40553845	T	C	0.8578	120377
TG	21	46582100	T	C	0.5212	120377
TG	22	24335977	A	G	0.2356	120377
TG	22	28807625	T	C	0.2213	120377
TG	22	33258288	T	C	0.9294	120377
TG	22	36042986	T	C	0.5760	120377
TG	22	37469192	A	G	0.4987	120377
TG	22	38575498	CT	C	0.7492	120377
TG	22	38600542	T	C	0.6500	120377
TG	22	38879010	A	T	0.3959	120377
TG	22	48182991	A	G	0.1406	120377
AST	1	14320502	T	G	0.1908	109230
AST	1	110408241	T	C	0.1878	109230
AST	1	183802259	T	C	0.7128	109230
AST	2	169870295	A	C	0.3324	109230
AST	2	233503989	A	G	0.2304	109230
AST	4	88176030	A	C	0.3233	109230

AST	4	146821410	A	C	0.4190	109230
AST	5	39424628	A	C	0.6098	109230
AST	5	72395189	A	G	0.3852	109230
AST	6	32191339	T	C	0.1489	109230
AST	6	33076268	T	C	0.3849	109230
AST	6	62697746	A	C	0.1543	109230
AST	6	135419631	A	G	0.6736	109230
AST	7	50258479	A	T	0.6603	109230
AST	9	33117965	T	C	0.6965	109230
AST	9	117134191	T	C	0.5044	109230
AST	9	134016121	T	C	0.2312	109230
AST	10	18222342	T	C	0.7061	109230
AST	10	18510448	A	G	0.3857	109230
AST	10	100240374	T	C	0.0706	109230
AST	10	101132282	A	C	0.6440	109230
AST	10	101520941	A	G	0.0188	109230
AST	11	93913692	T	C	0.8038	109230
AST	12	111718231	A	C	0.1819	109230
AST	14	94642962	T	C	0.4053	109230
AST	15	25145127	A	G	0.0180	109230
AST	16	58764855	A	C	0.4393	109230
AST	19	50012574	A	G	0.1611	109230
AST	22	44328730	A	G	0.4155	109230
ALT	1	18135882	A	G	0.7012	109068
ALT	1	27661037	T	C	0.7768	109068
ALT	1	167156500	T	C	0.7367	109068
ALT	2	37149498	T	C	0.3578	109068
ALT	2	169870295	A	C	0.3324	109068
ALT	2	233520254	A	T	0.7399	109068
ALT	3	99088011	A	C	0.1061	109068
ALT	3	149212268	A	G	0.0232	109068
ALT	4	77416627	A	G	0.4487	109068
ALT	4	88213808	A	G	0.6975	109068
ALT	4	146821410	A	C	0.4190	109068
ALT	5	56087899	T	C	0.1438	109068
ALT	6	33860843	T	G	0.0481	109068
ALT	7	28179258	A	C	0.3792	109068
ALT	7	55937441	A	T	0.4045	109068
ALT	7	87079406	A	T	0.2907	109068
ALT	8	126482077	A	G	0.4306	109068
ALT	9	6665010	T	C	0.1155	109068
ALT	9	27869510	A	G	0.2959	109068
ALT	9	85414981	A	G	0.5310	109068
ALT	9	108655096	T	G	0.4913	109068
ALT	10	79680514	A	C	0.4993	109068
ALT	10	98460581	A	G	0.2816	109068
ALT	10	101861435	A	G	0.0604	109068
ALT	10	113949664	T	C	0.3071	109068

ALT	11	93913692	T	C	0.8038	109068
ALT	12	111718231	A	C	0.1819	109068
ALT	13	23763282	T	C	0.6159	109068
ALT	13	40807483	C	G	0.4493	109068
ALT	13	81475442	T	C	0.1902	109068
ALT	16	15728274	T	C	0.9753	109068
ALT	18	4247231	T	C	0.2352	109068
ALT	19	10347084	T	C	0.5250	109068
ALT	21	30126482	A	G	0.7512	109068
ALT	22	44325996	A	G	0.4150	109068
GGT	1	16505320	A	G	0.1854	102729
GGT	1	23376541	T	G	0.5061	102729
GGT	1	89146234	A	C	0.5367	102729
GGT	1	111684276	A	G	0.8186	102729
GGT	1	154086512	A	G	0.4450	102729
GGT	1	155126948	T	C	0.1290	102729
GGT	1	178513411	T	C	0.4941	102729
GGT	1	200261014	A	C	0.4452	102729
GGT	2	27730940	T	C	0.5499	102729
GGT	2	66073552	A	G	0.1047	102729
GGT	2	169834370	A	G	0.3280	102729
GGT	2	185462041	T	C	0.4667	102729
GGT	2	192057406	A	C	0.3484	102729
GGT	2	192117238	T	C	0.3607	102729
GGT	2	233520254	A	T	0.7398	102729
GGT	3	4909440	T	C	0.4740	102729
GGT	3	149186927	A	G	0.9698	102729
GGT	3	149210443	T	C	0.5039	102729
GGT	4	3446883	A	G	0.5380	102729
GGT	4	146821410	A	C	0.4190	102729
GGT	5	31020521	A	G	0.7432	102729
GGT	5	31024853	T	C	0.8584	102729
GGT	5	52193125	A	G	0.9330	102729
GGT	5	156743083	C	G	0.2638	102729
GGT	6	53903152	A	G	0.5069	102729
GGT	6	53924697	T	C	0.4871	102729
GGT	6	66414828	A	G	0.2565	102729
GGT	6	104521604	T	C	0.9856	102729
GGT	6	121902869	A	G	0.6940	102729
GGT	7	26022414	A	C	0.2983	102729
GGT	7	73026378	T	C	0.1037	102729
GGT	7	97826232	A	G	0.4466	102729
GGT	7	116438511	T	G	0.8696	102729
GGT	9	131466489	T	G	0.0728	102729
GGT	10	79680434	A	T	0.7913	102729
GGT	10	79688208	A	G	0.2333	102729
GGT	11	15941099	T	C	0.5832	102729
GGT	11	34764919	A	C	0.7455	102729

GGT	11	62199817	T	C	0.7064	102729
GGT	12	53274674	T	G	0.2090	102729
GGT	12	111515020	C	G	0.1163	102729
GGT	12	111718231	A	C	0.1818	102729
GGT	12	121420260	A	G	0.4755	102729
GGT	12	121424861	A	G	0.4733	102729
GGT	13	19591944	T	G	0.8291	102729
GGT	14	102994549	A	C	0.0245	102729
GGT	14	103550388	C	G	0.5519	102729
GGT	14	103572815	A	G	0.7648	102729
GGT	15	39932972	A	G	0.6187	102729
GGT	15	60878030	A	G	0.9124	102729
GGT	15	60883281	A	C	0.9123	102729
GGT	15	73986264	T	G	0.4472	102729
GGT	16	80497601	A	C	0.3901	102729
GGT	17	70098161	C	G	0.8403	102729
GGT	17	73175294	T	C	0.3783	102729
GGT	18	56084054	T	C	0.1757	102729
GGT	19	47414986	A	G	0.7180	102729
GGT	21	30555154	A	G	0.1988	102729
GGT	22	18439958	T	C	0.0245	102729
GGT	22	24256894	T	C	0.5407	102729
GGT	22	24295286	T	C	0.7631	102729
GGT	22	24999104	C	G	0.6552	102729
GGT	22	35292510	A	C	0.2666	102729
GGT	22	38971950	T	C	0.4981	102729
FPG	1	26865971	T	C	0.9280	109942
FPG	1	214159256	T	C	0.6337	109942
FPG	1	214181766	A	C	0.3014	109942
FPG	2	27152874	T	C	0.1908	109942
FPG	2	27741105	A	G	0.5419	109942
FPG	2	27741237	T	C	0.5382	109942
FPG	2	27995781	A	C	0.5857	109942
FPG	2	45188353	T	C	0.3733	109942
FPG	2	77437279	T	G	0.6954	109942
FPG	2	169750483	T	C	0.8184	109942
FPG	2	169763148	T	C	0.0290	109942
FPG	2	169764491	T	C	0.9798	109942
FPG	2	169767148	T	C	0.6474	109942
FPG	2	169774071	A	C	0.9690	109942
FPG	2	173592663	C	G	0.5546	109942
FPG	3	30346968	A	G	0.8774	109942
FPG	3	62790623	A	G	0.0222	109942
FPG	3	124918754	T	C	0.8936	109942
FPG	3	170735099	A	G	0.1933	109942
FPG	3	185507299	A	T	0.6860	109942
FPG	4	121801790	A	G	0.3830	109942
FPG	5	55774613	A	G	0.7197	109942

FPG	5	95542726	A	C	0.2991	109942
FPG	5	129943319	T	C	0.6456	109942
FPG	5	164056594	T	C	0.9782	109942
FPG	6	7240577	A	T	0.7920	109942
FPG	6	20673880	T	C	0.4594	109942
FPG	6	20685486	T	C	0.5334	109942
FPG	6	31745518	A	G	0.9432	109942
FPG	6	32383050	A	G	0.0198	109942
FPG	6	39033595	A	G	0.2089	109942
FPG	7	15064309	T	G	0.6745	109942
FPG	7	15065467	T	C	0.6737	109942
FPG	7	44216137	A	G	0.4923	109942
FPG	7	44231886	T	C	0.8123	109942
FPG	7	44245853	A	T	0.7835	109942
FPG	7	50751090	T	C	0.1016	109942
FPG	8	9183596	A	G	0.0107	109942
FPG	8	40484239	T	C	0.2783	109942
FPG	8	77823083	C	G	0.0252	109942
FPG	8	118185733	A	G	0.5806	109942
FPG	8	118549376	T	C	0.9587	109942
FPG	9	622523	T	G	0.1700	109942
FPG	9	4285119	C	G	0.5862	109942
FPG	9	4289050	A	C	0.4060	109942
FPG	9	22134094	T	C	0.5622	109942
FPG	10	65091110	T	C	0.9847	109942
FPG	10	94367867	A	T	0.2367	109942
FPG	10	113042093	T	G	0.0937	109942
FPG	10	114732882	T	C	0.2127	109942
FPG	10	114756041	A	T	0.9718	109942
FPG	11	2735534	C	G	0.2317	109942
FPG	11	2857233	A	G	0.3929	109942
FPG	11	8243798	A	G	0.8977	109942
FPG	11	45873091	A	C	0.7941	109942
FPG	11	46300722	A	G	0.0782	109942
FPG	11	47336320	A	T	0.9789	109942
FPG	11	61571478	T	C	0.6982	109942
FPG	11	92708710	C	G	0.5696	109942
FPG	11	126481440	T	C	0.5744	109942
FPG	12	62392548	A	G	0.1065	109942
FPG	12	72343287	T	C	0.0892	109942
FPG	12	111718231	A	C	0.1819	109942
FPG	12	121938185	C	G	0.7652	109942
FPG	13	28487599	A	G	0.5341	109942
FPG	13	28499962	A	G	0.4586	109942
FPG	13	28510712	T	C	0.5765	109942
FPG	13	33554302	A	G	0.8333	109942
FPG	14	90034972	A	G	0.1202	109942
FPG	14	105623261	A	G	0.2466	109942

FPG	15	62396942	A	G	0.4611	109942
FPG	15	62433962	A	G	0.6514	109942
FPG	15	99258710	A	T	0.4759	109942
FPG	16	53797908	C	G	0.8578	109942
FPG	17	40261545	T	C	0.1137	109942
FPG	17	43185492	C	G	0.2109	109942
FPG	17	47037598	T	G	0.8047	109942
FPG	20	22557099	A	G	0.1483	109942
FPG	20	22581268	T	C	0.8717	109942
FPG	21	41939569	A	C	0.9713	109942
HBA1C	1	25529038	A	G	0.2745	51385
HBA1C	1	150940625	T	G	0.9223	51385
HBA1C	1	156233189	T	C	0.2318	51385
HBA1C	1	156318141	A	C	0.2332	51385
HBA1C	1	158585415	T	C	0.4166	51385
HBA1C	2	24021231	T	C	0.9755	51385
HBA1C	2	45192000	A	G	0.5774	51385
HBA1C	2	48414735	T	C	0.9346	51385
HBA1C	2	169763148	T	C	0.0290	51385
HBA1C	2	169767148	T	C	0.6473	51385
HBA1C	2	169792171	T	C	0.3734	51385
HBA1C	2	175292364	T	C	0.7629	51385
HBA1C	2	189377509	A	G	0.0710	51385
HBA1C	3	12267648	A	G	0.6395	51385
HBA1C	3	49382925	A	G	0.0678	51385
HBA1C	3	158904859	A	G	0.8443	51385
HBA1C	3	170724883	T	C	0.7805	51385
HBA1C	3	170732599	A	G	0.1923	51385
HBA1C	3	171795540	A	G	0.7532	51385
HBA1C	3	185518921	A	G	0.2939	51385
HBA1C	4	144659795	A	G	0.2548	51385
HBA1C	5	8305570	A	G	0.9163	51385
HBA1C	6	20661250	C	G	0.4782	51385
HBA1C	6	20679709	A	G	0.4587	51385
HBA1C	6	26107463	A	G	0.0601	51385
HBA1C	6	109562035	A	G	0.7445	51385
HBA1C	6	135419018	T	C	0.6748	51385
HBA1C	6	139840693	A	C	0.7612	51385
HBA1C	7	44229068	T	C	0.1827	51385
HBA1C	7	127841626	A	C	0.1081	51385
HBA1C	8	41549194	T	C	0.9493	51385
HBA1C	8	41630405	A	G	0.5209	51385
HBA1C	8	41630447	A	G	0.5241	51385
HBA1C	8	42383084	A	G	0.4981	51385
HBA1C	8	118185733	A	G	0.5807	51385
HBA1C	9	22132076	A	G	0.5884	51385
HBA1C	9	22137685	T	G	0.6424	51385
HBA1C	9	110536932	A	G	0.8167	51385

HBA1C	9	139328722	T	G	0.2065	51385
HBA1C	10	12253597	A	G	0.4543	51385
HBA1C	10	71091013	A	G	0.8323	51385
HBA1C	10	71099913	T	C	0.7115	51385
HBA1C	10	114752503	T	C	0.9740	51385
HBA1C	10	114758349	T	C	0.0280	51385
HBA1C	11	199256	A	G	0.5430	51385
HBA1C	11	205198	A	C	0.4416	51385
HBA1C	11	2858440	A	G	0.3907	51385
HBA1C	11	61604814	A	C	0.3055	51385
HBA1C	11	72432985	A	G	0.0614	51385
HBA1C	11	92673828	T	C	0.4211	51385
HBA1C	11	100456604	T	C	0.2192	51385
HBA1C	12	7075882	T	C	0.0387	51385
HBA1C	12	48409054	A	C	0.1111	51385
HBA1C	13	28487599	A	G	0.5341	51385
HBA1C	13	113351662	A	G	0.9713	51385
HBA1C	15	77799657	A	G	0.5711	51385
HBA1C	16	293562	T	C	0.4217	51385
HBA1C	16	53803574	A	T	0.1247	51385
HBA1C	16	68750190	A	G	0.1730	51385
HBA1C	16	88844932	T	C	0.3628	51385
HBA1C	17	27183104	A	G	0.5760	51385
HBA1C	17	42241929	T	G	0.9231	51385
HBA1C	17	76124865	C	G	0.8450	51385
HBA1C	17	76127431	C	G	0.4439	51385
HBA1C	17	76137477	T	G	0.8219	51385
HBA1C	17	80685426	A	G	0.4774	51385
HBA1C	19	17232499	T	C	0.3752	51385
HBA1C	19	17246737	A	T	0.6262	51385
HBA1C	19	17256523	A	G	0.1086	51385
HBA1C	19	46159986	T	C	0.4699	51385
HBA1C	22	37462936	A	G	0.5124	51385
T2D	1	6676040	A	G	0.7926	211793
T2D	1	11317932	T	C	0.1069	211793
T2D	1	16050470	A	C	0.9470	211793
T2D	1	20707153	A	G	0.6257	211793
T2D	1	22068326	A	G	0.2426	211793
T2D	1	26396065	A	G	0.8795	211793
T2D	1	26466831	A	G	0.8959	211793
T2D	1	26803430	T	C	0.8615	211793
T2D	1	26868639	A	G	0.2358	211793
T2D	1	29024956	A	G	0.2268	211793
T2D	1	36789546	A	G	0.9402	211793
T2D	1	39797055	A	G	0.7201	211793
T2D	1	39913351	A	G	0.8350	211793
T2D	1	51103268	T	C	0.2140	211039
T2D	1	51209148	T	C	0.9040	211039

T2D	1	67010654	A	C	0.1607	211793
T2D	1	72751552	A	G	0.9891	211214
T2D	1	72838529	A	G	0.0186	211793
T2D	1	88416590	T	C	0.8601	211039
T2D	1	112292303	T	C	0.6355	211793
T2D	1	120471224	A	G	0.9736	211793
T2D	1	154294260	T	C	0.8329	211793
T2D	1	154324384	C	G	0.3989	211793
T2D	1	155262613	C	G	0.2648	211793
T2D	1	155269776	A	G	0.2640	211793
T2D	1	172368310	A	G	0.1066	211793
T2D	1	177889025	A	C	0.7868	211793
T2D	1	179248952	A	G	0.5566	211793
T2D	1	183004334	A	G	0.5355	211793
T2D	1	201849926	A	G	0.9057	211793
T2D	1	204490470	A	G	0.6096	211039
T2D	1	205044339	A	G	0.1416	211793
T2D	1	205114873	C	G	0.0865	211793
T2D	1	205789455	A	T	0.6953	211793
T2D	1	206593900	C	G	0.7868	211793
T2D	1	206618799	A	C	0.7703	211793
T2D	1	207652176	A	G	0.7474	211039
T2D	1	214159256	T	C	0.6162	211793
T2D	1	214272242	A	C	0.9786	208438
T2D	1	219660535	A	G	0.5875	211793
T2D	1	219748818	C	G	0.6715	211793
T2D	1	233340154	A	G	0.7336	211793
T2D	1	235542023	A	G	0.3824	211793
T2D	2	646674	T	C	0.0929	211793
T2D	2	25635771	A	G	0.9819	211694
T2D	2	27741237	T	C	0.5381	211793
T2D	2	31068019	C	G	0.6733	211793
T2D	2	43227262	T	C	0.7650	211039
T2D	2	43732823	T	C	0.9921	211118
T2D	2	53397048	C	G	0.8183	211793
T2D	2	57287411	A	G	0.7300	211793
T2D	2	58956557	T	G	0.8262	211793
T2D	2	60557705	A	G	0.4895	211793
T2D	2	60585806	T	C	0.3221	211793
T2D	2	60586707	C	G	0.3344	211793
T2D	2	65279805	T	C	0.6756	211793
T2D	2	65287896	A	G	0.6762	211793
T2D	2	65655012	C	G	0.8580	211793
T2D	2	67622243	A	G	0.4068	211793
T2D	2	67878328	T	C	0.1951	211793
T2D	2	86707504	T	C	0.0440	211793
T2D	2	105165674	A	G	0.9092	211793
T2D	2	111950541	T	C	0.4409	211793

T2D	2	121318166	C	G	0.8518	211793
T2D	2	121378852	T	C	0.8883	211039
T2D	2	145261174	A	C	0.5905	211793
T2D	2	145704420	A	G	0.7944	211793
T2D	2	146352541	T	G	0.3879	211793
T2D	2	147833920	A	G	0.6668	211793
T2D	2	147920213	T	C	0.6540	211793
T2D	2	149433085	A	G	0.5463	211793
T2D	2	149455385	A	G	0.5468	211793
T2D	2	152167830	T	C	0.4248	211793
T2D	2	161136656	A	G	0.1696	211793
T2D	2	161171454	T	C	0.1566	211793
T2D	2	165513091	T	C	0.9099	211793
T2D	2	172796774	A	T	0.8863	211793
T2D	2	181618654	A	G	0.7654	211793
T2D	2	196952010	T	C	0.6129	211793
T2D	2	208870017	A	G	0.5939	211793
T2D	2	212274937	A	G	0.7427	211793
T2D	2	213687103	T	C	0.8992	211793
T2D	2	213829721	T	C	0.9622	211793
T2D	2	227020653	A	G	0.8169	211793
T2D	2	227101309	T	C	0.0763	211793
T2D	2	227101411	A	G	0.0763	211793
T2D	2	228971884	T	C	0.5052	211793
T2D	2	228973660	A	G	0.3056	211793
T2D	2	234303281	C	G	0.5955	211793
T2D	3	12393125	C	G	0.9611	200914
T2D	3	12489342	T	G	0.1090	200160
T2D	3	15741389	T	C	0.6046	211793
T2D	3	23077761	T	C	0.6523	211793
T2D	3	23454790	A	G	0.8210	211793
T2D	3	23455582	T	C	0.8212	211793
T2D	3	23510044	A	G	0.8586	211039
T2D	3	31176875	A	G	0.9028	211039
T2D	3	35667761	A	G	0.8542	211793
T2D	3	36870230	A	G	0.4074	211793
T2D	3	46925539	T	C	0.2956	211793
T2D	3	49860854	T	C	0.7358	211793
T2D	3	49995518	T	C	0.8364	211793
T2D	3	53125429	A	T	0.8807	211793
T2D	3	54575859	A	C	0.9801	210460
T2D	3	55313400	A	C	0.2594	211793
T2D	3	63948566	T	C	0.6273	211793
T2D	3	64701146	A	G	0.1992	211793
T2D	3	86756871	T	G	0.7233	211793
T2D	3	114913508	A	G	0.3056	211793
T2D	3	121965199	T	G	0.5140	211793
T2D	3	124926637	T	C	0.2393	211793

T2D	3	129293256	T	C	0.5857	207126
T2D	3	131644937	A	G	0.7285	211793
T2D	3	138053187	A	G	0.2795	211793
T2D	3	149196752	C	G	0.8956	211793
T2D	3	152053250	T	C	0.5210	211793
T2D	3	152086533	A	G	0.5300	211793
T2D	3	152382352	A	C	0.2777	211793
T2D	3	160153305	A	G	0.7506	106187
T2D	3	170724883	T	C	0.7804	211793
T2D	3	173107443	T	C	0.4040	211039
T2D	3	173119768	A	G	0.3102	211039
T2D	3	183738460	A	C	0.1619	211793
T2D	3	184877626	A	G	0.6245	211793
T2D	3	185510613	T	G	0.6893	211793
T2D	3	185520948	A	G	0.6895	211793
T2D	3	187698333	T	C	0.8187	211793
T2D	3	195831237	T	C	0.3087	211793
T2D	4	1010077	T	C	0.7883	205793
T2D	4	1236502	C	G	0.6807	206372
T2D	4	1246038	A	T	0.6840	211039
T2D	4	6289986	T	G	0.0328	207027
T2D	4	17792869	A	C	0.5001	211793
T2D	4	17922866	A	G	0.9063	211793
T2D	4	20265535	T	C	0.1831	211793
T2D	4	48880627	C	G	0.4300	211793
T2D	4	49067323	A	G	0.6011	211793
T2D	4	52798624	T	C	0.4011	211793
T2D	4	52818664	A	G	0.4000	211793
T2D	4	71753515	T	C	0.2059	211793
T2D	4	76496817	A	G	0.1545	211793
T2D	4	76535086	A	G	0.1539	211793
T2D	4	77528821	A	C	0.2707	211793
T2D	4	83578271	A	G	0.3867	211793
T2D	4	85339618	T	C	0.9765	211214
T2D	4	85384069	A	G	0.0127	211039
T2D	4	89740894	C	G	0.2881	211793
T2D	4	91288559	A	C	0.8167	211793
T2D	4	95091911	A	G	0.2374	211793
T2D	4	96114385	A	G	0.3443	211793
T2D	4	103895317	T	G	0.5019	211793
T2D	4	106048291	T	C	0.8096	211793
T2D	4	123833154	T	C	0.0540	211694
T2D	4	130786346	A	G	0.6865	211793
T2D	4	137083193	A	C	0.9274	211793
T2D	4	140877659	A	G	0.7289	211793
T2D	4	140906390	T	C	0.7261	211793
T2D	4	156697784	T	C	0.7530	211039
T2D	4	164532801	T	C	0.8089	211793

T2D	4	168660101	A	G	0.2690	211039
T2D	4	185708807	T	C	0.9872	208514
T2D	4	186580062	A	G	0.7596	208438
T2D	5	14768092	C	G	0.5560	211793
T2D	5	44682589	A	G	0.5045	211039
T2D	5	46204748	A	G	0.4180	201062
T2D	5	51791225	T	C	0.6505	211793
T2D	5	52100489	A	G	0.8600	211793
T2D	5	52110814	A	G	0.5262	211793
T2D	5	54986775	T	C	0.2684	211793
T2D	5	55806751	A	G	0.5075	211793
T2D	5	55810305	A	T	0.5077	211793
T2D	5	55848972	C	G	0.4741	211793
T2D	5	56104308	A	G	0.4332	211793
T2D	5	58132702	T	C	0.4725	211793
T2D	5	74574984	A	G	0.4279	211793
T2D	5	75003678	T	C	0.4361	211793
T2D	5	77532068	T	C	0.8426	211793
T2D	5	86577352	A	G	0.6168	211793
T2D	5	95850250	A	C	0.5871	211793
T2D	5	101620174	T	C	0.3123	211793
T2D	5	122650885	T	C	0.9099	211793
T2D	5	134240235	T	C	0.3633	211793
T2D	5	151324600	T	G	0.7069	211793
T2D	5	157928196	T	C	0.7209	211793
T2D	5	170683134	A	G	0.8769	211793
T2D	6	7269548	A	G	0.9782	211793
T2D	6	7281654	A	G	0.5747	211793
T2D	6	7290437	A	G	0.5747	211793
T2D	6	19718157	T	C	0.2974	211793
T2D	6	20679709	A	G	0.5053	211793
T2D	6	20682622	T	C	0.5837	211793
T2D	6	29816421	A	G	0.7139	211793
T2D	6	31026236	T	G	0.7959	211793
T2D	6	31129616	A	G	0.3444	211793
T2D	6	31204694	C	G	0.8319	211793
T2D	6	31264461	A	C	0.0417	211214
T2D	6	31347451	A	G	0.5084	211793
T2D	6	32138545	A	C	0.0896	211793
T2D	6	32573415	A	G	0.8976	210460
T2D	6	32627714	T	G	0.4093	207605
T2D	6	32654714	T	C	0.9083	211793
T2D	6	32668031	A	C	0.5218	211793
T2D	6	33542523	T	C	0.7560	211793
T2D	6	33552707	A	C	0.7554	211793
T2D	6	34214670	A	G	0.8925	211793
T2D	6	38106844	T	C	0.7604	211793
T2D	6	39046644	A	C	0.7118	211793

T2D	6	39281456	A	C	0.8817	211793
T2D	6	40409243	T	C	0.5976	211793
T2D	6	40576652	T	C	0.6519	211793
T2D	6	41012117	A	C	0.7481	211793
T2D	6	41012405	A	G	0.7479	211793
T2D	6	43760327	C	G	0.4488	211793
T2D	6	44875762	A	T	0.6918	211793
T2D	6	50788778	A	C	0.7298	211793
T2D	6	64163807	T	C	0.4999	211793
T2D	6	71289189	A	G	0.9088	211793
T2D	6	111738793	T	C	0.9942	205781
T2D	6	117850608	T	C	0.8651	211793
T2D	6	117996631	T	C	0.7859	211793
T2D	6	126052359	A	G	0.3464	211793
T2D	6	126792095	A	G	0.5257	208547
T2D	6	126964510	A	G	0.5312	209301
T2D	6	127401978	A	T	0.9491	211793
T2D	6	127416930	A	G	0.8866	211793
T2D	6	131954797	T	G	0.6436	211793
T2D	6	137293227	T	C	0.4508	211793
T2D	6	137302159	T	G	0.4546	211793
T2D	6	138864489	T	C	0.3560	211793
T2D	6	139205386	T	C	0.4011	211793
T2D	6	139835329	A	T	0.7307	211793
T2D	6	143056556	T	C	0.7997	211793
T2D	6	143073041	T	C	0.1582	211793
T2D	6	153427706	C	G	0.1848	211793
T2D	6	153428102	A	G	0.1840	211793
T2D	6	160770312	A	G	0.6907	211793
T2D	7	7249747	A	G	0.7962	211793
T2D	7	13894276	A	G	0.4687	211793
T2D	7	13900731	A	G	0.5841	211793
T2D	7	14923907	A	T	0.5944	211039
T2D	7	15064190	C	G	0.3189	211793
T2D	7	15064896	T	G	0.3196	211793
T2D	7	15203359	A	C	0.2936	211793
T2D	7	15926228	T	C	0.1114	211793
T2D	7	23884697	A	G	0.6933	211793
T2D	7	28196222	A	G	0.7726	211793
T2D	7	44174857	T	G	0.3730	211793
T2D	7	44245363	A	G	0.6742	211214
T2D	7	48839003	A	G	0.9415	211793
T2D	7	50365413	T	C	0.9235	211039
T2D	7	50577968	T	C	0.7410	211793
T2D	7	50581972	A	G	0.9109	211793
T2D	7	50887174	T	C	0.2267	211793
T2D	7	55802063	T	C	0.3571	211793
T2D	7	69189726	A	G	0.2868	211793

T2D	7	69696905	A	G	0.6638	211793
T2D	7	74076493	A	G	0.8854	208722
T2D	7	77047102	A	G	0.8278	211793
T2D	7	89803634	T	C	0.7772	211793
T2D	7	93107093	A	C	0.3230	211793
T2D	7	100312724	A	G	0.0445	206372
T2D	7	102336979	T	C	0.8152	207802
T2D	7	102800137	T	C	0.0716	208547
T2D	7	127164958	A	G	0.2276	211793
T2D	7	127237312	T	C	0.3344	211793
T2D	7	127862802	A	G	0.1009	211793
T2D	7	130027037	A	C	0.5745	211793
T2D	7	130457914	A	G	0.5170	211793
T2D	7	130466854	A	G	0.2890	211793
T2D	7	131574608	A	C	0.0382	209301
T2D	7	140522073	A	G	0.9189	211039
T2D	7	142607301	A	G	0.9754	210940
T2D	7	149238823	T	C	0.8293	211793
T2D	7	157024510	A	G	0.5053	211793
T2D	7	157027753	T	C	0.0719	211793
T2D	7	157032924	T	G	0.5067	211793
T2D	8	9974584	A	G	0.9842	211694
T2D	8	10633159	A	C	0.6527	211793
T2D	8	10808687	A	T	0.9008	206547
T2D	8	12811580	T	G	0.4144	211793
T2D	8	14124809	A	C	0.8031	211793
T2D	8	14148990	A	C	0.7845	211793
T2D	8	19843171	T	C	0.8833	211793
T2D	8	25871721	T	G	0.9715	211793
T2D	8	25872634	A	G	0.9709	211793
T2D	8	30854033	A	G	0.0569	211793
T2D	8	30863938	T	C	0.0600	211793
T2D	8	34502571	A	C	0.5245	211793
T2D	8	36858483	A	G	0.6394	211793
T2D	8	36859186	T	C	0.6355	211793
T2D	8	37397803	T	C	0.5526	211793
T2D	8	38343012	T	C	0.3278	211793
T2D	8	41506152	C	G	0.5475	211793
T2D	8	41519462	A	G	0.1791	211793
T2D	8	41552046	T	C	0.9987	195638
T2D	8	68613390	T	C	0.8393	211793
T2D	8	73503743	A	C	0.7576	211793
T2D	8	74568099	A	G	0.9261	211793
T2D	8	75214398	A	G	0.9761	211694
T2D	8	95685147	A	C	0.7542	211793
T2D	8	95961626	T	C	0.3003	211793
T2D	8	95967372	A	G	0.2989	211793
T2D	8	97138738	A	G	0.7197	211793

T2D	8	110123183	C	G	0.4618	211793
T2D	8	116565365	T	C	0.2153	211793
T2D	8	132879795	T	C	0.7477	211793
T2D	8	145536056	A	G	0.5286	207126
T2D	8	145551199	C	G	0.5289	207126
T2D	8	145879883	T	C	0.7212	211039
T2D	8	146003567	A	G	0.2797	211793
T2D	9	3249708	C	G	0.8462	211793
T2D	9	4291928	A	C	0.6373	211039
T2D	9	4293150	A	C	0.5577	211793
T2D	9	14141703	A	G	0.7537	211793
T2D	9	19067833	A	G	0.7876	211793
T2D	9	19080352	A	G	0.7733	211793
T2D	9	20241069	T	C	0.1192	211793
T2D	9	20662703	T	C	0.2460	211793
T2D	9	20790622	T	C	0.7437	211793
T2D	9	22029547	T	C	0.8633	211793
T2D	9	22132878	T	C	0.5630	211793
T2D	9	22134094	T	C	0.5638	211793
T2D	9	22137685	T	G	0.6253	211793
T2D	9	22157908	A	C	0.6801	211793
T2D	9	28772700	A	T	0.5810	211793
T2D	9	34074476	T	C	0.4232	211793
T2D	9	81359113	C	G	0.6394	211793
T2D	9	81905590	A	G	0.9442	211793
T2D	9	81917127	T	C	0.9448	211793
T2D	9	84311800	T	C	0.3886	211793
T2D	9	85316592	A	G	0.6940	211793
T2D	9	87387622	A	G	0.2916	211793
T2D	9	93633240	A	C	0.8092	211793
T2D	9	94395860	A	T	0.9146	211793
T2D	9	97001682	A	C	0.8842	211793
T2D	9	97050286	T	C	0.6743	211793
T2D	9	97497494	T	C	0.9394	211039
T2D	9	97804641	A	C	0.8668	211793
T2D	9	112526289	C	G	0.7492	211793
T2D	9	116942415	T	C	0.0289	211793
T2D	9	116943357	A	G	0.0291	211793
T2D	9	119252277	T	G	0.6437	211793
T2D	9	136149229	T	C	0.5552	211793
T2D	9	136149500	T	C	0.5555	211793
T2D	9	139241030	A	G	0.0891	201808
T2D	9	139244376	T	G	0.0833	203692
T2D	10	12309139	C	G	0.5168	211793
T2D	10	12328010	A	G	0.8465	208498
T2D	10	13540869	A	C	0.3272	211793
T2D	10	13566204	A	G	0.8583	211793
T2D	10	33997227	A	C	0.7805	211793

T2D	10	34018730	T	C	0.7921	211793
T2D	10	70382179	A	G	0.4880	211793
T2D	10	71321279	A	G	0.3605	211793
T2D	10	71452285	T	C	0.0754	211793
T2D	10	71466578	T	G	0.1045	211793
T2D	10	75599127	A	G	0.1200	211793
T2D	10	77310016	T	C	0.4849	211793
T2D	10	80947438	T	C	0.5650	207126
T2D	10	89722731	T	C	0.6957	211793
T2D	10	93956552	T	G	0.4833	211793
T2D	10	94462427	T	C	0.2913	211793
T2D	10	94466439	A	G	0.1339	211793
T2D	10	99056921	C	G	0.3058	211793
T2D	10	99061386	C	G	0.3254	211793
T2D	10	100421841	A	G	0.1661	211793
T2D	10	101820665	A	G	0.9353	211793
T2D	10	104563743	C	G	0.8815	211793
T2D	10	114703136	T	C	0.9294	211793
T2D	10	114743091	A	G	0.9754	209284
T2D	10	114754071	T	C	0.9618	209301
T2D	10	114754088	T	C	0.9618	209301
T2D	10	114757956	C	G	0.9712	205946
T2D	10	114871594	A	G	0.9625	200110
T2D	10	118558736	A	T	0.3516	211793
T2D	10	121660400	A	C	0.9238	211039
T2D	10	122849667	A	G	0.8031	211793
T2D	10	122929493	T	C	0.3359	211793
T2D	10	124193181	T	G	0.3963	211793
T2D	10	124195485	A	G	0.3975	211793
T2D	10	132947962	C	G	0.1316	211793
T2D	11	2118860	T	G	0.4812	207126
T2D	11	2197286	A	G	0.0816	206184
T2D	11	2203154	T	C	0.9181	206184
T2D	11	2270342	A	G	0.9603	210460
T2D	11	2372356	T	C	0.7179	211793
T2D	11	2579163	A	G	0.9539	206547
T2D	11	2632430	C	G	0.9475	211793
T2D	11	2642037	A	G	0.9138	211793
T2D	11	2691471	A	G	0.1066	211039
T2D	11	2705343	A	T	0.1813	206372
T2D	11	2755548	A	G	0.7402	207126
T2D	11	2849530	A	C	0.6196	207126
T2D	11	2857194	A	C	0.6579	207126
T2D	11	2908754	A	G	0.8660	206372
T2D	11	8654528	T	C	0.6186	211793
T2D	11	17409572	T	C	0.3760	211793
T2D	11	17415190	C	G	0.3846	211793
T2D	11	27729505	A	G	0.5655	211793

T2D	11	34642668	A	G	0.5839	211793
T2D	11	34908780	T	C	0.2882	211793
T2D	11	45768401	T	G	0.2941	211793
T2D	11	45846498	C	G	0.8121	211793
T2D	11	45912013	A	G	0.7753	211793
T2D	11	49351026	A	C	0.8245	211793
T2D	11	50110597	T	C	0.7643	211793
T2D	11	58128015	T	C	0.8186	211793
T2D	11	61565908	T	C	0.5575	152458
T2D	11	64100776	A	G	0.7167	207126
T2D	11	72460398	A	C	0.9455	211793
T2D	11	72460762	A	T	0.9456	211793
T2D	11	74625997	T	C	0.8260	211793
T2D	11	93014343	T	G	0.4536	211793
T2D	11	95710493	A	C	0.6267	211793
T2D	11	117693255	T	C	0.4701	211793
T2D	11	128042575	T	C	0.7156	211793
T2D	11	128234144	A	G	0.2633	211793
T2D	12	4300172	T	C	0.8693	211793
T2D	12	4374373	A	G	0.9546	211039
T2D	12	4399050	A	G	0.4291	208438
T2D	12	4406281	A	G	0.4699	207859
T2D	12	6302626	T	C	0.6386	211793
T2D	12	6681786	A	G	0.9828	208283
T2D	12	12871099	T	G	0.9684	200337
T2D	12	21781181	A	G	0.8754	211793
T2D	12	26457650	A	T	0.4664	211793
T2D	12	27963507	T	G	0.6714	211793
T2D	12	27963676	C	G	0.6712	211793
T2D	12	31367856	A	C	0.4785	211793
T2D	12	31441179	A	C	0.8301	211793
T2D	12	33409575	A	G	0.1893	211793
T2D	12	33410855	T	G	0.1852	211793
T2D	12	41838235	T	C	0.5689	211793
T2D	12	51357542	A	G	0.4985	211793
T2D	12	54541750	A	G	0.6297	211793
T2D	12	57146069	T	G	0.7839	211793
T2D	12	61250814	A	T	0.2313	211793
T2D	12	63788021	C	G	0.8750	211793
T2D	12	66221060	A	T	0.8769	211793
T2D	12	66250940	T	G	0.3404	211793
T2D	12	66326943	A	G	0.1376	211793
T2D	12	66363070	T	G	0.2037	211793
T2D	12	71520761	T	G	0.6240	211793
T2D	12	71634794	A	G	0.2382	211793
T2D	12	77398721	A	T	0.8867	211793
T2D	12	97848775	A	G	0.9461	211793
T2D	12	97849120	T	C	0.3326	211793

T2D	12	101218303	A	T	0.9182	211793
T2D	12	114123722	C	G	0.5728	211793
T2D	12	117723613	A	G	0.1449	211793
T2D	12	118394008	T	C	0.7068	211793
T2D	12	118489636	A	T	0.8691	211039
T2D	12	120989183	T	C	0.2092	211793
T2D	12	121429194	T	C	0.2828	211793
T2D	12	123447928	T	C	0.3254	211793
T2D	12	123493123	A	G	0.8630	211793
T2D	12	123640853	C	G	0.0257	211039
T2D	12	124428331	T	C	0.8777	211039
T2D	12	133777466	A	G	0.7835	211039
T2D	13	22589883	A	G	0.3505	211793
T2D	13	26776999	A	G	0.5649	211793
T2D	13	26784939	T	C	0.5653	211793
T2D	13	31036642	C	G	0.8378	211793
T2D	13	33507267	A	C	0.9270	211039
T2D	13	33554405	T	C	0.1584	211039
T2D	13	33557173	A	T	0.8428	211039
T2D	13	41688401	A	G	0.6822	211793
T2D	13	46514492	C	G	0.6000	211793
T2D	13	51094114	T	G	0.3794	211793
T2D	13	51096095	A	T	0.3815	211793
T2D	13	58656599	T	C	0.5964	211793
T2D	13	59077406	A	T	0.4328	211793
T2D	13	66204880	T	C	0.7807	211793
T2D	13	80707429	A	G	0.7185	211793
T2D	13	91949562	A	G	0.8310	211793
T2D	13	106074896	T	C	0.3158	211039
T2D	13	109947213	T	C	0.5481	211793
T2D	13	112187882	A	T	0.8396	211793
T2D	14	23288935	C	G	0.9464	211793
T2D	14	29744532	A	G	0.4400	211793
T2D	14	30086481	T	C	0.3667	211793
T2D	14	35390146	T	C	0.1493	211793
T2D	14	35409701	A	T	0.1905	211793
T2D	14	38804675	T	C	0.4569	211793
T2D	14	38809661	A	G	0.4569	211793
T2D	14	69459229	A	C	0.1057	211793
T2D	14	74932641	A	T	0.7578	211793
T2D	14	77382503	A	G	0.6732	207126
T2D	14	91963722	A	G	0.4878	211793
T2D	14	101255172	A	G	0.7556	206547
T2D	14	103860309	T	G	0.1634	211793
T2D	14	103960026	A	G	0.8398	211793
T2D	15	28546173	T	C	0.7322	174183
T2D	15	36392562	A	G	0.1621	211793
T2D	15	38822905	T	C	0.4283	211793

T2D	15	38834033	T	C	0.4114	211793
T2D	15	40398754	T	C	0.2422	211793
T2D	15	40619724	T	C	0.7698	206372
T2D	15	43895118	T	C	0.6914	211793
T2D	15	49794020	T	C	0.5033	211793
T2D	15	53099306	T	C	0.9560	211793
T2D	15	57333416	A	G	0.4153	211039
T2D	15	62383155	T	C	0.4930	211793
T2D	15	62386795	A	C	0.9688	211793
T2D	15	62396389	A	G	0.5839	211793
T2D	15	63823301	A	G	0.9374	211793
T2D	15	68080886	A	T	0.2214	211793
T2D	15	74328576	A	G	0.3477	211793
T2D	15	74331659	A	C	0.8731	211793
T2D	15	75742095	A	C	0.6081	211793
T2D	15	75928192	T	G	0.5613	211793
T2D	15	77776562	A	C	0.6051	211793
T2D	15	77782335	A	G	0.5952	211793
T2D	15	80432222	A	G	0.8913	211793
T2D	15	90423293	T	C	0.2087	211039
T2D	15	90428894	A	C	0.2010	211039
T2D	15	91544076	A	G	0.0196	208438
T2D	15	93825384	A	G	0.4379	211793
T2D	16	295795	T	C	0.6082	211793
T2D	16	300388	T	C	0.5965	211793
T2D	16	967241	A	G	0.7291	206372
T2D	16	15153717	T	C	0.2110	211039
T2D	16	28913787	T	C	0.1361	211793
T2D	16	30045789	C	G	0.6585	207126
T2D	16	53756885	A	G	0.9673	211214
T2D	16	53770578	A	T	0.9687	211214
T2D	16	53800954	T	C	0.8332	211793
T2D	16	69657996	T	G	0.8719	211793
T2D	16	70660243	A	G	0.5878	211793
T2D	16	71634811	A	T	0.2812	211039
T2D	16	75247245	T	G	0.7968	211793
T2D	16	81534790	T	C	0.3635	211793
T2D	16	85716463	A	G	0.4852	211039
T2D	16	88132199	A	G	0.1869	207126
T2D	16	88554480	T	C	0.3030	202590
T2D	17	3828086	C	G	0.5485	200337
T2D	17	3860356	A	T	0.7020	200337
T2D	17	3947644	T	C	0.6298	211793
T2D	17	3988451	T	C	0.7451	211793
T2D	17	6947453	C	G	0.9090	211793
T2D	17	7531965	T	C	0.5768	211793
T2D	17	17661802	A	G	0.8451	207126
T2D	17	17715317	C	G	0.8298	206372

T2D	17	27562990	T	C	0.1690	211039
T2D	17	29642430	T	C	0.5346	211039
T2D	17	34842521	A	G	0.5338	211793
T2D	17	34952964	A	T	0.6045	211793
T2D	17	36101586	A	C	0.3049	211214
T2D	17	36102381	A	G	0.3038	211214
T2D	17	37746307	T	C	0.4383	211039
T2D	17	40722029	A	G	0.5664	211039
T2D	17	41456413	A	G	0.7477	207126
T2D	17	46989154	A	G	0.7497	211793
T2D	17	61564052	A	G	0.3595	207126
T2D	17	65648427	T	G	0.9682	210940
T2D	17	65825248	T	C	0.3371	211793
T2D	17	75386909	A	G	0.2968	207126
T2D	17	78757626	A	G	0.6908	211039
T2D	18	4845027	A	G	0.6120	211214
T2D	18	7070642	T	C	0.3013	211793
T2D	18	13271367	A	G	0.5008	211793
T2D	18	21839152	A	T	0.9613	211039
T2D	18	31582890	T	C	0.9111	211793
T2D	18	53452144	T	C	0.9859	211694
T2D	18	56879827	T	C	0.8340	211793
T2D	18	57848369	A	T	0.7791	211793
T2D	18	57852587	T	C	0.7570	211793
T2D	18	60668270	A	G	0.4463	211793
T2D	18	60845884	T	C	0.5122	211793
T2D	18	60903978	C	G	0.5669	211793
T2D	18	74582340	T	C	0.4386	211039
T2D	19	7235146	A	G	0.9942	210721
T2D	19	7293119	T	C	0.1277	211039
T2D	19	7970635	A	G	0.4263	207126
T2D	19	7986638	C	G	0.4816	202938
T2D	19	12496934	T	C	0.7449	210460
T2D	19	12505873	A	G	0.7416	210460
T2D	19	18860041	A	G	0.9656	205793
T2D	19	33890838	C	G	0.5391	206372
T2D	19	39580737	A	G	0.6794	211793
T2D	19	45411941	T	C	0.8997	206547
T2D	19	46157019	A	G	0.7229	206547
T2D	19	46178661	T	C	0.3029	206547
T2D	19	47580185	A	G	0.7148	211793
T2D	19	47597102	T	C	0.7310	211793
T2D	20	21466795	T	C	0.8043	211793
T2D	20	22435749	A	C	0.3291	211793
T2D	20	32675727	A	G	0.2058	211793
T2D	20	39681683	C	G	0.9379	211039
T2D	20	42230695	T	C	0.2389	211039
T2D	20	42993328	T	C	0.8237	211793

T2D	20	42994812	T	C	0.5580	211793
T2D	20	48830772	A	T	0.5132	211793
T2D	20	50155386	T	C	0.4101	211793
T2D	20	50999627	T	C	0.3286	211793
T2D	20	51223594	A	T	0.8500	211793
T2D	20	51620857	A	G	0.8755	211039
T2D	20	57394628	C	G	0.3903	211793
T2D	20	57396495	T	C	0.3810	211793
T2D	20	61277014	A	T	0.2872	183611
T2D	20	62450664	T	C	0.4247	183611
T2D	21	33385186	A	G	0.5303	211793
T2D	22	29369398	A	C	0.5071	211793
T2D	22	32092981	A	T	0.9672	210940
T2D	22	35705359	A	G	0.3920	211793
T2D	22	38599767	C	G	0.3564	211793
T2D	22	40541838	T	C	0.6678	211793
T2D	22	41593873	T	G	0.2112	211039
T2D	22	44377442	A	G	0.5035	211793
T2D	22	50435480	A	G	0.4658	207126
T2D	22	50440296	T	C	0.4646	207126
T2D	22	50604696	A	G	0.8605	207126

BETA	SE	P
-0.0070	0.0051	1.685E-01
0.0053	0.0045	2.333E-01
-0.0048	0.0049	3.235E-01
-0.0215	0.0041	1.466E-07
-0.0024	0.0041	5.518E-01
0.0584	0.0065	3.039E-19
-0.0615	0.0067	6.003E-20
0.0069	0.0052	1.857E-01
0.0132	0.0056	1.855E-02
-0.0287	0.0043	1.515E-11
0.0258	0.0043	1.985E-09
0.0026	0.0045	5.583E-01
-0.0309	0.0085	2.831E-04
-0.0299	0.0086	4.882E-04
-0.0243	0.0065	2.016E-04
-0.0431	0.0045	1.724E-21
-0.0181	0.0076	1.713E-02
-0.0203	0.0068	3.002E-03
-0.0092	0.0082	2.591E-01
-0.0005	0.0089	9.578E-01
-0.0100	0.0078	2.006E-01
0.0159	0.0070	2.209E-02
-0.0229	0.0041	2.025E-08
-0.0355	0.0045	2.743E-15
0.0352	0.0045	4.291E-15
-0.0026	0.0046	5.678E-01
0.0083	0.0042	4.886E-02
0.0107	0.0049	3.019E-02
-0.0051	0.0042	2.252E-01
0.0011	0.0042	7.835E-01
-0.0176	0.0041	1.762E-05
0.0190	0.0041	3.072E-06
-0.0078	0.0049	1.125E-01
0.0118	0.0042	5.441E-03
0.0032	0.0050	5.144E-01
0.0094	0.0054	8.116E-02
0.0085	0.0043	4.637E-02
0.0078	0.0046	9.170E-02
-0.0344	0.0056	7.471E-10
-0.0103	0.0046	2.408E-02
0.0498	0.0055	1.904E-19
-0.0339	0.0047	8.453E-13
-0.0055	0.0061	3.632E-01
-0.0161	0.0045	3.453E-04
0.0236	0.0051	4.242E-06
0.0195	0.0046	2.606E-05
-0.0189	0.0047	5.257E-05

-0.0069	0.0049	1.607E-01
-0.0104	0.0044	1.709E-02
0.0314	0.0046	1.076E-11
-0.0313	0.0047	2.218E-11
-0.0172	0.0065	7.900E-03
0.0052	0.0048	2.756E-01
0.0149	0.0045	8.876E-04
0.0043	0.0041	2.883E-01
-0.0016	0.0042	7.036E-01
-0.0113	0.0059	5.627E-02
0.0117	0.0042	5.575E-03
0.0022	0.0044	6.125E-01
-0.0177	0.0043	4.489E-05
0.0183	0.0043	2.518E-05
-0.0063	0.0044	1.528E-01
-0.0047	0.0079	5.480E-01
-0.0027	0.0042	5.141E-01
0.0115	0.0046	1.153E-02
-0.0058	0.0046	2.028E-01
0.0099	0.0063	1.150E-01
0.0174	0.0049	3.573E-04
-0.0015	0.0086	8.612E-01
0.0029	0.0042	4.949E-01
0.0077	0.0042	6.591E-02
0.0020	0.0047	6.750E-01
0.0191	0.0068	4.843E-03
-0.0142	0.0045	1.530E-03
0.0043	0.0041	2.989E-01
-0.0018	0.0097	8.561E-01
-0.0208	0.0053	9.073E-05
0.0043	0.0042	3.089E-01
0.0312	0.0096	1.233E-03
-0.0374	0.0088	2.358E-05
0.0388	0.0085	5.281E-06
-0.0146	0.0045	1.126E-03
-0.0137	0.0054	1.170E-02
0.0180	0.0041	9.986E-06
-0.0042	0.0044	3.400E-01
-0.0121	0.0142	3.954E-01
0.0258	0.0048	7.913E-08
-0.0279	0.0094	2.859E-03
0.0122	0.0097	2.079E-01
0.0007	0.0065	9.109E-01
0.0197	0.0059	8.586E-04
0.0134	0.0043	1.811E-03
-0.0264	0.0047	1.504E-08
-0.0264	0.0047	1.605E-08
0.0124	0.0050	1.232E-02

0.0154	0.0055	5.203E-03
0.0153	0.0055	5.373E-03
-0.0005	0.0044	9.066E-01
0.0474	0.0134	4.223E-04
-0.0084	0.0048	7.858E-02
0.0077	0.0085	3.607E-01
-0.0101	0.0108	3.516E-01
-0.0335	0.0082	4.681E-05
0.0076	0.0041	6.460E-02
-0.0006	0.0113	9.574E-01
-0.0069	0.0073	3.429E-01
0.0058	0.0071	4.102E-01
0.0053	0.0045	2.356E-01
0.0231	0.0062	2.221E-04
-0.0384	0.0204	6.026E-02
-0.0029	0.0115	8.000E-01
-0.0054	0.0047	2.472E-01
0.0089	0.0042	3.340E-02
-0.0095	0.0042	2.215E-02
-0.0110	0.0054	4.161E-02
-0.0067	0.0088	4.466E-01
0.0469	0.0131	3.292E-04
-0.0038	0.0041	3.513E-01
-0.0508	0.0135	1.630E-04
-0.0224	0.0101	2.706E-02
-0.0434	0.0115	1.642E-04
-0.0015	0.0053	7.747E-01
-0.0231	0.0066	4.739E-04
0.0200	0.0057	4.066E-04
0.0100	0.0041	1.534E-02
-0.0094	0.0042	2.466E-02
-0.0019	0.0044	6.668E-01
0.0104	0.0069	1.313E-01
0.0077	0.0049	1.147E-01
0.0052	0.0042	2.148E-01
0.0012	0.0045	7.869E-01
-0.0063	0.0049	1.951E-01
0.0081	0.0059	1.718E-01
0.0025	0.0066	7.042E-01
0.0040	0.0064	5.379E-01
-0.0030	0.0041	4.704E-01
-0.0019	0.0055	7.255E-01
-0.0051	0.0041	2.160E-01
0.0072	0.0042	8.732E-02
-0.0202	0.0041	8.234E-07
-0.0193	0.0043	5.879E-06
-0.0198	0.0045	9.039E-06
-0.0020	0.0054	7.077E-01

-0.0105	0.0043	1.451E-02
0.0243	0.0051	2.362E-06
-0.0268	0.0181	1.393E-01
0.0354	0.0048	1.948E-13
-0.0029	0.0049	5.518E-01
0.0124	0.0042	3.547E-03
-0.0114	0.0054	3.538E-02
-0.0045	0.0043	2.957E-01
0.0094	0.0046	3.865E-02
0.0114	0.0045	1.083E-02
-0.0098	0.0042	2.043E-02
-0.0142	0.0048	3.057E-03
-0.0032	0.0045	4.774E-01
-0.0051	0.0067	4.461E-01
0.0006	0.0064	9.226E-01
-0.0032	0.0041	4.367E-01
0.0125	0.0041	2.120E-03
-0.0126	0.0041	2.052E-03
0.0191	0.0071	7.023E-03
-0.0194	0.0071	6.229E-03
0.0081	0.0136	5.517E-01
0.0183	0.0044	3.444E-05
-0.0171	0.0055	1.859E-03
-0.0144	0.0041	4.575E-04
0.0000	0.0130	9.996E-01
-0.0109	0.0046	1.639E-02
-0.0061	0.0042	1.461E-01
0.0042	0.0107	6.970E-01
0.0010	0.0047	8.229E-01
0.0224	0.0060	1.801E-04
0.0023	0.0048	6.283E-01
0.0176	0.0045	8.544E-05
-0.0048	0.0051	3.471E-01
-0.0058	0.0045	1.949E-01
-0.0054	0.0102	5.948E-01
0.0187	0.0047	7.850E-05
0.0179	0.0047	1.269E-04
0.0060	0.0042	1.576E-01
-0.0071	0.0075	3.497E-01
-0.0042	0.0063	5.115E-01
-0.0185	0.0041	5.445E-06
-0.0009	0.0061	8.814E-01
-0.0279	0.0123	2.326E-02
0.0070	0.0057	2.231E-01
-0.0035	0.0047	4.507E-01
0.0057	0.0045	2.120E-01
0.0018	0.0085	8.286E-01
0.0184	0.0119	1.221E-01

0.0061	0.0046	1.784E-01
0.0058	0.0045	1.926E-01
-0.0242	0.0050	1.328E-06
0.0135	0.0063	3.199E-02
0.0138	0.0043	1.275E-03
0.0113	0.0042	6.718E-03
-0.0294	0.0092	1.413E-03
-0.0204	0.0055	1.938E-04
-0.0330	0.0128	9.823E-03
0.0222	0.0043	1.699E-07
0.0092	0.0045	4.244E-02
-0.0047	0.0057	4.077E-01
0.0194	0.0101	5.349E-02
-0.0137	0.0044	1.894E-03
0.0127	0.0060	3.432E-02
-0.0242	0.0043	1.933E-08
-0.0376	0.0048	5.880E-15
0.0010	0.0058	8.669E-01
0.0006	0.0053	9.023E-01
0.0069	0.0042	9.629E-02
-0.0131	0.0042	1.790E-03
-0.0196	0.0196	3.167E-01
0.0137	0.0047	3.732E-03
-0.0084	0.0042	4.711E-02
0.0066	0.0041	1.070E-01
-0.0062	0.0046	1.798E-01
0.0172	0.0042	4.236E-05
0.0025	0.0042	5.532E-01
-0.0208	0.0046	6.564E-06
-0.0212	0.0046	4.577E-06
-0.0138	0.0057	1.584E-02
0.0077	0.0105	4.641E-01
-0.0151	0.0072	3.685E-02
0.0146	0.0057	1.000E-02
0.0107	0.0041	8.623E-03
-0.0147	0.0126	2.420E-01
-0.0251	0.0064	8.765E-05
0.0215	0.0052	3.202E-05
0.0205	0.0048	2.341E-05
-0.0084	0.0041	4.039E-02
0.0086	0.0043	4.482E-02
-0.0056	0.0042	1.797E-01
0.0040	0.0043	3.440E-01
-0.0295	0.0041	5.586E-13
-0.0278	0.0041	1.339E-11
-0.0117	0.0042	5.902E-03
0.0149	0.0043	4.404E-04
0.0083	0.0052	1.086E-01

0.0113	0.0052	2.979E-02
0.0074	0.0052	1.559E-01
-0.0100	0.0041	1.487E-02
0.0385	0.0043	3.276E-19
-0.0311	0.0042	7.467E-14
-0.0514	0.0067	1.566E-14
0.0028	0.0075	7.039E-01
0.0110	0.0098	2.628E-01
0.0235	0.0060	8.129E-05
-0.0059	0.0050	2.364E-01
0.0227	0.0069	9.683E-04
0.0273	0.0045	1.317E-09
-0.0273	0.0045	1.248E-09
-0.0093	0.0052	7.601E-02
-0.0122	0.0055	2.760E-02
-0.0138	0.0054	1.038E-02
-0.0140	0.0054	9.413E-03
-0.0122	0.0043	4.438E-03
-0.0040	0.0060	5.041E-01
-0.0183	0.0046	7.479E-05
0.0202	0.0045	7.378E-06
0.0057	0.0061	3.506E-01
-0.1170	0.0202	7.553E-09
-0.1193	0.0204	5.219E-09
-0.0064	0.0044	1.451E-01
-0.0028	0.0078	7.210E-01
-0.0212	0.0173	2.212E-01
-0.0204	0.0048	1.803E-05
0.0143	0.0102	1.598E-01
-0.0066	0.0048	1.667E-01
0.0190	0.0086	2.790E-02
0.0524	0.0062	2.343E-17
-0.1692	0.0069	2.300E-132
-0.1775	0.0061	2.110E-184
0.1323	0.0050	2.600E-155
0.1084	0.0047	1.880E-116
0.0297	0.0051	7.066E-09
-0.0085	0.0050	8.740E-02
0.0100	0.0045	2.734E-02
-0.0011	0.0043	7.910E-01
-0.0028	0.0109	7.943E-01
0.0356	0.0149	1.697E-02
-0.0277	0.0042	2.377E-11
0.0170	0.0045	1.675E-04
-0.0186	0.0064	3.817E-03
0.0008	0.0045	8.582E-01
-0.0037	0.0097	6.989E-01
0.0128	0.0054	1.715E-02

-0.0245	0.0058	2.231E-05
0.0016	0.0042	6.924E-01
-0.0530	0.0051	1.342E-25
0.0527	0.0051	2.313E-25
0.0235	0.0042	1.974E-08
0.0329	0.0044	6.764E-14
0.0421	0.0053	1.228E-15
0.0364	0.0045	7.025E-16
0.0110	0.0043	1.133E-02
-0.0180	0.0051	3.541E-04
-0.0160	0.0044	2.455E-04
-0.0023	0.0063	7.187E-01
0.0083	0.0042	4.712E-02
-0.0143	0.0041	5.020E-04
0.0561	0.0122	4.531E-06
0.0519	0.0119	1.330E-05
-0.0040	0.0045	3.680E-01
-0.0038	0.0049	4.413E-01
0.0433	0.0149	3.610E-03
0.0133	0.0050	8.091E-03
0.0135	0.0050	6.960E-03
-0.0010	0.0047	8.301E-01
-0.0044	0.0064	4.878E-01
-0.0043	0.0095	6.492E-01
-0.0117	0.0041	4.443E-03
0.0037	0.0051	4.651E-01
-0.0137	0.0047	3.714E-03
0.0450	0.0043	8.259E-26
-0.0512	0.0091	1.837E-08
0.0643	0.0131	8.554E-07
0.0572	0.0045	3.367E-37
0.0492	0.0077	1.532E-10
0.0636	0.0079	8.241E-16
0.1074	0.0047	4.400E-116
-0.1128	0.0047	1.390E-127
-0.0221	0.0046	1.272E-06
0.0119	0.0044	6.543E-03
-0.0127	0.0042	2.359E-03
0.0076	0.0042	6.601E-02
0.0079	0.0043	6.583E-02
-0.0049	0.0049	3.182E-01
-0.0066	0.0046	1.535E-01
-0.0124	0.0043	4.235E-03
-0.0078	0.0050	1.155E-01
-0.0076	0.0044	8.725E-02
-0.0066	0.0063	2.931E-01
0.0017	0.0044	6.949E-01
0.0070	0.0044	1.156E-01

-0.0262	0.0072	2.698E-04
0.0048	0.0050	3.337E-01
-0.0211	0.0042	5.178E-07
0.0126	0.0046	5.660E-03
0.0057	0.0089	5.197E-01
0.0038	0.0044	3.875E-01
0.0049	0.0042	2.431E-01
-0.0080	0.0045	7.842E-02
0.0367	0.0046	1.327E-15
0.0319	0.0070	4.952E-06
0.0064	0.0047	1.753E-01
-0.0065	0.0088	4.576E-01
0.0077	0.0089	3.885E-01
0.0420	0.0044	2.991E-21
0.0414	0.0044	5.339E-21
-0.0358	0.0045	1.400E-15
-0.0393	0.0042	9.661E-21
0.0115	0.0051	2.425E-02
0.0208	0.0208	3.175E-01
-0.0109	0.0046	1.784E-02
-0.0129	0.0080	1.078E-01
0.0333	0.0044	2.239E-14
0.0064	0.0042	1.324E-01
0.0100	0.0044	2.154E-02
-0.0198	0.0080	1.356E-02
0.0005	0.0044	9.005E-01
0.0032	0.0080	6.941E-01
-0.0094	0.0051	6.543E-02
0.0132	0.0041	1.186E-03
-0.0024	0.0065	7.108E-01
-0.0097	0.0061	1.104E-01
-0.0090	0.0041	2.680E-02
0.0017	0.0054	7.600E-01
0.0133	0.0049	6.296E-03
-0.0050	0.0093	5.894E-01
-0.0047	0.0054	3.847E-01
-0.0121	0.0041	3.526E-03
-0.0296	0.0046	1.269E-10
0.0271	0.0043	2.193E-10
0.0214	0.0044	9.653E-07
0.0021	0.0041	6.180E-01
0.0057	0.0052	2.705E-01
-0.0020	0.0061	7.480E-01
0.0014	0.0058	8.114E-01
0.0005	0.0056	9.227E-01
-0.0036	0.0061	5.571E-01
0.0052	0.0082	5.277E-01
-0.0021	0.0066	7.534E-01

-0.0012	0.0065	8.476E-01
0.0055	0.0048	2.544E-01
-0.0057	0.0048	2.291E-01
0.0082	0.0101	4.158E-01
-0.0146	0.0045	1.032E-03
0.0253	0.0041	5.432E-10
-0.0074	0.0046	1.119E-01
0.0127	0.0044	3.638E-03
-0.0089	0.0054	1.006E-01
0.0021	0.0050	6.697E-01
0.0258	0.0046	2.610E-08
0.0277	0.0047	3.706E-09
-0.0116	0.0042	6.521E-03
0.0184	0.0079	2.022E-02
0.0050	0.0046	2.752E-01
0.0177	0.0062	4.230E-03
-0.0265	0.0067	7.832E-05
-0.1439	0.0049	5.240E-193
-0.1211	0.0049	2.820E-135
-0.1390	0.0051	1.630E-163
0.1270	0.0049	4.830E-147
-0.3124	0.0075	3.34E-376
0.1253	0.0093	2.085E-41
0.0203	0.0049	3.180E-05
0.0155	0.0063	1.351E-02
0.0831	0.0057	4.535E-48
-0.0214	0.0059	3.065E-04
-0.0246	0.0064	1.276E-04
0.0160	0.0041	9.492E-05
0.0075	0.0042	7.257E-02
-0.0226	0.0060	1.765E-04
0.0248	0.0058	1.749E-05
0.0253	0.0058	1.159E-05
0.0263	0.0064	3.984E-05
0.0063	0.0059	2.859E-01
0.0138	0.0046	2.722E-03
0.0136	0.0042	1.409E-03
-0.0251	0.0042	2.605E-09
0.0209	0.0044	2.379E-06
-0.0236	0.0044	9.977E-08
0.0246	0.0045	6.264E-08
-0.0414	0.0059	3.030E-12
0.0247	0.0085	3.468E-03
0.0127	0.0047	7.080E-03
0.0143	0.0047	2.402E-03
-0.0135	0.0113	2.321E-01
-0.0040	0.0078	6.061E-01
0.0041	0.0044	3.556E-01

0.0130	0.0048	6.559E-03
-0.0024	0.0071	7.301E-01
-0.0181	0.0042	1.663E-05
-0.0233	0.0048	1.554E-06
0.0198	0.0059	7.699E-04
0.0489	0.0071	5.392E-12
0.0490	0.0070	1.935E-12
-0.0088	0.0046	5.577E-02
-0.0029	0.0042	4.854E-01
-0.0002	0.0042	9.640E-01
0.0041	0.0042	3.252E-01
0.0043	0.0115	7.123E-01
-0.0041	0.0045	3.633E-01
-0.0103	0.0041	1.221E-02
-0.0171	0.0044	9.905E-05
-0.0150	0.0044	6.866E-04
0.0095	0.0049	5.000E-02
-0.0936	0.0054	1.682E-66
0.0032	0.0041	4.420E-01
0.0467	0.0112	3.220E-05
0.0224	0.0041	5.682E-08
0.0407	0.0045	7.145E-20
-0.0423	0.0045	3.350E-21
-0.0220	0.0065	7.612E-04
-0.0161	0.0041	9.171E-05
-0.0070	0.0052	1.785E-01
0.0011	0.0069	8.754E-01
0.0108	0.0069	1.161E-01
-0.0444	0.0044	5.876E-24
-0.0002	0.0049	9.610E-01
-0.0268	0.0045	1.725E-09
-0.0503	0.0088	9.569E-09
0.0490	0.0043	1.998E-30
0.0431	0.0052	1.241E-16
-0.0437	0.0050	2.900E-18
0.0042	0.0042	3.216E-01
-0.0026	0.0042	5.381E-01
0.0156	0.0075	3.805E-02
0.0057	0.0045	2.069E-01
0.0180	0.0057	1.559E-03
-0.0043	0.0062	4.864E-01
0.0109	0.0053	3.872E-02
-0.0252	0.0055	4.810E-06
0.0006	0.0055	9.188E-01
0.0000	0.0051	9.928E-01
0.0091	0.0067	1.733E-01
-0.0025	0.0041	5.468E-01
-0.0174	0.0073	1.681E-02

-0.0096	0.0046	3.660E-02
-0.0144	0.0048	2.471E-03
-0.0051	0.0041	2.114E-01
-0.0133	0.0042	1.652E-03
-0.0044	0.0066	5.049E-01
-0.0110	0.0063	7.779E-02
0.0143	0.0042	6.852E-04
-0.0010	0.0043	8.201E-01
-0.0204	0.0041	8.698E-07
-0.0470	0.0052	3.096E-19
0.0069	0.0042	1.059E-01
-0.0241	0.0071	6.336E-04
-0.0036	0.0045	4.193E-01
-0.0051	0.0046	2.682E-01
0.0114	0.0109	2.990E-01
0.0008	0.0048	8.657E-01
0.0027	0.0103	7.961E-01
0.0055	0.0053	2.995E-01
0.0139	0.0041	6.499E-04
-0.0898	0.0063	9.460E-47
-0.0575	0.0041	4.995E-44
-0.0731	0.0059	6.939E-35
0.1023	0.0042	3.960E-130
-0.1198	0.0041	1.070E-189
0.0837	0.0043	8.236E-85
0.0511	0.0059	5.765E-18
-0.0267	0.0102	8.746E-03
-0.1626	0.0041	7.59E-343
-0.1625	0.0041	1.01E-342
0.0918	0.0042	1.000E-103
0.0101	0.0042	1.700E-02
-0.0009	0.0043	8.330E-01
-0.0043	0.0048	3.679E-01
0.0063	0.0041	1.269E-01
-0.0190	0.0179	2.883E-01
-0.0097	0.0084	2.459E-01
0.0034	0.0050	4.865E-01
-0.0026	0.0042	5.371E-01
0.0096	0.0050	5.400E-02
-0.0017	0.0043	6.893E-01
-0.0197	0.0053	1.923E-04
0.0028	0.0041	5.015E-01
0.0034	0.0102	7.350E-01
-0.0130	0.0056	2.071E-02
-0.0046	0.0062	4.573E-01
-0.0046	0.0043	2.802E-01
0.0024	0.0052	6.474E-01
0.0065	0.0056	2.401E-01

0.0074	0.0061	2.259E-01
-0.0167	0.0061	6.079E-03
0.0621	0.0072	1.024E-17
-0.0030	0.0045	5.002E-01
0.0135	0.0062	2.823E-02
-0.0645	0.0150	1.727E-05
-0.0311	0.0067	3.980E-06
-0.3133	0.0054	1.58E-745
0.0945	0.0041	4.940E-119
-0.1943	0.0054	2.760E-284
0.0334	0.0066	3.416E-07
0.0672	0.0051	5.328E-40
0.0076	0.0041	6.560E-02
-0.0023	0.0179	8.970E-01
-0.0352	0.0080	1.020E-05
-0.0468	0.0111	2.327E-05
0.0655	0.0056	1.159E-31
0.0102	0.0128	4.238E-01
-0.0409	0.0042	2.975E-22
0.0213	0.0071	2.930E-03
-0.0078	0.0046	9.180E-02
-0.0039	0.0104	7.094E-01
-0.0221	0.0111	4.627E-02
-0.0219	0.0111	4.782E-02
0.0032	0.0042	4.427E-01
0.0020	0.0053	7.095E-01
-0.0142	0.0080	7.729E-02
0.0025	0.0078	7.470E-01
0.0243	0.0041	3.668E-09
0.0084	0.0043	5.267E-02
0.0043	0.0059	4.628E-01
-0.0037	0.0049	4.423E-01
-0.0010	0.0042	8.124E-01
-0.0159	0.0044	3.111E-04
0.0089	0.0046	5.278E-02
-0.0050	0.0043	2.492E-01
-0.0046	0.0045	3.029E-01
-0.0188	0.0043	1.100E-05
0.0056	0.0068	4.091E-01
0.0198	0.0041	1.306E-06
0.0212	0.0043	7.130E-07
0.0115	0.0041	5.251E-03
0.0329	0.0041	1.523E-15
0.0054	0.0042	1.998E-01
0.0116	0.0044	8.833E-03
0.0098	0.0042	2.011E-02
-0.0104	0.0046	2.388E-02
0.0197	0.0041	1.714E-06

-0.0436	0.0047	1.587E-20
-0.0920	0.0042	6.600E-108
0.0688	0.0046	1.894E-50
-0.1189	0.0081	9.998E-49
-0.0653	0.0051	1.095E-37
-0.0409	0.0064	1.594E-10
0.0103	0.0041	1.278E-02
0.0335	0.0055	1.297E-09
-0.0151	0.0043	5.017E-04
0.0062	0.0046	1.790E-01
-0.0181	0.0052	5.485E-04
-0.0229	0.0048	1.452E-06
0.0123	0.0111	2.710E-01
0.0031	0.0044	4.825E-01
-0.0096	0.0042	2.114E-02
-0.0146	0.0076	5.485E-02
0.0093	0.0043	2.846E-02
-0.0053	0.0056	3.356E-01
0.0013	0.0058	8.271E-01
0.0160	0.0076	3.501E-02
0.0186	0.0074	1.146E-02
-0.0188	0.0043	1.104E-05
-0.0045	0.0059	4.496E-01
-0.0267	0.0046	4.456E-09
-0.0104	0.0056	6.454E-02
0.0057	0.0053	2.799E-01
0.0249	0.0041	1.137E-09
-0.0028	0.0050	5.742E-01
-0.0026	0.0043	5.397E-01
-0.0053	0.0055	3.284E-01
0.0082	0.0042	5.090E-02
-0.0172	0.0052	8.330E-04
0.0085	0.0042	4.400E-02
-0.0094	0.0074	1.996E-01
-0.0008	0.0052	8.766E-01
0.0788	0.0058	1.404E-42
0.0340	0.0045	2.724E-14
0.1463	0.0070	6.250E-98
0.1172	0.0083	1.901E-45
-0.0571	0.0041	1.267E-44
0.0123	0.0041	2.593E-03
0.0194	0.0051	1.296E-04
0.0189	0.0049	1.011E-04
0.0030	0.0045	5.077E-01
-0.0223	0.0051	1.263E-05
-0.0124	0.0041	2.553E-03
0.0081	0.0053	1.298E-01
0.0117	0.0057	3.997E-02

-0.0090	0.0043	3.723E-02
-0.0234	0.0041	9.427E-09
-0.0056	0.0095	5.525E-01
0.0136	0.0041	9.189E-04
0.0058	0.0059	3.281E-01
0.0084	0.0043	4.998E-02
-0.0112	0.0042	8.401E-03
-0.0331	0.0042	6.523E-15
-0.1107	0.0084	6.709E-40
0.0702	0.0064	3.479E-28
0.0971	0.0181	8.105E-08
-0.1108	0.0083	3.187E-40
0.0034	0.0043	4.337E-01
-0.0220	0.0057	1.110E-04
-0.0188	0.0047	6.491E-05
-0.0029	0.0046	5.351E-01
0.0042	0.0042	3.183E-01
0.0173	0.0051	6.324E-04
0.0089	0.0044	4.140E-02
0.0239	0.0045	1.230E-07
0.0015	0.0168	9.308E-01
-0.0087	0.0042	3.771E-02
-0.0110	0.0042	8.143E-03
-0.0272	0.0041	4.838E-11
-0.0355	0.0044	9.818E-16
-0.0069	0.0042	9.949E-02
0.0022	0.0052	6.711E-01
0.0301	0.0043	3.155E-12
0.0358	0.0041	4.908E-18
-0.0014	0.0044	7.574E-01
-0.0060	0.0069	3.872E-01
0.0350	0.0121	3.962E-03
-0.0139	0.0043	1.430E-03
-0.0232	0.0047	8.703E-07
0.0138	0.0041	7.275E-04
-0.0022	0.0045	6.253E-01
0.0046	0.0052	3.710E-01
0.0124	0.0041	2.606E-03
0.0085	0.0064	1.847E-01
0.0009	0.0047	8.457E-01
0.0023	0.0054	6.754E-01
0.0128	0.0043	2.841E-03
-0.0079	0.0067	2.367E-01
-0.0192	0.0073	8.094E-03
-0.0191	0.0073	8.943E-03
0.0003	0.0068	9.632E-01
0.0025	0.0043	5.683E-01
-0.0010	0.0046	8.367E-01

0.0327	0.0043	5.148E-14
0.0330	0.0066	5.677E-07
-0.0329	0.0044	6.032E-14
0.0045	0.0085	5.965E-01
0.0526	0.0059	3.977E-19
0.0482	0.0044	2.196E-27
-0.0477	0.0047	3.160E-24
-0.0248	0.0133	6.275E-02
-0.0516	0.0064	5.191E-16
0.0340	0.0048	8.695E-13
-0.0603	0.0090	2.366E-11
-0.0631	0.0090	2.059E-12
0.0073	0.0042	8.403E-02
0.0117	0.0054	3.010E-02
-0.0055	0.0049	2.693E-01
-0.0457	0.0120	1.386E-04
0.0447	0.0120	1.957E-04
-0.0098	0.0046	3.300E-02
-0.0104	0.0046	2.459E-02
-0.1977	0.0087	6.390E-115
0.1928	0.0086	2.860E-110
0.0040	0.0047	3.952E-01
-0.0051	0.0078	5.139E-01
0.0085	0.0062	1.695E-01
-0.0077	0.0042	6.902E-02
-0.0057	0.0043	1.869E-01
0.0010	0.0053	8.489E-01
-0.0005	0.0054	9.223E-01
-0.0399	0.0056	1.364E-12
-0.0303	0.0062	1.037E-06
-0.0311	0.0168	6.506E-02
0.0105	0.0045	1.930E-02
-0.0166	0.0181	3.579E-01
-0.0104	0.0047	2.623E-02
0.0103	0.0047	3.028E-02
-0.0355	0.0046	6.669E-15
-0.0351	0.0045	1.194E-14
0.0117	0.0047	1.360E-02
-0.0186	0.0047	6.733E-05
0.0195	0.0047	3.237E-05
0.0126	0.0042	2.441E-03
-0.0223	0.0044	4.164E-07
0.0722	0.0089	3.713E-16
-0.0006	0.0102	9.538E-01
-0.1138	0.0063	3.257E-72
-0.1144	0.0063	5.786E-74
0.0887	0.0065	3.614E-42
0.0148	0.0068	3.057E-02

0.0959	0.0109	1.681E-18
-0.0107	0.0047	2.406E-02
-0.0001	0.0051	9.836E-01
0.0179	0.0041	1.501E-05
-0.0111	0.0047	1.714E-02
-0.0903	0.0206	1.207E-05
0.0901	0.0206	1.227E-05
0.0127	0.0057	2.573E-02
0.0121	0.0055	2.740E-02
0.0396	0.0046	1.209E-17
-0.0387	0.0046	7.508E-17
0.0067	0.0072	3.505E-01
-0.0024	0.0043	5.681E-01
0.0004	0.0042	9.185E-01
-0.0128	0.0041	1.963E-03
-0.0071	0.0127	5.741E-01
-0.0020	0.0087	8.183E-01
-0.0087	0.0043	4.350E-02
-0.0274	0.0047	6.714E-09
0.0263	0.0046	1.228E-08
-0.0218	0.0046	1.977E-06
-0.0220	0.0046	1.539E-06
0.0042	0.0046	3.542E-01
-0.0082	0.0071	2.456E-01
0.0071	0.0098	4.729E-01
-0.0105	0.0046	2.191E-02
-0.0304	0.0087	5.074E-04
0.0108	0.0048	2.512E-02
0.0232	0.0077	2.713E-03
-0.0152	0.0072	3.594E-02
0.0337	0.0073	4.305E-06
-0.0127	0.0044	4.040E-03
0.0218	0.0046	2.321E-06
0.0431	0.0144	2.736E-03
0.0005	0.0043	9.118E-01
-0.0314	0.0092	6.835E-04
-0.0569	0.0118	1.572E-06
0.0128	0.0042	2.234E-03
-0.0163	0.0041	8.724E-05
-0.0179	0.0042	2.263E-05
0.0089	0.0049	6.974E-02
-0.0141	0.0058	1.548E-02
0.0105	0.0065	1.058E-01
0.0005	0.0060	9.276E-01
0.0045	0.0125	7.207E-01
0.0079	0.0089	3.740E-01
-0.0044	0.0062	4.763E-01
-0.0033	0.0046	4.706E-01

-0.0048	0.0047	3.076E-01
-0.0213	0.0049	1.545E-05
-0.0245	0.0053	3.416E-06
-0.0023	0.0111	8.368E-01
0.0117	0.0079	1.382E-01
0.0283	0.0041	8.855E-12
-0.0083	0.0046	7.088E-02
0.0076	0.0043	7.328E-02
-0.0029	0.0044	5.053E-01
0.0011	0.0047	8.207E-01
-0.0016	0.0042	7.014E-01
-0.0064	0.0048	1.840E-01
-0.0410	0.0135	2.344E-03
0.0091	0.0051	7.419E-02
0.0036	0.0098	7.142E-01
-0.0106	0.0043	1.355E-02
0.0038	0.0054	4.817E-01
-0.0049	0.0082	5.483E-01
0.0115	0.0071	1.070E-01
0.0005	0.0062	9.291E-01
-0.0021	0.0064	7.426E-01
0.0902	0.0041	2.990E-107
-0.0901	0.0041	6.960E-107
0.0085	0.0043	4.692E-02
-0.0086	0.0043	4.563E-02
0.0157	0.0043	2.645E-04
-0.0159	0.0042	1.334E-04
-0.0089	0.0048	6.467E-02
0.0003	0.0042	9.452E-01
-0.0004	0.0042	9.322E-01
-0.0497	0.0049	1.246E-24
-0.0107	0.0042	1.093E-02
-0.0009	0.0050	8.560E-01
0.0116	0.0078	1.402E-01
0.0176	0.0051	4.966E-04
-0.0161	0.0050	1.348E-03
0.0008	0.0044	8.592E-01
-0.0011	0.0046	8.077E-01
0.0181	0.0054	8.135E-04
-0.0022	0.0045	6.290E-01
0.0150	0.0046	1.088E-03
-0.0033	0.0049	4.950E-01
0.0193	0.0165	2.402E-01
0.0174	0.0065	7.554E-03
0.0224	0.0045	6.719E-07
-0.0160	0.0047	7.363E-04
0.0345	0.0074	3.633E-06
0.0116	0.0045	1.038E-02

-0.0416	0.0177	1.860E-02
0.0151	0.0073	3.895E-02
0.0138	0.0043	1.173E-03
-0.0168	0.0047	3.615E-04
0.0167	0.0047	3.608E-04
-0.0203	0.0062	1.090E-03
-0.0227	0.0167	1.729E-01
-0.0047	0.0041	2.520E-01
0.0114	0.0060	5.802E-02
-0.0028	0.0047	5.533E-01
-0.0058	0.0041	1.613E-01
0.0067	0.0044	1.258E-01
0.0367	0.0044	6.927E-17
0.0368	0.0044	7.682E-17
-0.0195	0.0118	9.798E-02
0.0113	0.0041	6.398E-03
0.0074	0.0046	1.092E-01
0.0080	0.0131	5.434E-01
0.0030	0.0045	4.949E-01
0.0169	0.0041	4.404E-05
0.0100	0.0068	1.408E-01
0.0182	0.0052	5.134E-04
0.0119	0.0049	1.495E-02
0.0119	0.0053	2.502E-02
-0.0084	0.0043	5.010E-02
-0.0071	0.0075	3.426E-01
-0.0074	0.0043	8.262E-02
-0.0423	0.0049	5.425E-18
-0.0425	0.0049	3.156E-18
-0.0359	0.0130	5.548E-03
-0.0349	0.0130	7.192E-03
0.0280	0.0042	3.101E-11
-0.0134	0.0042	1.542E-03
-0.0051	0.0045	2.502E-01
-0.0103	0.0054	5.473E-02
-0.0020	0.0043	6.413E-01
0.0383	0.0180	3.389E-02
0.0011	0.0042	8.016E-01
0.0361	0.0129	5.332E-03
0.0024	0.0044	5.855E-01
0.0062	0.0045	1.667E-01
0.0173	0.0046	1.538E-04
-0.0276	0.0050	4.008E-08
0.0206	0.0055	1.669E-04
-0.0240	0.0073	1.083E-03
0.0339	0.0113	2.710E-03
-0.0295	0.0163	6.950E-02
-0.0181	0.0081	2.611E-02

-0.0147	0.0047	1.838E-03
-0.0069	0.0045	1.298E-01
-0.0018	0.0042	6.691E-01
-0.0007	0.0046	8.781E-01
0.0009	0.0046	8.381E-01
-0.0036	0.0054	5.135E-01
0.0678	0.0159	1.937E-05
-0.0209	0.0042	6.078E-07
-0.0186	0.0046	4.764E-05
0.0089	0.0067	1.848E-01
-0.0919	0.0204	6.814E-06
-0.1005	0.0206	1.065E-06
0.0009	0.0055	8.737E-01
-0.0048	0.0048	3.114E-01
-0.0035	0.0043	4.199E-01
-0.0161	0.0249	5.180E-01
-0.0236	0.0049	1.424E-06
-0.0242	0.0049	7.724E-07
-0.0361	0.0058	6.636E-10
0.0323	0.0059	5.665E-08
-0.0386	0.0047	1.602E-16
0.0073	0.0045	1.028E-01
-0.0182	0.0053	5.393E-04
-0.0104	0.0081	1.954E-01
-0.0445	0.0046	8.244E-22
0.0520	0.0042	5.082E-36
0.0280	0.0051	3.392E-08
-0.0279	0.0051	4.585E-08
0.0123	0.0046	8.334E-03
0.0261	0.0057	4.442E-06
0.0285	0.0066	1.746E-05
0.0245	0.0071	5.980E-04
-0.0122	0.0048	1.056E-02
0.0628	0.0180	4.718E-04
-0.0565	0.0176	1.312E-03
-0.0044	0.0044	3.169E-01
0.0054	0.0042	1.930E-01
-0.0044	0.0042	2.948E-01
0.0247	0.0044	2.281E-08
-0.0250	0.0045	2.078E-08
0.0320	0.0080	6.182E-05
0.0367	0.0047	1.045E-14
-0.0378	0.0047	1.306E-15
0.0401	0.0079	4.120E-07
0.0900	0.0047	1.096E-82
0.0903	0.0047	2.601E-83
0.0140	0.0060	1.997E-02
-0.0075	0.0090	4.016E-01

-0.0072	0.0088	4.137E-01
-0.0190	0.0045	2.222E-05
-0.0008	0.0043	8.571E-01
-0.0079	0.0171	6.426E-01
-0.0108	0.0044	1.287E-02
0.0039	0.0042	3.501E-01
-0.0091	0.0055	9.480E-02
0.0000	0.0067	9.980E-01
0.0219	0.0042	2.329E-07
0.0218	0.0042	2.632E-07
-0.0571	0.0051	1.255E-28
0.0636	0.0050	1.359E-37
-0.0136	0.0046	3.438E-03
0.0289	0.0096	2.735E-03
0.0477	0.0045	1.523E-26
-0.0473	0.0045	2.741E-26
-0.0227	0.0209	2.776E-01
-0.0212	0.0047	5.512E-06
-0.0171	0.0041	3.434E-05
0.0173	0.0041	2.640E-05
0.0068	0.0120	5.701E-01
0.0417	0.0081	2.701E-07
-0.0155	0.0041	1.588E-04
0.0152	0.0041	2.186E-04
0.0022	0.0042	6.116E-01
-0.0035	0.0101	7.295E-01
0.0073	0.0045	1.083E-01
0.0024	0.0064	7.021E-01
-0.0521	0.0049	3.879E-26
0.0507	0.0049	2.258E-25
-0.0062	0.0042	1.352E-01
0.0052	0.0047	2.622E-01
-0.0141	0.0043	1.024E-03
0.0046	0.0042	2.683E-01
-0.0036	0.0064	5.770E-01
0.0150	0.0089	9.140E-02
0.0046	0.0050	3.596E-01
-0.0315	0.0078	5.450E-05
0.0048	0.0050	3.346E-01
-0.0056	0.0051	2.677E-01
0.0128	0.0045	4.710E-03
-0.0253	0.0058	1.432E-05
-0.0560	0.0065	9.443E-18
0.0504	0.0064	2.376E-15
-0.0264	0.0106	1.252E-02
0.0022	0.0077	7.795E-01
-0.0098	0.0082	2.351E-01
0.0270	0.0119	2.383E-02

0.0006	0.0046	8.949E-01
0.0097	0.0041	1.940E-02
-0.0089	0.0059	1.303E-01
0.0179	0.0043	2.847E-05
-0.0218	0.0043	2.785E-07
-0.0088	0.0102	3.842E-01
-0.0108	0.0045	1.577E-02
0.0003	0.0057	9.551E-01
0.0174	0.0049	4.053E-04
0.0359	0.0048	4.952E-14
0.0125	0.0043	3.851E-03
-0.0192	0.0053	3.048E-04
0.0586	0.0055	1.061E-26
-0.0193	0.0043	7.487E-06
-0.0200	0.0043	3.172E-06
-0.0197	0.0042	2.797E-06
-0.0253	0.0044	1.180E-08
-0.0427	0.0042	1.030E-24
0.0089	0.0086	2.997E-01
-0.0010	0.0062	8.660E-01
0.0002	0.0061	9.676E-01
-0.0215	0.0045	1.770E-06
0.0118	0.0055	3.315E-02
0.0108	0.0061	7.600E-02
0.0290	0.0042	4.833E-12
-0.0277	0.0043	7.607E-11
-0.0151	0.0059	1.094E-02
0.0030	0.0050	5.474E-01
-0.0062	0.0054	2.529E-01
0.0038	0.0056	4.987E-01
0.0136	0.0043	1.429E-03
-0.0134	0.0042	1.481E-03
-0.0198	0.0045	1.295E-05
0.0380	0.0093	4.077E-05
0.0423	0.0102	3.291E-05
-0.0190	0.0050	1.407E-04
-0.0017	0.0076	8.193E-01
-0.0191	0.0079	1.556E-02
-0.0188	0.0080	1.811E-02
-0.0155	0.0045	5.866E-04
0.0104	0.0076	1.691E-01
-0.0135	0.0046	3.614E-03
0.0201	0.0048	3.003E-05
-0.0144	0.0042	5.324E-04
0.0137	0.0047	3.247E-03
0.0085	0.0053	1.061E-01
-0.0037	0.0041	3.751E-01
0.0284	0.0152	6.104E-02

-0.0218	0.0145	1.330E-01
-0.0051	0.0047	2.773E-01
-0.0011	0.0043	7.929E-01
-0.0079	0.0046	8.221E-02
-0.0085	0.0042	4.491E-02
0.0001	0.0042	9.782E-01
0.0162	0.0100	1.043E-01
-0.0058	0.0053	2.719E-01
0.0052	0.0055	3.447E-01
0.0061	0.0043	1.542E-01
0.0087	0.0056	1.240E-01
-0.0114	0.0049	2.073E-02
-0.0124	0.0068	6.871E-02
-0.0090	0.0050	7.108E-02
0.0228	0.0042	6.622E-08
0.0225	0.0041	4.420E-08
0.0205	0.0043	1.797E-06
-0.0116	0.0042	5.620E-03
0.0148	0.0080	6.461E-02
-0.0131	0.0062	3.583E-02
-0.0053	0.0047	2.653E-01
0.0000	0.0055	9.996E-01
0.0068	0.0076	3.677E-01
-0.0139	0.0045	1.739E-03
-0.0823	0.0048	4.649E-65
-0.0552	0.0045	3.089E-34
-0.0450	0.0047	7.351E-22
0.0071	0.0043	9.777E-02
0.0138	0.0042	9.961E-04
0.0201	0.0051	7.167E-05
0.0146	0.0042	5.403E-04
-0.0183	0.0085	3.191E-02
0.0046	0.0147	7.530E-01
0.0066	0.0078	3.969E-01
-0.0260	0.0044	3.122E-09
0.0114	0.0122	3.490E-01
-0.0156	0.0046	6.919E-04
-0.0377	0.0053	1.358E-12
0.0344	0.0047	3.244E-13
0.0145	0.0041	4.271E-04
-0.0081	0.0049	1.016E-01
-0.0123	0.0043	4.516E-03
0.0329	0.0044	1.104E-13
-0.0099	0.0049	4.261E-02
-0.0061	0.0041	1.369E-01
-0.0112	0.0049	2.257E-02
0.0357	0.0042	1.237E-17
0.0144	0.0041	5.028E-04

0.0096	0.0045	3.226E-02
0.0084	0.0044	5.766E-02
-0.0014	0.0043	7.388E-01
0.0099	0.0048	3.855E-02
-0.0046	0.0056	4.110E-01
0.0098	0.0043	2.404E-02
-0.0098	0.0043	2.431E-02
-0.0142	0.0043	9.845E-04
0.0126	0.0064	4.892E-02
-0.0080	0.0044	6.818E-02
0.0152	0.0041	2.399E-04
0.0030	0.0042	4.776E-01
-0.0131	0.0073	7.378E-02
-0.0154	0.0120	1.991E-01
-0.0154	0.0068	2.270E-02
0.0191	0.0047	5.521E-05
-0.0327	0.0071	3.877E-06
-0.0302	0.0069	1.051E-05
0.0414	0.0090	4.831E-06
0.0675	0.0062	1.335E-27
-0.0083	0.0042	5.080E-02
-0.0838	0.0059	1.012E-45
0.1210	0.0045	1.300E-156
-0.1166	0.0049	1.100E-124
-0.0705	0.0046	1.957E-53
0.0713	0.0046	2.443E-55
0.0168	0.0044	1.531E-04
-0.0074	0.0060	2.224E-01
0.0172	0.0067	1.053E-02
0.0165	0.0078	3.390E-02
-0.0094	0.0070	1.777E-01
0.0058	0.0063	3.552E-01
-0.0103	0.0049	3.472E-02
0.0249	0.0049	4.395E-07
0.0084	0.0052	1.040E-01
-0.0041	0.0058	4.811E-01
-0.0101	0.0041	1.359E-02
0.0261	0.0052	5.424E-07
-0.0156	0.0043	2.461E-04
-0.0136	0.0074	6.731E-02
0.0038	0.0053	4.654E-01
-0.0035	0.0052	5.021E-01
0.0547	0.0059	2.937E-20
-0.4924	0.0088	6.82E-689
0.1108	0.0071	5.660E-55
-0.0447	0.0051	1.930E-18
-0.1492	0.0071	3.480E-99
0.0101	0.0080	2.073E-01

-0.0022	0.0076	7.728E-01
-0.0008	0.0045	8.513E-01
-0.0028	0.0049	5.661E-01
-0.0073	0.0048	1.296E-01
0.0044	0.0049	3.669E-01
-0.0078	0.0043	6.651E-02
-0.0077	0.0042	7.142E-02
-0.0052	0.0043	2.306E-01
0.0046	0.0065	4.809E-01
-0.0287	0.0044	4.126E-11
-0.0282	0.0043	9.311E-11
-0.0150	0.0082	6.731E-02
0.0156	0.0082	5.747E-02
-0.0303	0.0060	4.354E-07
0.0250	0.0054	3.075E-06
-0.0121	0.0047	9.945E-03
0.0256	0.0042	1.502E-09
0.0375	0.0052	8.554E-13
-0.0360	0.0047	3.012E-14
0.0191	0.0043	8.762E-06
-0.0297	0.0078	1.443E-04
-0.0086	0.0043	4.386E-02
0.0126	0.0042	2.893E-03
-0.0099	0.0045	2.845E-02
0.0114	0.0043	7.844E-03
0.0084	0.0046	6.480E-02
0.0249	0.0106	1.852E-02
0.0147	0.0044	7.976E-04
-0.0151	0.0041	2.656E-04
0.0130	0.0042	1.889E-03
0.0081	0.0047	8.576E-02
-0.0065	0.0046	1.592E-01
0.0364	0.0075	1.353E-06
-0.0020	0.0044	6.442E-01
-0.0087	0.0041	3.649E-02
0.0074	0.0042	8.066E-02
0.0103	0.0041	1.196E-02
0.0115	0.0043	6.955E-03
0.0039	0.0051	4.471E-01
0.0043	0.0042	3.047E-01
-0.0065	0.0042	1.225E-01
0.0089	0.0048	6.133E-02
-0.0091	0.0048	5.450E-02
0.0043	0.0054	4.206E-01
-0.0210	0.0052	5.218E-05
-0.0190	0.0080	1.711E-02
0.0093	0.0046	4.168E-02
-0.0327	0.0043	4.753E-14

-0.0329	0.0043	3.650E-14
-0.0136	0.0041	9.052E-04
0.0480	0.0058	1.730E-16
0.0417	0.0044	2.766E-21
-0.0428	0.0047	4.008E-20
0.0421	0.0068	5.849E-10
-0.0108	0.0130	4.056E-01
0.0263	0.0045	3.521E-09
-0.0566	0.0089	1.999E-10
-0.0247	0.0054	4.103E-06
-0.0274	0.0049	2.004E-08
0.0390	0.0054	3.970E-13
-0.0511	0.0119	1.747E-05
0.0509	0.0119	1.897E-05
0.1594	0.0085	2.619E-78
-0.1656	0.0086	9.779E-83
0.0154	0.0041	1.552E-04
-0.0085	0.0080	2.871E-01
-0.0053	0.0042	2.050E-01
0.0040	0.0069	5.646E-01
-0.0413	0.0056	1.320E-13
-0.0323	0.0062	1.520E-07
-0.0321	0.0167	5.383E-02
0.0101	0.0044	2.271E-02
-0.0436	0.0045	4.273E-22
-0.0433	0.0045	6.802E-22
0.0165	0.0041	5.391E-05
-0.0054	0.0075	4.720E-01
-0.0254	0.0043	2.799E-09
-0.0253	0.0043	3.094E-09
-0.0190	0.0105	7.004E-02
0.0350	0.0046	1.802E-14
-0.1206	0.0063	2.441E-82
-0.1210	0.0062	5.389E-84
0.0823	0.0065	3.272E-37
0.0169	0.0068	1.251E-02
0.0769	0.0109	1.383E-12
0.0654	0.0041	2.052E-57
0.0511	0.0043	2.582E-32
-0.0751	0.0203	2.220E-04
-0.0815	0.0205	6.800E-05
0.0135	0.0055	1.361E-02
-0.0342	0.0046	8.493E-14
0.0327	0.0046	8.456E-13
-0.0099	0.0061	1.038E-01
-0.0061	0.0042	1.486E-01
0.0055	0.0057	3.395E-01
-0.0041	0.0094	6.657E-01

-0.0038	0.0095	6.859E-01
0.0088	0.0041	3.280E-02
-0.0097	0.0041	1.958E-02
-0.0064	0.0043	1.377E-01
0.0100	0.0054	6.421E-02
0.0308	0.0046	1.235E-11
-0.0042	0.0054	4.416E-01
-0.0224	0.0045	7.498E-07
-0.0148	0.0070	3.444E-02
-0.0138	0.0098	1.602E-01
0.0138	0.0098	1.601E-01
-0.0145	0.0066	2.944E-02
-0.0253	0.0080	1.460E-03
-0.0125	0.0062	4.407E-02
0.0318	0.0073	1.217E-05
0.0201	0.0043	2.382E-06
-0.0114	0.0041	5.594E-03
-0.0250	0.0139	7.245E-02
-0.0324	0.0092	4.029E-04
-0.0513	0.0118	1.271E-05
-0.0019	0.0041	6.353E-01
0.0208	0.0048	1.758E-05
-0.0130	0.0058	2.467E-02
0.0128	0.0064	4.679E-02
0.0073	0.0075	3.250E-01
-0.0263	0.0052	4.788E-07
-0.0128	0.0130	3.262E-01
-0.0363	0.0041	1.948E-18
0.0418	0.0042	2.204E-23
0.0361	0.0042	3.871E-18
-0.0042	0.0048	3.758E-01
-0.0081	0.0042	5.222E-02
-0.0169	0.0065	9.044E-03
-0.0013	0.0046	7.768E-01
0.0057	0.0042	1.807E-01
0.0005	0.0098	9.584E-01
-0.0019	0.0049	6.960E-01
-0.0013	0.0082	8.741E-01
-0.0074	0.0063	2.373E-01
0.0841	0.0041	4.210E-95
-0.0838	0.0041	1.450E-94
-0.0090	0.0042	3.410E-02
-0.0139	0.0041	7.229E-04
-0.0009	0.0042	8.287E-01
-0.0579	0.0048	2.420E-33
0.0580	0.0048	2.137E-33
0.0187	0.0050	1.878E-04
-0.0208	0.0042	8.614E-07

0.0194	0.0053	2.806E-04
0.0120	0.0074	1.039E-01
0.0203	0.0045	7.831E-06
-0.0042	0.0049	3.908E-01
0.0241	0.0065	1.999E-04
0.0270	0.0074	2.498E-04
0.0305	0.0045	7.534E-12
-0.0211	0.0047	7.177E-06
-0.0380	0.0168	2.417E-02
0.0285	0.0129	2.636E-02
0.0276	0.0072	1.319E-04
0.0135	0.0046	3.751E-03
-0.0187	0.0062	2.627E-03
-0.0040	0.0047	3.900E-01
-0.0257	0.0111	2.023E-02
0.0404	0.0044	1.855E-20
0.0397	0.0044	1.569E-19
0.0130	0.0041	1.473E-03
-0.0132	0.0048	5.598E-03
0.0059	0.0044	1.814E-01
0.0235	0.0041	8.329E-09
0.0232	0.0052	7.003E-06
-0.0050	0.0065	4.374E-01
-0.0101	0.0042	1.738E-02
0.0264	0.0097	6.376E-03
0.0432	0.0049	7.904E-19
-0.0433	0.0048	3.533E-19
-0.0313	0.0128	1.471E-02
-0.0314	0.0129	1.477E-02
-0.0222	0.0042	1.072E-07
-0.0011	0.0043	8.060E-01
0.0439	0.0179	1.397E-02
0.0003	0.0042	9.394E-01
0.0331	0.0128	9.892E-03
0.0005	0.0044	9.128E-01
-0.0046	0.0067	4.944E-01
-0.0167	0.0045	1.993E-04
0.0070	0.0043	1.047E-01
-0.0314	0.0161	5.108E-02
0.0871	0.0158	3.252E-08
0.0195	0.0045	1.436E-05
-0.0885	0.0202	1.229E-05
-0.0981	0.0204	1.443E-06
-0.0174	0.0043	4.384E-05
-0.0273	0.0048	1.765E-08
-0.0435	0.0058	5.398E-14
0.0439	0.0046	1.141E-21
0.0400	0.0059	1.086E-11

-0.0118	0.0043	5.990E-03
-0.0089	0.0053	9.727E-02
0.0220	0.0130	9.041E-02
-0.0020	0.0041	6.205E-01
-0.0403	0.0046	9.256E-19
0.0676	0.0041	6.853E-61
-0.0360	0.0051	1.272E-12
0.0260	0.0056	3.912E-06
0.0277	0.0066	2.533E-05
0.0291	0.0071	3.759E-05
0.0244	0.0073	7.983E-04
0.0653	0.0182	3.243E-04
-0.0453	0.0164	5.607E-03
0.0125	0.0042	3.053E-03
0.0260	0.0044	2.814E-09
-0.0462	0.0045	7.111E-25
0.0588	0.0079	9.606E-14
0.0772	0.0047	1.010E-60
-0.0795	0.0047	1.543E-64
0.0152	0.0042	2.907E-04
-0.0072	0.0041	7.710E-02
0.0820	0.0046	3.028E-70
0.0824	0.0046	5.085E-71
-0.0233	0.0044	1.066E-07
-0.0231	0.0044	1.885E-07
-0.0219	0.0076	3.937E-03
0.0218	0.0098	2.638E-02
-0.0452	0.0051	7.122E-19
-0.0491	0.0084	5.519E-09
-0.0160	0.0046	4.746E-04
0.0119	0.0089	1.805E-01
0.0506	0.0044	3.789E-30
-0.0496	0.0044	2.661E-29
0.0503	0.0044	1.446E-30
0.0113	0.0041	6.118E-03
-0.0183	0.0041	7.336E-06
0.0180	0.0041	1.117E-05
-0.0262	0.0048	6.077E-08
0.0261	0.0048	6.636E-08
-0.0312	0.0050	3.118E-10
-0.0167	0.0042	6.577E-05
0.0357	0.0049	3.243E-13
-0.0350	0.0049	1.329E-12
-0.0357	0.0045	1.334E-15
-0.0251	0.0057	1.099E-05
-0.0645	0.0087	1.322E-13
0.0315	0.0063	4.910E-07
0.0211	0.0064	1.062E-03

-0.0111	0.0041	6.942E-03
-0.0258	0.0041	3.865E-10
0.0136	0.0049	5.074E-03
-0.0149	0.0045	8.150E-04
0.0287	0.0047	1.022E-09
0.0204	0.0044	2.895E-06
0.0309	0.0044	2.768E-12
0.0255	0.0118	3.035E-02
-0.0177	0.0044	5.215E-05
0.0163	0.0049	8.078E-04
0.0129	0.0043	2.503E-03
-0.0254	0.0044	8.278E-09
-0.0429	0.0041	1.936E-25
-0.0232	0.0044	1.733E-07
-0.0229	0.0045	3.098E-07
-0.0320	0.0045	7.330E-13
0.0277	0.0052	1.067E-07
0.0188	0.0041	4.867E-06
-0.0041	0.0047	3.839E-01
0.0141	0.0042	8.115E-04
0.0130	0.0041	1.744E-03
0.0108	0.0046	1.749E-02
0.0360	0.0092	8.752E-05
-0.0081	0.0063	2.004E-01
0.0186	0.0079	1.788E-02
-0.0090	0.0075	2.271E-01
-0.0184	0.0041	7.922E-06
-0.0204	0.0108	5.829E-02
-0.0367	0.0059	6.441E-10
0.0562	0.0050	8.133E-30
-0.0642	0.0041	1.337E-55
0.0222	0.0059	1.721E-04
-0.0870	0.0041	7.830E-99
-0.0819	0.0042	7.693E-85
-0.0948	0.0055	1.332E-67
0.0958	0.0054	3.822E-69
-0.0630	0.0070	1.387E-19
-0.0642	0.0054	1.895E-32
-0.0179	0.0056	1.415E-03
0.0770	0.0047	9.802E-60
0.0576	0.0045	9.179E-38
0.0202	0.0050	5.896E-05
0.0275	0.0043	1.291E-10
-0.0278	0.0043	1.607E-10
-0.0164	0.0046	4.019E-04
0.0064	0.0049	1.957E-01
0.0139	0.0042	9.140E-04
-0.0118	0.0043	6.138E-03

0.0348	0.0044	2.171E-15
-0.0053	0.0041	1.958E-01
0.0262	0.0041	2.050E-10
0.0117	0.0041	4.272E-03
0.0094	0.0044	3.236E-02
-0.0010	0.0042	8.063E-01
-0.0122	0.0043	4.442E-03
-0.0633	0.0042	6.759E-52
-0.0300	0.0046	7.087E-11
-0.0500	0.0055	1.801E-19
-0.0169	0.0043	9.683E-05
0.0129	0.0041	1.641E-03
-0.0030	0.0073	6.777E-01
-0.0176	0.0067	8.445E-03
0.0184	0.0047	8.643E-05
-0.0280	0.0070	6.355E-05
0.0680	0.0104	6.693E-11
-0.0060	0.0042	1.499E-01
0.0792	0.0059	2.546E-41
-0.0998	0.0049	2.290E-93
0.0907	0.0045	7.229E-89
0.0896	0.0045	9.438E-88
-0.0102	0.0067	1.251E-01
0.0089	0.0067	1.827E-01
-0.0217	0.0077	5.048E-03
0.0268	0.0069	9.402E-05
-0.0036	0.0133	7.879E-01
0.0133	0.0063	3.414E-02
-0.0067	0.0041	9.953E-02
0.0237	0.0052	4.302E-06
-0.0163	0.0074	2.722E-02
-0.0028	0.0052	5.908E-01
0.0518	0.0059	1.038E-18
-0.3423	0.0087	9.74E-339
-0.0430	0.0051	1.827E-17
0.3562	0.0084	6.54E-396
-0.1350	0.0070	1.044E-83
-0.2562	0.0074	1.300E-263
-0.1180	0.0067	2.083E-70
-0.0502	0.0058	2.910E-18
-0.0077	0.0043	7.212E-02
-0.0180	0.0043	3.049E-05
-0.0042	0.0066	5.212E-01
-0.0352	0.0059	3.282E-09
-0.0345	0.0059	5.836E-09
-0.0100	0.0047	3.178E-02
0.0238	0.0043	2.274E-08
0.0377	0.0052	3.494E-13

-0.0387	0.0052	8.094E-14
-0.0136	0.0041	9.195E-04
0.0109	0.0041	7.887E-03
0.0138	0.0041	8.584E-04
0.0067	0.0043	1.156E-01
-0.0020	0.0071	7.806E-01
0.0205	0.0046	7.843E-06
0.0084	0.0080	2.898E-01
-0.0161	0.0045	3.711E-04
0.0295	0.0066	7.899E-06
0.0085	0.0043	4.859E-02
-0.0100	0.0120	4.030E-01
-0.0389	0.0066	4.166E-09
0.0390	0.0066	3.963E-09
0.0112	0.0042	8.281E-03
0.0602	0.0049	9.141E-35
-0.0788	0.0054	1.459E-48
-0.0627	0.0149	2.700E-05
0.0007	0.0044	8.659E-01
0.0083	0.0045	6.636E-02
-0.0123	0.0042	3.499E-03
0.0119	0.0094	2.041E-01
-0.0060	0.0105	5.649E-01
0.0116	0.0057	4.098E-02
0.0185	0.0041	6.290E-06
0.0154	0.0043	3.004E-04
0.0063	0.0043	1.406E-01
-0.0073	0.0070	2.932E-01
-0.0058	0.0042	1.700E-01
-0.0099	0.0133	4.585E-01
-0.0125	0.0041	2.158E-03
0.0043	0.0066	5.194E-01
-0.0033	0.0043	4.368E-01
-0.0065	0.0042	1.229E-01
-0.0082	0.0042	5.291E-02
-0.0054	0.0093	5.619E-01
-0.0063	0.0042	1.365E-01
0.0122	0.0044	5.429E-03
-0.0150	0.0068	2.753E-02
-0.0134	0.0045	2.533E-03
0.0070	0.0046	1.277E-01
-0.0300	0.0055	5.558E-08
0.0189	0.0047	5.570E-05
0.0047	0.0091	6.040E-01
0.0043	0.0047	3.657E-01
-0.0099	0.0048	4.124E-02
-0.0088	0.0047	5.820E-02
0.0175	0.0044	6.432E-05

0.0605	0.0076	2.070E-15
-0.0134	0.0047	3.895E-03
0.0634	0.0088	4.962E-13
0.0252	0.0063	6.394E-05
0.0067	0.0068	3.251E-01
-0.0017	0.0044	6.913E-01
0.0024	0.0045	5.962E-01
-0.0069	0.0062	2.661E-01
-0.0089	0.0047	5.737E-02
0.1288	0.0041	5.560E-218
0.0823	0.0043	2.946E-81
0.0533	0.0045	6.929E-33
0.0031	0.0045	4.962E-01
-0.0167	0.0042	7.361E-05
0.0040	0.0051	4.350E-01
0.0003	0.0041	9.335E-01
-0.0090	0.0043	3.586E-02
-0.0100	0.0043	2.135E-02
0.0159	0.0043	2.412E-04
0.0173	0.0043	6.716E-05
0.0106	0.0045	1.905E-02
0.0032	0.0042	4.419E-01
0.0071	0.0041	8.286E-02
-0.0247	0.0045	3.415E-08
-0.0027	0.0046	5.611E-01
0.0064	0.0042	1.288E-01
-0.0257	0.0075	5.514E-04
-0.0199	0.0068	3.424E-03
0.0349	0.0243	1.510E-01
-0.0052	0.0045	2.408E-01
-0.0050	0.0047	2.841E-01
-0.0040	0.0046	3.795E-01
-0.0389	0.0080	1.170E-06
-0.0041	0.0042	3.245E-01
0.0109	0.0088	2.188E-01
0.0128	0.0082	1.203E-01
0.0039	0.0045	3.818E-01
-0.0093	0.0059	1.194E-01
0.0015	0.0076	8.484E-01
-0.0062	0.0041	1.321E-01
-0.0053	0.0044	2.235E-01
-0.0174	0.0063	5.735E-03
-0.0384	0.0114	7.494E-04
0.0241	0.0058	3.144E-05
-0.0142	0.0043	1.031E-03
-0.0035	0.0053	5.085E-01
0.0114	0.0043	8.150E-03
0.0068	0.0041	9.790E-02

0.0009	0.0071	8.958E-01
0.0031	0.0047	5.151E-01
-0.0066	0.0041	1.066E-01
0.0073	0.0111	5.100E-01
-0.0178	0.0136	1.901E-01
0.0081	0.0124	5.154E-01
-0.0238	0.0062	1.356E-04
-0.0213	0.0060	4.065E-04
-0.0084	0.0132	5.233E-01
0.0100	0.0041	1.507E-02
0.0055	0.0042	1.928E-01
0.0138	0.0049	4.561E-03
0.0000	0.0042	9.915E-01
0.0053	0.0059	3.687E-01
0.0085	0.0057	1.382E-01
0.0021	0.0096	8.243E-01
-0.0057	0.0046	2.192E-01
-0.0060	0.0047	2.059E-01
-0.0195	0.0057	5.593E-04
0.0075	0.0041	7.208E-02
0.0527	0.0041	1.327E-37
0.0532	0.0041	2.710E-38
0.0096	0.0041	1.914E-02
-0.0161	0.0144	2.640E-01
-0.0109	0.0071	1.277E-01
0.0047	0.0047	3.187E-01
-0.0084	0.0064	1.904E-01
-0.0095	0.0042	2.354E-02
0.0032	0.0059	5.874E-01
0.0032	0.0059	5.873E-01
0.0183	0.0068	6.790E-03
-0.0049	0.0044	2.610E-01
-0.0066	0.0042	1.159E-01
0.0021	0.0042	6.164E-01
0.0362	0.0043	2.233E-17
0.0407	0.0044	2.133E-20
-0.0022	0.0080	7.817E-01
0.0105	0.0045	1.910E-02
0.0095	0.0044	2.957E-02
-0.0033	0.0051	5.204E-01
0.0005	0.0049	9.158E-01
-0.0057	0.0061	3.552E-01
0.0094	0.0046	4.093E-02
0.0129	0.0047	6.106E-03
-0.0084	0.0043	5.203E-02
-0.0094	0.0045	3.491E-02
0.0079	0.0064	2.189E-01
0.0023	0.0042	5.887E-01

-0.0076	0.0044	8.520E-02
-0.0117	0.0041	4.018E-03
0.0120	0.0041	3.201E-03
0.0227	0.0097	1.961E-02
-0.0160	0.0071	2.402E-02
-0.0057	0.0044	2.025E-01
-0.0151	0.0048	1.692E-03
-0.0191	0.0044	1.557E-05
-0.0206	0.0079	9.413E-03
-0.0041	0.0041	3.128E-01
-0.0073	0.0043	8.792E-02
0.0081	0.0043	6.080E-02
-0.0119	0.0079	1.309E-01
0.0177	0.0060	3.227E-03
0.0051	0.0052	3.284E-01
0.0106	0.0063	9.254E-02
0.0109	0.0063	8.487E-02
0.0091	0.0077	2.342E-01
-0.0030	0.0062	6.238E-01
0.0088	0.0041	3.260E-02
0.0044	0.0048	3.557E-01
0.0404	0.0048	5.052E-17
0.0402	0.0048	7.184E-17
0.0176	0.0047	1.985E-04
0.0061	0.0081	4.576E-01
-0.0174	0.0121	1.521E-01
0.0022	0.0050	6.678E-01
-0.0032	0.0085	7.053E-01
0.0011	0.0057	8.450E-01
-0.0270	0.0054	6.065E-07
0.0187	0.0100	6.091E-02
0.0139	0.0122	2.517E-01
0.0200	0.0042	1.812E-06
0.0371	0.0046	1.472E-15
0.0040	0.0056	4.692E-01
0.0181	0.0041	9.264E-06
0.0294	0.0046	1.118E-10
0.0072	0.0138	5.998E-01
0.0413	0.0065	2.027E-10
0.0105	0.0041	1.050E-02
0.0179	0.0058	2.028E-03
0.0126	0.0074	8.826E-02
-0.0262	0.0048	5.148E-08
0.0453	0.0055	1.199E-16
0.1301	0.0092	2.346E-45
0.0305	0.0128	1.715E-02
0.0040	0.0045	3.763E-01
0.0040	0.0041	3.319E-01

0.0236	0.0043	4.369E-08
0.0340	0.0048	1.759E-12
-0.0316	0.0070	6.582E-06
-0.0277	0.0056	6.412E-07
-0.0067	0.0045	1.348E-01
0.0048	0.0076	5.274E-01
-0.0027	0.0042	5.262E-01
-0.0151	0.0055	5.888E-03
-0.0216	0.0051	2.538E-05
-0.0030	0.0196	8.784E-01
0.0060	0.0043	1.573E-01
0.0122	0.0042	3.790E-03
-0.0108	0.0041	8.312E-03
0.0012	0.0056	8.273E-01
0.0040	0.0049	4.100E-01
0.0269	0.0046	5.460E-09
0.0269	0.0046	6.836E-09
0.0084	0.0041	4.089E-02
-0.0076	0.0044	8.372E-02
0.0046	0.0067	4.911E-01
0.0034	0.0041	4.043E-01
-0.0109	0.0131	4.076E-01
-0.0084	0.0074	2.592E-01
-0.0048	0.0067	4.768E-01
-0.0055	0.0087	5.290E-01
-0.0138	0.0116	2.344E-01
-0.0016	0.0043	7.111E-01
0.0079	0.0041	5.542E-02
-0.0121	0.0043	4.764E-03
0.0107	0.0041	8.966E-03
-0.0046	0.0043	2.772E-01
0.0270	0.0097	5.344E-03
-0.0099	0.0048	4.198E-02
-0.0191	0.0096	4.757E-02
-0.0135	0.0041	1.068E-03
0.0126	0.0129	3.264E-01
-0.0200	0.0043	3.143E-06
-0.0118	0.0044	7.192E-03
-0.0162	0.0168	3.358E-01
0.0099	0.0129	4.395E-01
0.0066	0.0045	1.410E-01
0.0141	0.0045	1.619E-03
-0.0155	0.0041	1.447E-04
0.0124	0.0041	2.734E-03
-0.0178	0.0067	7.745E-03
-0.0230	0.0060	1.317E-04
0.1255	0.0067	3.246E-77
0.1270	0.0067	1.459E-79

0.0005	0.0041	9.052E-01
0.0037	0.0043	3.870E-01
0.0053	0.0043	2.138E-01
0.0244	0.0130	6.181E-02
0.0108	0.0042	9.959E-03
-0.0074	0.0042	7.730E-02
0.0134	0.0070	5.575E-02
0.0205	0.0045	5.125E-06
-0.0209	0.0043	1.032E-06
0.0163	0.0049	8.324E-04
0.0119	0.0056	3.479E-02
-0.0146	0.0055	8.427E-03
0.0013	0.0042	7.521E-01
-0.0088	0.0061	1.502E-01
0.0041	0.0047	3.805E-01
0.0831	0.0204	4.592E-05
0.0794	0.0204	1.022E-04
-0.0011	0.0044	8.080E-01
-0.0014	0.0082	8.650E-01
0.0010	0.0078	8.977E-01
0.0135	0.0174	4.377E-01
-0.0008	0.0045	8.587E-01
0.0038	0.0077	6.197E-01
0.0123	0.0083	1.372E-01
-0.0122	0.0044	5.000E-03
0.0010	0.0044	8.219E-01
0.0059	0.0045	1.875E-01
-0.0193	0.0048	5.185E-05
-0.0304	0.0043	1.003E-12
-0.0277	0.0086	1.348E-03
-0.0525	0.0062	2.424E-17
0.1682	0.0069	1.160E-130
0.1718	0.0061	1.740E-172
-0.1232	0.0050	1.750E-134
-0.0991	0.0047	2.760E-97
-0.0209	0.0051	4.707E-05
0.0061	0.0051	2.300E-01
0.0118	0.0050	1.743E-02
0.0131	0.0045	3.636E-03
0.0180	0.0057	1.689E-03
-0.0158	0.0194	4.139E-01
0.0265	0.0064	3.864E-05
-0.0212	0.0058	2.579E-04
-0.0202	0.0047	2.084E-05
-0.0239	0.0046	1.669E-07
0.0016	0.0042	7.079E-01
-0.0071	0.0049	1.527E-01
0.0724	0.0041	1.701E-69

-0.0625	0.0051	7.422E-35
0.0210	0.0046	5.004E-06
0.0051	0.0083	5.409E-01
-0.0067	0.0054	2.137E-01
-0.0087	0.0042	3.695E-02
0.0150	0.0048	1.837E-03
0.0264	0.0073	2.877E-04
-0.0110	0.0047	1.988E-02
-0.0107	0.0047	2.342E-02
-0.0048	0.0041	2.476E-01
0.0039	0.0062	5.226E-01
0.0030	0.0049	5.351E-01
-0.0069	0.0045	1.272E-01
0.0108	0.0130	4.062E-01
-0.0126	0.0046	6.398E-03
-0.0008	0.0041	8.514E-01
-0.0214	0.0047	5.046E-06
0.0216	0.0052	2.896E-05
0.0240	0.0049	8.424E-07
0.0078	0.0042	6.085E-02
0.0070	0.0042	9.811E-02
0.0096	0.0046	3.907E-02
-0.0043	0.0092	6.384E-01
-0.0308	0.0073	2.270E-05
0.0307	0.0073	2.254E-05
-0.0096	0.0044	2.964E-02
-0.0062	0.0044	1.586E-01
0.0365	0.0042	3.287E-18
-0.0372	0.0042	7.562E-19
0.0080	0.0041	5.476E-02
-0.0001	0.0041	9.887E-01
-0.0311	0.0050	5.937E-10
0.0296	0.0049	1.094E-09
-0.0126	0.0050	1.127E-02
-0.0134	0.0047	4.054E-03
0.0181	0.0123	1.429E-01
0.0080	0.0049	1.032E-01
-0.0402	0.0046	1.955E-18
-0.0405	0.0046	1.142E-18
-0.0045	0.0047	3.387E-01
-0.0197	0.0044	8.379E-06
0.0196	0.0044	8.823E-06
-0.0114	0.0045	1.151E-02
0.0247	0.0050	9.400E-07
-0.0046	0.0042	2.787E-01
-0.0256	0.0041	5.804E-10
-0.0025	0.0043	5.569E-01
0.0182	0.0044	3.367E-05

-0.0095	0.0113	4.003E-01
-0.0181	0.0049	2.114E-04
-0.0194	0.0076	1.125E-02
0.0004	0.0041	9.151E-01
-0.0212	0.0046	3.629E-06
0.0077	0.0076	3.102E-01
-0.0113	0.0047	1.631E-02
-0.0541	0.0048	5.460E-29
0.0535	0.0048	7.075E-29
0.0047	0.0042	2.681E-01
-0.0075	0.0090	4.011E-01
0.0100	0.0045	2.481E-02
-0.0182	0.0041	7.667E-06
0.0108	0.0046	2.015E-02
0.0132	0.0043	2.338E-03
0.0142	0.0053	7.592E-03
0.0044	0.0043	3.109E-01
-0.0110	0.0042	8.660E-03
-0.0009	0.0049	8.555E-01
0.0036	0.0041	3.842E-01
0.1433	0.0065	1.870E-106
-0.2071	0.0049	1.13E-389
0.3800	0.0075	1.27E-557
-0.3008	0.0044	1.90E-1025
-0.1781	0.0057	6.050E-216
0.0355	0.0042	5.967E-17
-0.0974	0.0093	1.180E-25
0.0396	0.0055	5.232E-13
-0.0739	0.0041	1.120E-72
-0.0101	0.0072	1.630E-01
0.0202	0.0059	6.727E-04
-0.0234	0.0058	5.763E-05
-0.0110	0.0056	5.201E-02
-0.0050	0.0091	5.834E-01
-0.0124	0.0047	8.191E-03
-0.0123	0.0047	8.838E-03
0.0077	0.0041	6.367E-02
-0.0120	0.0113	2.868E-01
0.0143	0.0064	2.642E-02
0.0010	0.0042	8.088E-01
0.0143	0.0066	3.204E-02
-0.0219	0.0064	6.787E-04
-0.0268	0.0070	1.149E-04
0.0002	0.0167	9.890E-01
-0.0102	0.0057	7.503E-02
-0.0103	0.0046	2.544E-02
0.0195	0.0041	2.436E-06
-0.0019	0.0045	6.665E-01

-0.0177	0.0055	1.188E-03
-0.0168	0.0043	9.974E-05
0.0165	0.0043	1.451E-04
-0.0242	0.0046	1.555E-07
0.0072	0.0041	7.895E-02
0.0092	0.0045	4.113E-02
0.0066	0.0065	3.069E-01
-0.0056	0.0066	3.890E-01
-0.0134	0.0041	1.200E-03
0.0007	0.0061	9.127E-01
0.0093	0.0044	3.398E-02
-0.0080	0.0044	6.870E-02
0.0097	0.0047	3.767E-02
0.0168	0.0047	3.680E-04
0.0126	0.0051	1.287E-02
-0.0118	0.0048	1.405E-02
0.0154	0.0076	4.310E-02
0.0082	0.0046	7.329E-02
-0.0237	0.0043	5.056E-08
0.0248	0.0043	1.128E-08
0.0019	0.0048	6.912E-01
0.0066	0.0042	1.160E-01
-0.0015	0.0042	7.152E-01
-0.0125	0.0046	6.987E-03
-0.0259	0.0049	1.091E-07
-0.0081	0.0052	1.163E-01
0.0090	0.0041	2.623E-02
0.0193	0.0045	2.197E-05
0.0196	0.0049	7.425E-05
0.0258	0.0079	1.011E-03
-0.0249	0.0078	1.403E-03
0.0058	0.0051	2.477E-01
0.0140	0.0184	4.459E-01
0.0065	0.0042	1.247E-01
0.0024	0.0062	7.026E-01
-0.0093	0.0046	4.534E-02
-0.0111	0.0071	1.173E-01
0.0070	0.0041	8.320E-02
-0.0086	0.0060	1.533E-01
-0.0111	0.0055	4.489E-02
0.0066	0.0059	2.666E-01
-0.0167	0.0103	1.030E-01
0.0061	0.0042	1.404E-01
-0.0079	0.0060	1.821E-01
0.0571	0.0150	1.375E-04
-0.0530	0.0041	1.998E-38
0.0493	0.0041	1.770E-33
0.0089	0.0059	1.346E-01

-0.0642	0.0041	1.631E-54
-0.0372	0.0043	4.057E-18
-0.0140	0.0077	6.936E-02
0.0150	0.0049	2.220E-03
-0.0152	0.0049	1.767E-03
-0.0280	0.0124	2.404E-02
0.0019	0.0044	6.709E-01
0.0035	0.0044	4.165E-01
-0.0136	0.0041	8.739E-04
0.0124	0.0043	4.173E-03
0.0078	0.0046	8.484E-02
-0.0125	0.0049	1.012E-02
-0.0016	0.0042	7.047E-01
0.0111	0.0041	7.166E-03
-0.0095	0.0042	2.255E-02
0.0246	0.0041	3.007E-09
0.0125	0.0042	2.843E-03
-0.0153	0.0061	1.266E-02
-0.0071	0.0047	1.274E-01
0.0178	0.0068	9.352E-03
-0.0028	0.0046	5.447E-01
-0.0056	0.0041	1.751E-01
-0.0176	0.0041	1.813E-05
0.0079	0.0043	6.587E-02
-0.0040	0.0077	6.059E-01
-0.0026	0.0076	7.320E-01
0.0014	0.0056	8.082E-01
-0.0411	0.0055	4.922E-14
0.0431	0.0054	2.198E-15
0.0340	0.0054	3.482E-10
0.0126	0.0124	3.099E-01
-0.0159	0.0111	1.519E-01
-0.0146	0.0111	1.858E-01
0.0019	0.0060	7.551E-01
-0.0149	0.0078	5.433E-02
-0.0048	0.0062	4.341E-01
0.0231	0.0045	2.717E-07
0.0251	0.0047	7.590E-08
-0.0067	0.0045	1.367E-01
0.0305	0.0042	5.267E-13
0.0214	0.0049	1.303E-05
-0.0138	0.0072	5.426E-02
0.0056	0.0065	3.865E-01
0.0001	0.0042	9.774E-01
-0.0016	0.0049	7.417E-01
-0.0017	0.0100	8.622E-01
-0.0070	0.0107	5.153E-01
-0.0294	0.0056	1.828E-07

0.0064	0.0048	1.796E-01
0.0132	0.0041	1.304E-03
-0.0110	0.0046	1.600E-02
-0.0051	0.0045	2.520E-01
-0.0157	0.0047	8.358E-04
0.0072	0.0041	7.814E-02
-0.0073	0.0041	7.846E-02
-0.0003	0.0049	9.480E-01
-0.0056	0.0056	3.169E-01
0.0038	0.0044	3.865E-01
-0.0064	0.0051	2.112E-01
-0.0063	0.0046	1.690E-01
-0.0087	0.0042	3.778E-02
0.0059	0.0072	4.132E-01
0.0069	0.0042	9.977E-02
-0.0094	0.0041	2.120E-02
0.0267	0.0043	6.152E-10
-0.0283	0.0043	6.652E-11
0.0161	0.0044	2.411E-04
0.0147	0.0044	9.183E-04
-0.0046	0.0091	6.103E-01
-0.0042	0.0042	3.125E-01
0.0038	0.0042	3.692E-01
0.0249	0.0041	1.573E-09
-0.0252	0.0041	1.104E-09
0.0196	0.0104	5.867E-02
-0.0147	0.0049	2.761E-03
-0.0311	0.0107	3.788E-03
0.0046	0.0074	5.299E-01
0.0098	0.0042	1.951E-02
-0.0097	0.0042	2.053E-02
0.0063	0.0043	1.396E-01
-0.0156	0.0084	6.302E-02
-0.0157	0.0049	1.388E-03
0.0127	0.0041	1.955E-03
-0.0509	0.0110	4.083E-06
-0.0511	0.0110	3.199E-06
-0.0024	0.0080	7.664E-01
0.0003	0.0042	9.424E-01
0.0066	0.0043	1.227E-01
0.0081	0.0042	5.785E-02
-0.0426	0.0157	6.651E-03
-0.0351	0.0045	1.278E-14
-0.0977	0.0077	1.475E-36
-0.0779	0.0158	8.546E-07
0.0064	0.0063	3.062E-01
-0.0135	0.0043	1.480E-03
-0.0020	0.0107	8.528E-01

0.0196	0.0041	1.940E-06
-0.0199	0.0041	1.138E-06
-0.0491	0.0129	1.426E-04
0.0030	0.0043	4.783E-01
0.0781	0.0059	2.158E-40
0.0676	0.0043	4.321E-56
0.1359	0.0055	1.130E-135
-0.0033	0.0041	4.224E-01
-0.0070	0.0044	1.090E-01
0.0070	0.0041	9.038E-02
-0.0114	0.0106	2.842E-01
0.0698	0.0121	8.132E-09
-0.0341	0.0061	1.896E-08
0.0295	0.0067	9.369E-06
0.0079	0.0043	6.570E-02
-0.0249	0.0156	1.102E-01
0.0288	0.0059	1.316E-06
-0.0064	0.0051	2.056E-01
0.0050	0.0043	2.400E-01
0.0188	0.0043	1.492E-05
0.0931	0.0084	1.178E-28
-0.0336	0.0043	3.287E-15
-0.0984	0.0181	5.575E-08
0.0929	0.0084	9.886E-29
0.0134	0.0089	1.332E-01
-0.0050	0.0042	2.293E-01
-0.0014	0.0047	7.712E-01
-0.0019	0.0041	6.505E-01
-0.0177	0.0045	8.178E-05
-0.0167	0.0045	2.124E-04
0.0130	0.0050	8.945E-03
0.0091	0.0059	1.225E-01
0.0050	0.0042	2.245E-01
-0.0101	0.0048	3.691E-02
-0.0100	0.0049	4.159E-02
-0.0092	0.0080	2.462E-01
0.0194	0.0043	8.009E-06
-0.0069	0.0041	8.898E-02
0.0204	0.0047	1.565E-05
0.0234	0.0043	4.680E-08
0.0185	0.0042	9.140E-06
-0.0056	0.0059	3.423E-01
-0.0011	0.0055	8.395E-01
-0.0076	0.0055	1.675E-01
-0.0245	0.0047	1.965E-07
0.0261	0.0046	1.171E-08
0.0306	0.0053	6.229E-09
-0.0359	0.0046	6.980E-15

0.0229	0.0043	1.346E-07
-0.0275	0.0046	2.683E-09
0.0278	0.0045	4.612E-10
0.0536	0.0060	4.523E-19
-0.0206	0.0044	2.815E-06
0.0147	0.0059	1.341E-02
0.0251	0.0046	4.438E-08
0.0171	0.0046	2.003E-04
-0.0290	0.0047	8.831E-10
-0.0249	0.0044	1.221E-08
-0.0016	0.0051	7.578E-01
-0.0537	0.0048	4.333E-29
0.0391	0.0044	1.090E-18
-0.0538	0.0088	8.356E-10
-0.0426	0.0047	8.820E-20
-0.0314	0.0163	5.342E-02
0.0403	0.0054	7.592E-14
-0.0667	0.0057	1.273E-31
-0.0014	0.0044	7.514E-01
-0.0031	0.0161	8.495E-01
0.0228	0.0044	1.985E-07
0.0216	0.0061	3.628E-04
0.0831	0.0043	2.157E-81
0.0043	0.0048	3.666E-01
0.0078	0.0052	1.315E-01
-0.0030	0.0049	5.423E-01
0.0060	0.0046	1.886E-01
0.0437	0.0046	1.428E-21
-0.0441	0.0050	9.349E-19
0.0124	0.0070	7.566E-02
0.0607	0.0143	2.151E-05
0.0333	0.0043	1.049E-14
0.0556	0.0047	4.143E-32
0.0329	0.0043	3.753E-14
-0.0131	0.0062	3.345E-02
0.0359	0.0100	3.347E-04
0.0220	0.0045	7.607E-07
0.0197	0.0044	8.679E-06
-0.0339	0.0047	6.438E-13
0.0373	0.0043	5.764E-18
-0.0003	0.0067	9.621E-01
0.0027	0.0051	5.974E-01
0.0054	0.0043	2.049E-01
-0.0108	0.0043	1.158E-02
0.0130	0.0043	2.490E-03
0.0241	0.0049	8.293E-07
-0.0415	0.0090	3.970E-06
0.0288	0.0047	7.845E-10

0.0547	0.0054	4.223E-24
-0.0667	0.0057	1.493E-31
-0.0001	0.0045	9.911E-01
0.0004	0.0043	9.331E-01
-0.0103	0.0055	6.116E-02
-0.0150	0.0138	2.769E-01
-0.0039	0.0050	4.442E-01
0.0290	0.0043	2.451E-11
-0.0199	0.0050	6.657E-05
0.0836	0.0043	9.939E-83
-0.0741	0.0057	4.747E-39
-0.0090	0.0045	4.673E-02
0.0197	0.0044	8.126E-06
0.0169	0.0057	3.118E-03
0.0158	0.0045	4.551E-04
0.0281	0.0066	2.298E-05
0.0185	0.0044	2.967E-05
0.0333	0.0045	2.368E-13
0.0659	0.0044	5.569E-50
-0.0035	0.0073	6.335E-01
0.0353	0.0048	2.789E-13
0.0030	0.0045	5.005E-01
0.0324	0.0047	3.525E-12
0.0360	0.0046	4.779E-15
-0.0378	0.0051	1.804E-13
-0.0338	0.0045	1.001E-13
0.0662	0.0133	6.970E-07
-0.0376	0.0045	4.107E-17
0.0253	0.0045	1.356E-08
0.0900	0.0045	2.950E-90
0.0208	0.0051	3.814E-05
0.0446	0.0065	7.285E-12
-0.0809	0.0088	6.141E-20
-0.0322	0.0050	1.254E-10
-0.0233	0.0044	1.706E-07
0.0237	0.0044	8.951E-08
0.0040	0.0051	4.245E-01
-0.0129	0.0184	4.838E-01
-0.0012	0.0050	8.135E-01
-0.0477	0.0049	2.841E-22
-0.0817	0.0072	1.004E-29
-0.0218	0.0045	1.016E-06
-0.0116	0.0067	8.190E-02
-0.0496	0.0085	5.833E-09
-0.0664	0.0054	2.706E-34
-0.0525	0.0053	3.992E-23
0.0013	0.0045	7.653E-01
-0.0034	0.0051	5.110E-01

0.0399	0.0049	4.877E-16
0.0120	0.0055	2.752E-02
0.0864	0.0071	3.796E-34
-0.1165	0.0059	6.484E-88
-0.1073	0.0044	1.370E-129
-0.1066	0.0044	2.720E-128
0.0010	0.0060	8.712E-01
0.0034	0.0145	8.145E-01
0.0249	0.0045	4.065E-08
-0.0751	0.0052	1.575E-47
-0.0145	0.0045	1.416E-03
0.0461	0.0078	3.543E-09
0.0449	0.0078	9.292E-09
0.0223	0.0045	5.962E-07
0.0297	0.0045	4.468E-11
0.0196	0.0060	1.116E-03
-0.0104	0.0046	2.236E-02
0.0776	0.0059	4.344E-40
0.0044	0.0050	3.786E-01
0.0191	0.0057	7.129E-04
-0.0468	0.0143	1.064E-03
-0.0316	0.0045	1.663E-12
0.0204	0.0052	9.366E-05
-0.2154	0.0046	3.74E-477
-0.0122	0.0050	1.523E-02
-0.0162	0.0044	2.463E-04
-0.0127	0.0084	1.284E-01
-0.0219	0.0044	7.252E-07
-0.0128	0.0048	7.544E-03
0.0188	0.0054	5.414E-04
-0.0721	0.0043	9.781E-64
-0.0716	0.0043	5.096E-63
0.0192	0.0043	8.867E-06
0.0754	0.0044	2.716E-65
0.0099	0.0047	3.488E-02
0.0164	0.0057	3.989E-03
-0.1314	0.0129	2.933E-24
0.2567	0.0155	1.611E-61
-0.1024	0.0045	7.240E-115
0.1217	0.0123	3.545E-23
0.0582	0.0043	8.930E-42
0.0070	0.0065	2.833E-01
0.0075	0.0146	6.087E-01
-0.0012	0.0070	8.606E-01
-0.0280	0.0054	2.539E-07
-0.0257	0.0046	2.531E-08
-0.0025	0.0045	5.741E-01
-0.0006	0.0050	8.965E-01

-0.0145	0.0047	1.912E-03
-0.0086	0.0045	5.503E-02
0.0091	0.0147	5.349E-01
0.0016	0.0053	7.705E-01
-0.0449	0.0043	1.343E-25
-0.0507	0.0043	3.066E-32
-0.0252	0.0092	6.122E-03
0.0069	0.0153	6.518E-01
-0.0479	0.0053	1.139E-19
0.0665	0.0045	1.212E-48
0.0666	0.0046	9.701E-48
0.0429	0.0043	7.947E-24
-0.1184	0.0055	2.320E-104
-0.1013	0.0052	5.025E-85
0.0380	0.0071	6.947E-08
0.0624	0.0212	3.276E-03
0.0062	0.0048	1.952E-01
-0.0044	0.0137	7.484E-01
0.0631	0.0043	2.383E-48
-0.0115	0.0107	2.831E-01
0.0701	0.0057	5.922E-35
-0.0477	0.0044	8.549E-28
0.0472	0.0044	2.414E-27
0.0650	0.0043	8.853E-52
-0.0289	0.0174	9.685E-02
0.0015	0.0051	7.770E-01
-0.0377	0.0073	2.518E-07
0.0070	0.0053	1.907E-01
0.0106	0.0130	4.141E-01
-0.0194	0.0053	2.651E-04
-0.0576	0.0045	3.581E-37
0.0175	0.0070	1.275E-02
0.0240	0.0053	5.549E-06
-0.0060	0.0082	4.628E-01
0.0120	0.0149	4.236E-01
0.0486	0.0051	8.470E-22
-0.1021	0.0043	3.070E-125
-0.0007	0.0044	8.825E-01
-0.0008	0.0072	9.153E-01
0.0007	0.0075	9.226E-01
-0.0799	0.0057	5.122E-45
0.0184	0.0052	3.671E-04
-0.0431	0.0043	6.831E-24
0.0439	0.0043	2.384E-24
-0.0136	0.0043	1.631E-03
-0.0401	0.0058	3.327E-12
-0.0269	0.0066	4.976E-05
-0.0112	0.0051	2.993E-02

-0.0538	0.0043	1.903E-36
0.0043	0.0046	3.406E-01
0.0270	0.0043	3.291E-10
-0.0056	0.0061	3.614E-01
0.0034	0.0068	6.136E-01
-0.0116	0.0054	3.017E-02
0.0071	0.0058	2.212E-01
-0.0733	0.0060	1.721E-34
0.0874	0.0064	7.498E-42
0.0050	0.0128	6.972E-01
0.0469	0.0073	1.008E-10
0.0442	0.0117	1.487E-04
-0.1000	0.0074	6.214E-42
-0.0998	0.0073	3.177E-42
0.0621	0.0063	1.047E-22
-0.0425	0.0199	3.291E-02
-0.0542	0.0064	3.098E-17
0.0135	0.0128	2.913E-01
-0.0910	0.0191	1.802E-06
-0.0712	0.0066	3.148E-27
0.0688	0.0065	3.560E-26
-0.0711	0.0074	5.525E-22
0.0083	0.0122	4.953E-01
0.0253	0.0065	1.038E-04
0.0351	0.0124	4.716E-03
0.0019	0.0088	8.311E-01
0.0397	0.0076	1.591E-07
-0.0394	0.0079	6.491E-07
0.0201	0.0073	5.969E-03
0.0278	0.0069	5.096E-05
0.0088	0.0072	2.207E-01
-0.0109	0.0114	3.355E-01
0.0593	0.0063	3.141E-21
-0.0566	0.0063	1.557E-19
-0.0721	0.0131	3.326E-08
0.0144	0.0072	4.435E-02
0.0828	0.0067	3.112E-35
0.0180	0.0073	1.432E-02
0.1166	0.0081	1.663E-47
0.0418	0.0103	4.879E-05
-0.0226	0.0143	1.144E-01
0.0698	0.0062	4.313E-29
0.0705	0.0063	5.182E-29
0.0388	0.0063	6.422E-10
0.0540	0.0063	1.145E-17
0.0526	0.0063	9.237E-17
-0.0257	0.0065	7.893E-05
-0.0500	0.0080	5.092E-10

-0.0216	0.0077	5.261E-03
0.0414	0.0063	6.898E-11
0.0120	0.0085	1.584E-01
0.0161	0.0069	1.901E-02
0.0169	0.0197	3.914E-01
-0.0160	0.0192	4.042E-01
0.0450	0.0065	4.203E-12
-0.0437	0.0064	6.699E-12
-0.0573	0.0068	5.287E-17
-0.0492	0.0073	1.466E-11
-0.0275	0.0129	3.348E-02
0.0502	0.0063	1.521E-15
-0.0321	0.0076	2.094E-05
0.1165	0.0161	4.312E-13
-0.0160	0.0100	1.075E-01
-0.0335	0.0063	9.622E-08
-0.0870	0.0190	4.629E-06
-0.0267	0.0063	2.599E-05
0.0286	0.0063	6.332E-06
0.0282	0.0095	2.813E-03
0.0205	0.0083	1.380E-02
-0.0897	0.0065	4.831E-43
0.0440	0.0063	2.808E-12
0.0178	0.0118	1.298E-01
-0.1599	0.0088	3.058E-74
0.0959	0.0067	6.561E-47
-0.0370	0.0081	5.168E-06
0.0916	0.0062	5.398E-49
-0.0839	0.0065	8.515E-38
0.0831	0.0064	5.189E-38
0.0482	0.0100	1.550E-06
0.0440	0.0066	2.929E-11
0.0611	0.0062	9.109E-23
-0.0139	0.0083	9.399E-02
-0.0330	0.0112	3.215E-03
-0.0324	0.0159	4.158E-02
0.0401	0.0069	6.188E-09
0.0277	0.0078	3.834E-04
0.0257	0.0103	1.259E-02
0.0277	0.0110	1.180E-02
-0.0075	0.0097	4.394E-01
0.0037	0.0079	6.395E-01
0.0206	0.0081	1.098E-02
-0.0295	0.0143	3.912E-02
-0.0319	0.0074	1.627E-05
-0.0580	0.0089	7.180E-11
-0.0617	0.0089	4.132E-12
0.0890	0.0116	1.688E-14

-0.0179	0.0093	5.426E-02
0.0158	0.0320	6.215E-01
0.0106	0.0246	6.665E-01
-0.0025	0.0099	8.006E-01
-0.0273	0.0070	9.619E-05
-0.0705	0.0215	1.041E-03
-0.0224	0.0090	1.281E-02
0.0247	0.0067	2.273E-04
-0.0188	0.0081	2.029E-02
-0.0187	0.0081	2.096E-02
0.0269	0.0108	1.275E-02
-0.0535	0.0080	2.270E-11
-0.0288	0.0067	1.720E-05
-0.0211	0.0067	1.637E-03
0.0072	0.0116	5.348E-01
0.0345	0.0070	8.283E-07
-0.0226	0.0096	1.856E-02
-0.0174	0.0119	1.437E-01
-0.0163	0.0072	2.358E-02
0.0181	0.0083	2.920E-02
0.0216	0.0080	6.934E-03
-0.0080	0.0078	3.051E-01
-0.0435	0.0069	2.894E-10
-0.0903	0.0233	1.064E-04
-0.0235	0.0068	5.485E-04
0.0274	0.0072	1.415E-04
0.0108	0.0075	1.499E-01
-0.0180	0.0074	1.500E-02
-0.0969	0.0116	6.630E-17
0.0036	0.0269	8.935E-01
-0.0590	0.0066	3.913E-19
-0.0199	0.0071	5.066E-03
-0.0169	0.0084	4.423E-02
-0.0465	0.0379	2.199E-01
-0.0106	0.0087	2.231E-01
-0.0258	0.0074	4.894E-04
-0.0439	0.0089	8.115E-07
-0.0029	0.0070	6.787E-01
-0.0553	0.0072	1.584E-14
-0.0590	0.0074	1.549E-15
0.0321	0.0071	6.151E-06
0.0293	0.0071	3.679E-05
0.0485	0.0098	7.460E-07
0.0272	0.0067	4.914E-05
0.0031	0.0085	7.153E-01
0.0298	0.0167	7.435E-02
0.0319	0.0119	7.347E-03
0.0105	0.0068	1.226E-01

0.0327	0.0098	8.477E-04
0.0334	0.0108	1.984E-03
-0.0120	0.0072	9.558E-02
-0.0185	0.0084	2.764E-02
0.0190	0.0069	5.894E-03
-0.0103	0.0073	1.583E-01
-0.0094	0.0071	1.855E-01
0.0367	0.0066	2.688E-08
0.0373	0.0067	2.589E-08
-0.0184	0.0067	6.028E-03
-0.0044	0.0088	6.171E-01
-0.0122	0.0092	1.848E-01
0.0552	0.0118	2.897E-06
-0.0112	0.0108	2.997E-01
0.0299	0.0080	1.859E-04
-0.0319	0.0069	3.779E-06
0.0203	0.0070	3.732E-03
0.0303	0.0078	1.025E-04
0.0745	0.0120	5.355E-10
0.0433	0.0179	1.556E-02
0.0076	0.0087	3.824E-01
-0.0469	0.0127	2.217E-04
-0.0464	0.0127	2.586E-04
-0.0185	0.0069	7.337E-03
-0.0150	0.0072	3.722E-02
-0.0462	0.0069	2.147E-11
0.1110	0.0178	4.490E-10
-0.0224	0.0112	4.550E-02
0.0134	0.0067	4.550E-02
0.0234	0.0072	1.154E-03
0.1096	0.0088	1.321E-35
0.1103	0.0089	2.843E-35
-0.0966	0.0095	2.743E-24
0.0140	0.0115	2.235E-01
-0.0153	0.0094	1.036E-01
0.0073	0.0069	2.901E-01
-0.0157	0.0076	3.885E-02
-0.0168	0.0077	2.912E-02
-0.0345	0.0090	1.264E-04
0.0067	0.0104	5.194E-01
-0.0461	0.0251	6.626E-02
-0.0079	0.0077	3.049E-01
0.0797	0.0070	4.926E-30
0.0135	0.0083	1.038E-01
0.0170	0.0076	2.530E-02
-0.0398	0.0074	7.516E-08
-0.0392	0.0066	2.861E-09
0.0346	0.0080	1.525E-05

0.0204	0.0070	3.565E-03
-0.0064	0.0075	3.935E-01
0.0109	0.0076	1.515E-01
-0.0070	0.0109	5.207E-01
0.0249	0.0067	2.021E-04
0.0237	0.0067	4.042E-04
0.0442	0.0073	1.406E-09
0.0275	0.0105	8.818E-03
0.0483	0.0081	2.477E-09
-0.0206	0.0068	2.450E-03
-0.0178	0.0074	1.615E-02
0.0105	0.0090	2.433E-01
-0.0168	0.0070	1.640E-02
-0.1233	0.0072	9.661E-66
-0.1233	0.0072	9.661E-66
0.0509	0.0088	7.291E-09
-0.0402	0.0076	1.227E-07
-0.0367	0.0085	1.577E-05
0.0995	0.0075	3.613E-40
0.0998	0.0074	1.878E-41
-0.1042	0.0192	5.728E-08
-0.0164	0.0069	1.746E-02
-0.0050	0.0114	6.610E-01
0.0177	0.0087	4.190E-02
0.0043	0.0069	5.332E-01
0.0170	0.0069	1.375E-02
-0.0161	0.0068	1.790E-02
-0.0155	0.0070	2.681E-02
0.0660	0.0083	1.838E-15
-0.0235	0.0098	1.649E-02
-0.0220	0.0094	1.926E-02
-0.0068	0.0076	3.709E-01
0.0219	0.0069	1.504E-03
0.2048	0.0249	1.954E-16
0.0849	0.0304	5.226E-03
-0.0059	0.0076	4.376E-01
-0.0148	0.0085	8.165E-02
0.0009	0.0078	9.081E-01
0.0131	0.0071	6.503E-02
-0.0010	0.0067	8.814E-01
-0.0333	0.0085	8.942E-05
-0.0184	0.0150	2.199E-01
0.0284	0.0073	1.001E-04
-0.0272	0.0134	4.237E-02
0.0117	0.0076	1.237E-01
0.0141	0.0075	6.011E-02
-0.0287	0.0080	3.339E-04
-0.0052	0.0085	5.407E-01

0.0053	0.0078	4.968E-01
-0.0253	0.0383	5.089E-01
0.0275	0.0085	1.215E-03
0.0405	0.0068	2.587E-09
-0.0173	0.0067	9.820E-03
-0.0331	0.0077	1.718E-05
0.0308	0.0071	1.438E-05
-0.0089	0.0095	3.488E-01
0.0095	0.0066	1.500E-01
0.0124	0.0075	9.826E-02
-0.0737	0.0066	5.937E-29
-0.0740	0.0066	3.556E-29
-0.0047	0.0069	4.958E-01
-0.0003	0.0067	9.643E-01
0.0294	0.0067	1.144E-05
0.0377	0.0067	1.835E-08
0.0094	0.0066	1.544E-01
-0.0005	0.0092	9.567E-01
-0.0175	0.0071	1.371E-02
-0.0389	0.0067	6.399E-09
-0.0036	0.0072	6.171E-01
0.0660	0.0123	8.057E-08
-0.0203	0.0070	3.732E-03
-0.0249	0.0073	6.473E-04
-0.0188	0.0073	1.001E-02
0.0107	0.0104	3.036E-01
0.0091	0.0233	6.961E-01
-0.0015	0.0067	8.229E-01
-0.0008	0.0067	9.050E-01
0.0156	0.0074	3.502E-02
-0.1616	0.0066	2.140E-132
-0.1923	0.0067	3.660E-181
0.0350	0.0077	5.482E-06
0.0728	0.0085	1.084E-17
-0.0484	0.0072	1.790E-11
0.0770	0.0093	1.236E-16
0.0025	0.0175	8.864E-01
0.0039	0.0068	5.663E-01
-0.0688	0.0126	4.753E-08
-0.0298	0.0122	1.458E-02
-0.0097	0.0078	2.137E-01
-0.0339	0.0117	3.762E-03
0.0346	0.0068	3.614E-07
0.0317	0.0079	6.004E-05
0.0317	0.0079	6.004E-05
0.0926	0.0111	7.283E-17
-0.0333	0.0079	2.496E-05
0.0751	0.0077	1.787E-22

-0.0410	0.0105	9.432E-05
0.0232	0.0076	2.268E-03
-0.0038	0.0072	5.977E-01
0.0076	0.0077	3.236E-01
0.0077	0.0077	3.173E-01
-0.0219	0.0068	1.279E-03
0.0150	0.0072	3.722E-02
-0.0432	0.0075	8.411E-09
-0.0195	0.0070	5.341E-03
0.0022	0.0116	8.496E-01
-0.0070	0.0479	8.838E-01
-0.0075	0.0098	4.441E-01
0.0595	0.0083	7.573E-13
-0.0073	0.0075	3.304E-01
-0.0515	0.0074	3.416E-12
-0.0469	0.0067	2.560E-12
0.0688	0.0166	3.404E-05
0.0640	0.0111	8.129E-09
-0.0472	0.0070	1.553E-11
0.0498	0.0067	1.063E-13
0.0480	0.0069	3.488E-12
-0.0332	0.0073	5.417E-06
-0.0385	0.0068	1.498E-08
0.0146	0.0075	5.157E-02
0.0482	0.0083	6.352E-09
-0.0439	0.0092	1.826E-06
-0.0269	0.0087	1.988E-03
-0.0275	0.0087	1.573E-03
0.0286	0.0072	7.121E-05
-0.0245	0.0084	3.538E-03
-0.0417	0.0069	1.508E-09
-0.0436	0.0067	7.643E-11
-0.0608	0.0070	3.764E-18
-0.0644	0.0072	3.738E-19
-0.0641	0.0072	5.447E-19
-0.0023	0.0073	7.527E-01
0.0229	0.0112	4.089E-02
0.0227	0.0073	1.873E-03
0.0557	0.0080	3.343E-12
-0.0461	0.0071	8.417E-11
0.0331	0.0072	4.282E-06
-0.0002	0.0143	9.888E-01
0.0178	0.0150	2.354E-01
-0.0134	0.0078	8.581E-02
-0.0169	0.0117	1.486E-01
-0.0100	0.0081	2.170E-01
-0.0311	0.0075	3.374E-05
0.0670	0.0074	1.378E-19

-0.0673	0.0070	6.959E-22
-0.0266	0.0107	1.292E-02
-0.0014	0.0090	8.764E-01
-0.0603	0.0080	4.791E-14
0.0409	0.0071	8.383E-09
-0.0450	0.0163	5.767E-03
0.0657	0.0099	3.215E-11
0.0302	0.0136	2.638E-02
-0.0480	0.0079	1.233E-09
-0.0697	0.0071	9.523E-23
0.1165	0.0109	1.158E-26
0.0013	0.0069	8.506E-01
-0.0269	0.0070	1.216E-04
-0.0294	0.0075	8.855E-05
0.0042	0.0175	8.103E-01
-0.0675	0.0124	5.223E-08
-0.0431	0.0215	4.500E-02
-0.0280	0.0092	2.339E-03
-0.0473	0.0067	1.669E-12
-0.0488	0.0136	3.329E-04
-0.0471	0.0067	2.068E-12
0.0407	0.0281	1.475E-01
0.0145	0.0070	3.832E-02
-0.0033	0.0120	7.833E-01
0.0208	0.0070	2.964E-03
-0.0121	0.0084	1.497E-01
-0.0124	0.0081	1.258E-01
0.0297	0.0105	4.676E-03
-0.0338	0.0198	8.781E-02
-0.0376	0.0197	5.631E-02
0.0116	0.0146	4.269E-01
0.0112	0.0147	4.461E-01
0.0202	0.0067	2.570E-03
-0.0417	0.0071	4.274E-09
-0.0423	0.0071	2.557E-09
0.0455	0.0068	2.214E-11
0.0395	0.0072	4.109E-08
-0.0721	0.0067	5.249E-27
-0.0654	0.0087	5.594E-14
-0.1655	0.1067	1.209E-01
-0.0104	0.0091	2.531E-01
-0.0432	0.0078	3.051E-08
-0.0211	0.0128	9.926E-02
-0.1339	0.0216	5.680E-10
0.0298	0.0079	1.618E-04
0.0416	0.0072	7.569E-09
0.0417	0.0072	6.969E-09
0.0268	0.0077	5.004E-04

0.0109	0.0066	9.863E-02
0.0271	0.0083	1.094E-03
-0.0416	0.0076	4.408E-08
-0.0317	0.0074	1.837E-05
-0.0302	0.0075	5.657E-05
0.0252	0.0076	9.138E-04
0.0152	0.0076	4.550E-02
-0.0107	0.0095	2.600E-01
-0.0670	0.0070	1.054E-21
-0.0655	0.0067	1.426E-22
-0.0274	0.0078	4.434E-04
-0.0256	0.0081	1.575E-03
-0.0270	0.0081	8.581E-04
0.0095	0.0105	3.656E-01
-0.0002	0.0079	9.798E-01
-0.0215	0.0077	5.235E-03
0.0022	0.0098	8.224E-01
0.1829	0.0067	4.420E-164
0.1823	0.0067	5.090E-163
-0.0594	0.0070	2.144E-17
-0.0160	0.0074	3.061E-02
0.0032	0.0068	6.379E-01
-0.0295	0.0068	1.436E-05
0.0044	0.0072	5.411E-01
0.0927	0.0148	3.764E-10
0.0967	0.0148	6.413E-11
0.0750	0.0071	4.405E-26
0.0121	0.0074	1.020E-01
-0.0268	0.0074	2.928E-04
-0.0122	0.0084	1.464E-01
-0.0035	0.0120	7.705E-01
0.0161	0.0108	1.360E-01
0.0109	0.0071	1.247E-01
0.0130	0.0144	3.666E-01
0.0180	0.0099	6.904E-02
-0.0089	0.0077	2.477E-01
0.0228	0.0203	2.614E-01
0.0235	0.0203	2.470E-01
-0.0255	0.0069	2.193E-04
-0.0424	0.0067	2.478E-10
-0.0428	0.0067	1.680E-10
-0.1327	0.0131	4.076E-24
-0.1427	0.0138	4.617E-25
-0.1144	0.0069	9.770E-62
-0.0643	0.0099	8.306E-11
-0.0198	0.0072	5.960E-03
0.0216	0.0097	2.596E-02
-0.0334	0.0081	3.732E-05

-0.0295	0.0084	4.449E-04
0.0252	0.0069	2.600E-04
-0.0456	0.0070	7.304E-11
0.0489	0.0129	1.502E-04
-0.0230	0.0111	3.826E-02
0.0143	0.0104	1.691E-01
0.0461	0.0067	5.960E-12
0.0597	0.0068	1.643E-18
-0.0394	0.0074	1.013E-07
-0.0253	0.0067	1.593E-04
0.1273	0.0072	5.921E-70
0.1682	0.0095	3.819E-70
0.0473	0.0073	9.206E-11
0.0449	0.0072	4.486E-10
-0.0242	0.0090	7.169E-03
0.0503	0.0137	2.411E-04
0.0216	0.0106	4.158E-02
0.0160	0.0137	2.429E-01
-0.2475	0.0224	2.214E-28
-0.2742	0.0166	2.720E-61
-0.2754	0.0166	8.183E-62
0.0549	0.0226	1.513E-02
0.0210	0.0222	3.442E-01
-0.0112	0.0070	1.096E-01
-0.0221	0.0126	7.944E-02
0.0560	0.0087	1.220E-10
-0.0595	0.0071	5.280E-17
0.0433	0.0068	1.919E-10
0.0436	0.0068	1.438E-10
-0.0052	0.0099	5.994E-01
-0.0150	0.0069	2.971E-02
0.1003	0.0123	3.507E-16
-0.1021	0.0123	1.034E-16
0.0187	0.0186	3.147E-01
-0.0275	0.0075	2.457E-04
0.1156	0.0185	4.140E-10
-0.0486	0.0168	3.818E-03
0.1354	0.0126	6.187E-27
-0.0854	0.0110	8.253E-15
-0.0840	0.0091	2.687E-20
0.0217	0.0077	4.830E-03
0.1996	0.0072	3.770E-169
-0.1982	0.0072	8.170E-167
-0.0518	0.0099	1.674E-07
-0.0340	0.0069	8.327E-07
0.0728	0.0069	5.041E-26
0.0735	0.0069	1.703E-26
0.0422	0.0066	1.617E-10

-0.0047	0.0070	5.019E-01
0.0231	0.0073	1.554E-03
0.0003	0.0074	9.677E-01
0.0283	0.0086	9.994E-04
0.0252	0.0082	2.118E-03
-0.0089	0.0092	3.333E-01
-0.0236	0.0087	6.675E-03
0.0324	0.0086	1.649E-04
0.0298	0.0083	3.302E-04
0.0137	0.0075	6.775E-02
0.1399	0.0150	1.093E-20
0.1402	0.0150	9.045E-21
-0.0097	0.0087	2.649E-01
-0.0066	0.0068	3.318E-01
-0.0068	0.0069	3.244E-01
0.0266	0.0070	1.447E-04
0.0249	0.0075	9.002E-04
0.0332	0.0075	9.570E-06
-0.0394	0.0098	5.810E-05
-0.0296	0.0173	8.708E-02
-0.0332	0.0072	4.005E-06
-0.0210	0.0070	2.700E-03
0.0055	0.0073	4.512E-01
-0.0191	0.0287	5.057E-01
-0.0376	0.0200	6.011E-02
0.0259	0.0101	1.034E-02
-0.0208	0.0070	2.964E-03
0.0665	0.0071	7.519E-21
0.0663	0.0071	9.814E-21
0.0250	0.0067	1.905E-04
-0.1073	0.0091	4.333E-32
-0.0088	0.0086	3.062E-01
-0.0106	0.0087	2.231E-01
-0.0145	0.0067	3.045E-02
-0.0006	0.0066	9.276E-01
-0.0078	0.0070	2.652E-01
-0.0436	0.0081	7.337E-08
0.0044	0.0080	5.823E-01
-0.0014	0.0102	8.908E-01
-0.0443	0.0101	1.154E-05
0.0561	0.0071	2.758E-15
-0.0367	0.0102	3.206E-04
0.0034	0.0083	6.821E-01
-0.0425	0.0068	4.105E-10
0.0241	0.0077	1.749E-03
0.0195	0.0107	6.839E-02
-0.0006	0.0158	9.697E-01
0.0308	0.0073	2.452E-05

-0.0141	0.0123	2.517E-01
-0.0369	0.0067	3.640E-08
0.0324	0.0096	7.382E-04
-0.0534	0.0079	1.385E-11
0.0227	0.0106	3.223E-02
-0.0221	0.0081	6.364E-03
0.0471	0.0074	1.955E-10
-0.0300	0.0072	3.091E-05
0.0158	0.0098	1.069E-01
-0.0148	0.0213	4.872E-01
0.0274	0.0103	7.810E-03
-0.0097	0.0087	2.649E-01
0.0414	0.0070	3.333E-09
0.0637	0.0069	2.659E-20
0.0635	0.0067	2.602E-21
0.0177	0.0092	5.437E-02
0.0242	0.0130	6.267E-02
0.0777	0.0091	1.360E-17
-0.0792	0.0091	3.224E-18
0.0095	0.0076	2.113E-01
0.0321	0.0075	1.869E-05
0.0344	0.0068	4.219E-07
0.0344	0.0068	4.219E-07
0.0136	0.0067	4.237E-02
0.0091	0.0069	1.872E-01
0.0171	0.0082	3.704E-02
0.0889	0.0076	1.315E-31
0.0775	0.0090	7.236E-18
-0.0041	0.0073	5.744E-01
0.0055	0.0067	4.117E-01
0.0073	0.0097	4.517E-01
-0.0276	0.0156	7.686E-02
0.0131	0.0069	5.762E-02
0.0216	0.0069	1.745E-03
-0.0071	0.0095	4.548E-01
-0.0065	0.0086	4.498E-01
0.0395	0.0066	2.166E-09
0.0396	0.0066	1.973E-09
-0.0364	0.0110	9.360E-04
0.0003	0.0078	9.693E-01
-0.0474	0.0073	8.406E-11
-0.0298	0.0067	8.677E-06
0.0586	0.0090	7.460E-11
-0.0140	0.0091	1.239E-01
0.0154	0.0094	1.014E-01
0.0552	0.0100	3.390E-08
-0.0020	0.0091	8.260E-01
0.0502	0.0068	1.555E-13

-0.0353	0.0069	3.122E-07
0.0403	0.0080	4.717E-07
-0.0747	0.0083	2.257E-19
0.0320	0.0074	1.530E-05
-0.0274	0.0067	4.322E-05
-0.0139	0.0172	4.190E-01
0.0098	0.0067	1.436E-01
0.0589	0.0067	1.482E-18
-0.0409	0.0195	3.596E-02
0.0792	0.0067	3.044E-32
0.0230	0.0140	1.004E-01
0.0436	0.0079	3.410E-08
-0.0094	0.0070	1.793E-01
-0.0067	0.0102	5.113E-01
0.0457	0.0068	1.810E-11
0.0339	0.0069	8.967E-07
-0.0714	0.0067	1.622E-26
-0.0707	0.0067	4.960E-26
-0.0261	0.0113	2.090E-02
0.0588	0.0083	1.397E-12
0.0601	0.0084	8.382E-13
-0.0532	0.0243	2.858E-02
0.0473	0.0070	1.407E-11
0.0269	0.0070	1.216E-04
0.0283	0.0071	6.722E-05
-0.0187	0.0077	1.516E-02
0.0326	0.0082	7.020E-05
0.0192	0.0100	5.486E-02
-0.0131	0.0071	6.503E-02
-0.0343	0.0199	8.478E-02
-0.0280	0.0200	1.615E-01
-0.1303	0.0089	1.550E-48
0.0336	0.0103	1.106E-03
0.0231	0.0069	8.145E-04
-0.0309	0.0075	3.789E-05
-0.0030	0.0083	7.178E-01
0.0423	0.0070	1.514E-09
-0.0299	0.0070	1.942E-05
0.0004	0.0088	9.637E-01
-0.0375	0.0083	6.241E-06
-0.0438	0.0085	2.564E-07
0.0053	0.0081	5.129E-01
-0.0191	0.0068	4.972E-03
-0.0185	0.0082	2.406E-02
-0.1142	0.0115	3.069E-23
0.0189	0.0070	6.934E-03
0.0030	0.0095	7.522E-01
0.0261	0.0092	4.555E-03

0.0073	0.0090	4.173E-01
0.0443	0.0067	3.793E-11
-0.0167	0.0070	1.705E-02
0.0227	0.0068	8.431E-04
0.1202	0.0073	6.465E-61
0.1212	0.0074	2.729E-60
0.0277	0.0070	7.585E-05
-0.0294	0.0067	1.144E-05
-0.0309	0.0081	1.363E-04
0.0259	0.0077	7.692E-04
0.0194	0.0071	6.288E-03
-0.0998	0.0190	1.499E-07
-0.0345	0.0071	1.179E-06
-0.0051	0.0076	5.022E-01
0.0295	0.0074	6.706E-05
0.0107	0.0073	1.427E-01
-0.0398	0.0074	7.516E-08
0.0063	0.0067	3.471E-01
-0.0269	0.0180	1.351E-01
-0.0288	0.0118	1.466E-02
-0.0762	0.0282	6.890E-03
0.0114	0.0089	2.002E-01
-0.0813	0.0079	7.726E-25
-0.0840	0.0078	4.810E-27
0.0007	0.0068	9.180E-01
0.0543	0.0068	1.402E-15
-0.0220	0.0069	1.431E-03
-0.0314	0.0067	2.778E-06
-0.0083	0.0512	8.712E-01
0.0634	0.0115	3.527E-08
0.0368	0.0068	6.241E-08
-0.0434	0.0071	9.797E-10
-0.0352	0.0079	8.362E-06
-0.0353	0.0077	4.553E-06
-0.0401	0.0194	3.873E-02
0.0557	0.0068	2.587E-16
-0.0198	0.0072	5.960E-03
0.0291	0.0117	1.288E-02
0.0893	0.0078	2.386E-30
-0.0723	0.0075	5.419E-22
-0.0327	0.0087	1.709E-04
-0.0331	0.0084	8.132E-05
-0.0048	0.0084	5.677E-01
0.0191	0.0071	7.142E-03
-0.0220	0.0088	1.242E-02
-0.0365	0.0141	9.635E-03
-0.0189	0.0080	1.815E-02
0.0732	0.0089	1.956E-16

-0.0655	0.0069	2.250E-21
0.0522	0.0068	1.635E-14
0.0426	0.0069	6.662E-10
-0.0173	0.0072	1.627E-02
0.0158	0.0095	9.628E-02
0.0340	0.0102	8.581E-04
-0.0226	0.0068	8.889E-04
-0.0238	0.0069	5.621E-04
0.0341	0.0084	4.917E-05
0.0107	0.0076	1.592E-01
-0.0030	0.0067	6.543E-01
-0.0343	0.0067	3.065E-07
-0.0638	0.0184	5.255E-04
0.0319	0.0067	1.925E-06
-0.0070	0.0071	3.242E-01
0.0150	0.0073	3.990E-02
-0.0215	0.0083	9.588E-03
-0.0166	0.0067	1.323E-02
-0.0517	0.0073	1.419E-12
-0.0542	0.0074	2.401E-13
-0.0078	0.0100	4.354E-01
