# Supplementary figures and images for: Validation and genetic heritability estimation of known type 2 diabetes related variants in the Korean population
Source: Genomics Inform. 2021 Dec 31;19(4):e37. doi: 10.5808/gi.21071 (PMC8752982; doi:10.5808/gi.21071)

# Supplementary Figure 1. Effect sizes and MAF of variants (liver enzymes)

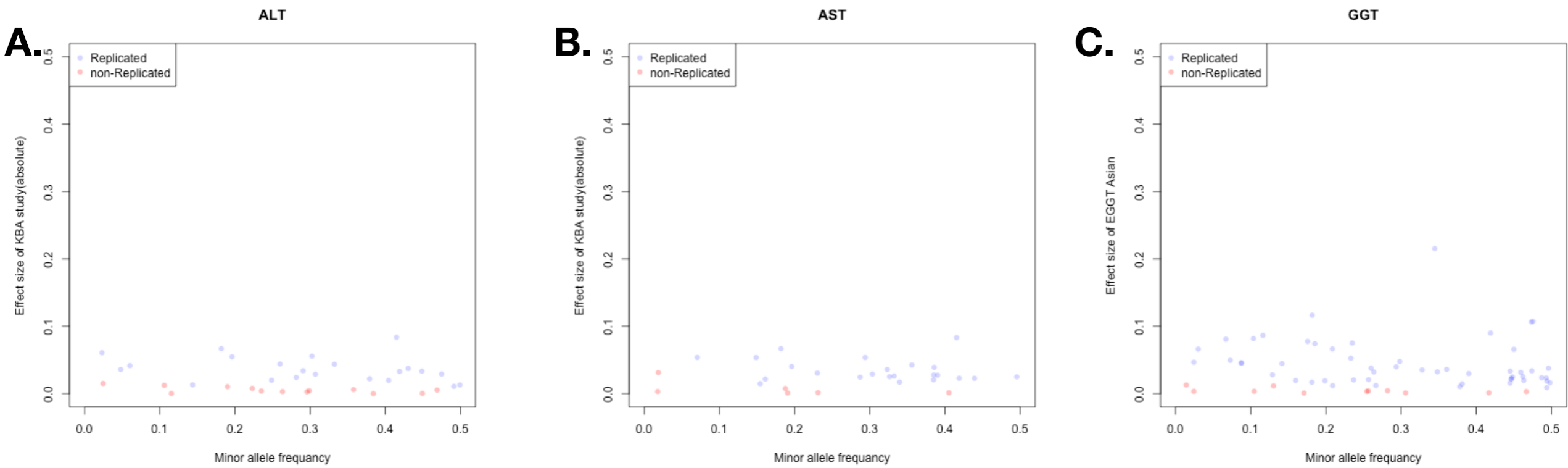

Supplement: Supplementary Fig. 1. — Effect sizes and MAF of variants (liver enzymes) [file gi-21071suppl2.pdf]

# Supplementary Figure 2. Effect sizes and MAF of variants (lipid traits)

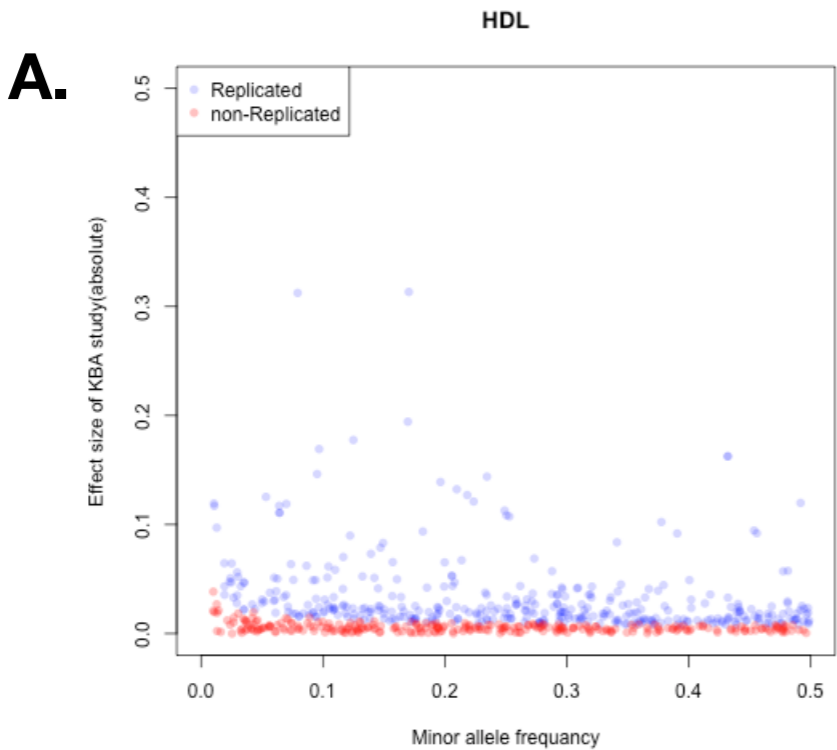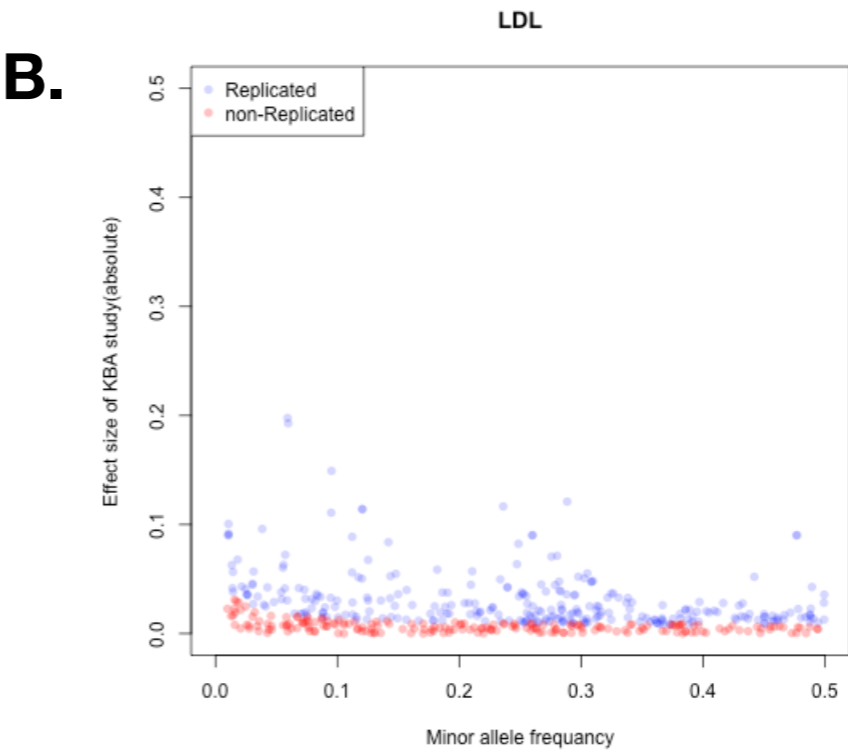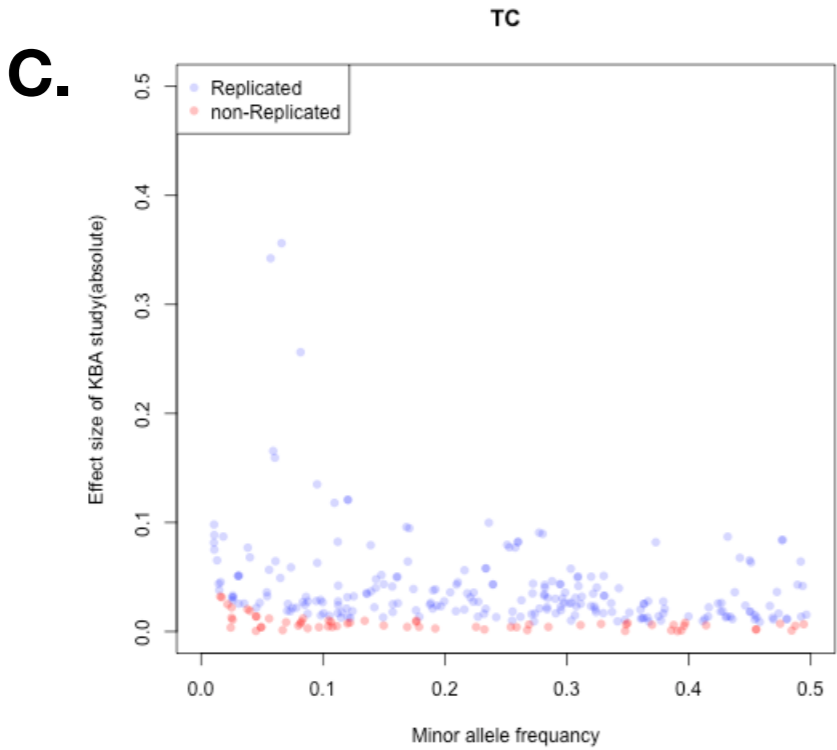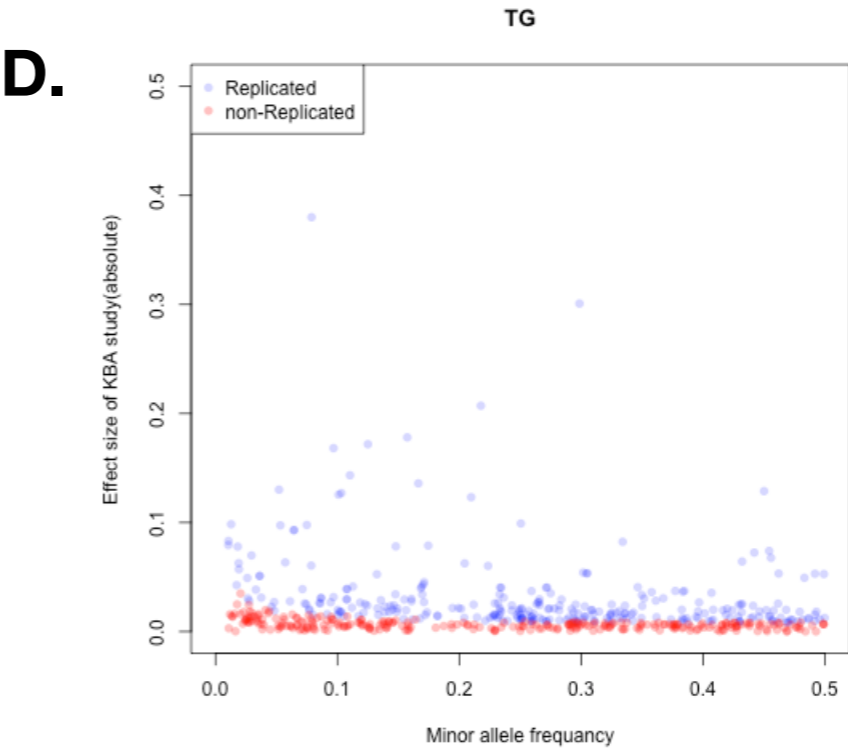

Supplement: Supplementary Fig. 2. — Effect sizes and MAF of variants (lipid traits) [file gi-21071suppl3.pdf]
